# Versatile Dibenzothio[seleno]phenes via Hexadehydro-Diels–Alder Domino Cyclization

**DOI:** 10.3389/fchem.2019.00374

**Published:** 2019-05-24

**Authors:** Baohua Liu, Qiong Hu, Yinshan Wen, Bo Fang, Xiaoliang Xu, Yimin Hu

**Affiliations:** College of Chemistry and Materials Science, Anhui Normal University, Wuhu, China

**Keywords:** cyclization, cross-coupling, hexadehydro-Diels–Alder reaction, dibenzoselenophene, dibenzothiophene, benzyne

## Abstract

A facile strategy to synthesize highly substituted dibenzoselenophenes and dibenzothiophenes by a domino hexadehydro-Diels–Alder reaction is reported in this article. The formation of three new C–C bonds, one new Caryl–Se/Caryl–S bond, and C–H σ-bond migration via one-pot multiterminal cycloaddition reactions were involved in over three transformations. The target tetracyclic compounds were prepared from tetraynes with a triphenylphosphine selenide or triphenylphosphine sulfide. This reaction played a pivotal role in constructing natural thio[seleno]phene cores, which were highly substituted, and is a robust method for producing fused heterocycles.

## Introduction

**Scheme 1:**
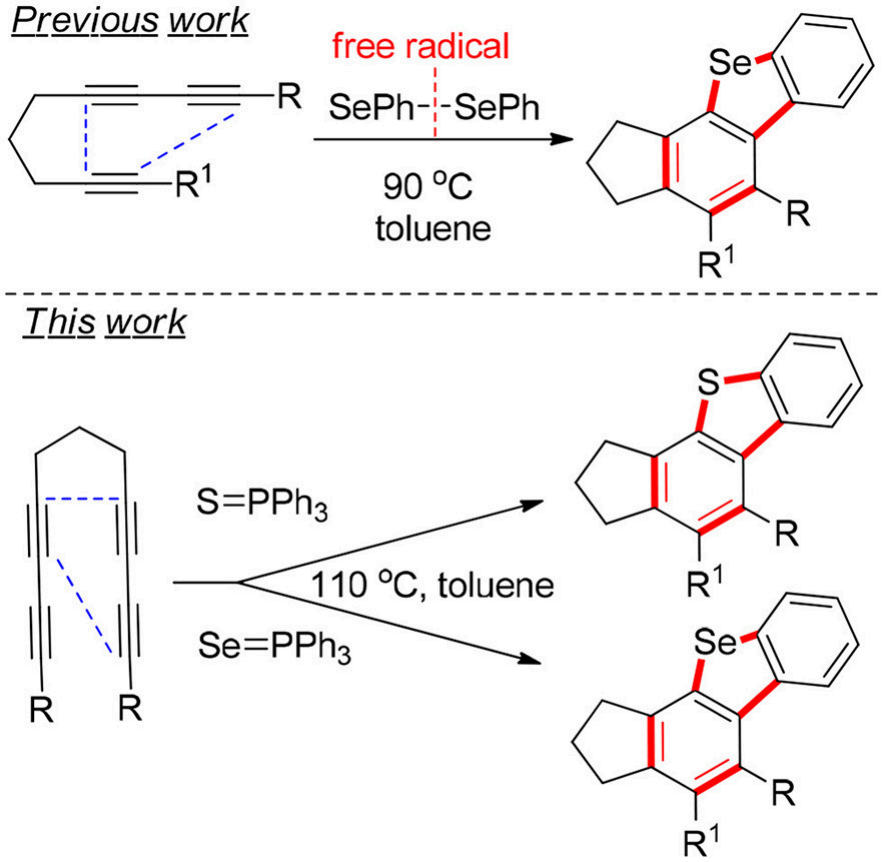
Target tetracyclic thio[seleno]phene core structures.

In recent years, the use of group-16 elements such as sulfur and selenium in biological processes, materials science, and anti-cancer drugs has increasingly attracted interest among researchers (Chen et al., 2013; Sun et al., 2018; Waldvogel et al., 2018). During the manufacturing of perfumes, dyes, polymers, medicine materials, and pharmaceuticals, thiophene and selenophene could be significant intermediates (Duffield-Lillico et al., 2002; Sancineto et al., 2015). Herrmann (2015) used a structure-based medicine design to identify cyclothiophenes as antiprion molecules. Ebata et al. (2007) and Shinamura et al. (2010) presented a facile and efficient synthesis of complex benzoselenophenes. Kumar et al. (2007) established versatile and flexible methods for synthesizing regenerable chain-breaking 2,3-dihydrobenzoselenophene-5-ol antioxidants. Mlochowska reported carbon–carbon bond cleavages, ring closure and ring transformations, syn-eliminations, and sigmatropic [2,3]-rearrangements involving oxygenated and reduced selenium and sulfur compounds (Młochowski et al., 2012). Hoye used benzynes with the Lewis acid BF_3_ in cascade reactions to promote carbene-like reactivity (Shen et al., 2018). We reported (Hu et al., 2017) a novel cyclization method involving a benzyne intermediate/[2+2] cycloaddition/intermolecular rearrangement/σ-bond migration of an aromatic C–H bond and subsequent elimination to prepare rare tetracyclic thio[seleno]phene cores (Wakefield et al., 2007; Tsai et al., 2016). The synthesis of this four-fused-heterocyclic-ring system (Scheme 1) is very challenging for organic chemists (Siebert et al., 2010; Piemontesi et al., 2016; Meng et al., 2017; He et al., 2018). The benzannulation of triynes and triphenylphosphine selenide or triphenylphosphine sulfide in toluene typically yields benzothio[seleno]phene derivatives as the major product that also have high atom economy. When cyclopenta[h]benzothio[seleno]phene derivatives are compared with general thio[seleno]phene derivatives, the former were prepared in current reactions that have multiple rings, and such sophisticated and diverse structures have broad potential for chemical production and pharmaceutical synthesis (Wetzel et al., 2015; Wang et al., 2016).

Herein, we describe a facile strategy for preparing multifunctional dibenzoselenophenes and dibenzothiophenes, which are difficult to obtain via other means. These reactions of tetraynes with triphenylphosphine selenide or triphenylphosphine sulfide produce various fused products in excellent yields without catalysts, oxidants, bases, or metals. This method, with no need for directing groups, however, exhibits excellent regioselectivity and can generate fused 2,3-dihydro-1H-benzoindenoselenophene and 2,3-dihydro-1H-benzoindenothiophene via a one-pot multiterminal HDDA (Ma et al., 2012; Wang et al., 2012; Karmakar et al., 2018) cycloaddition reaction (1a−1q) in excellent yields (Table 1). Consequently, this route could afford an economical, efficient, and direct path of forming highly substituted dibenzoselenophene- and dibenzothiophene-containing compounds. We report, to the best of our knowledge, the first process to provide a range of valuable selenium-containing molecules using a simple and convenient system without metal catalysts or other additive oxidants. The reaction of multiyne 1a with triphenylphosphine selenide was used as a model reaction. When the reaction system temperature increased rapidly to 105°C, the efficiency of the reaction was greatly improved. After the screening of catalysts, we revealed that Pd(OAc)_2_ was the most effective catalyst for the cyclization reaction; amazingly, further investigation showed that the reaction proceeded better without other additives or metal catalysts, such as oxidants or bases. Therefore, the following reaction environments were used in follow-up experiments: 1 equiv of 1 was reacted with 1.05 equiv of triphenylphosphine selenide in toluene at 105°C for 16 h.

**Table 1:** One-pot formation of fused benzoselenophenes^a^, ^b^.

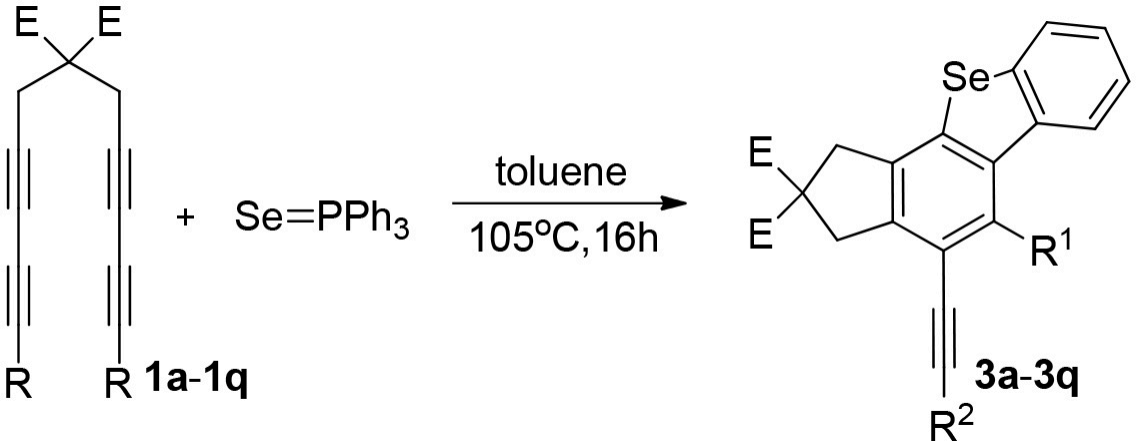		
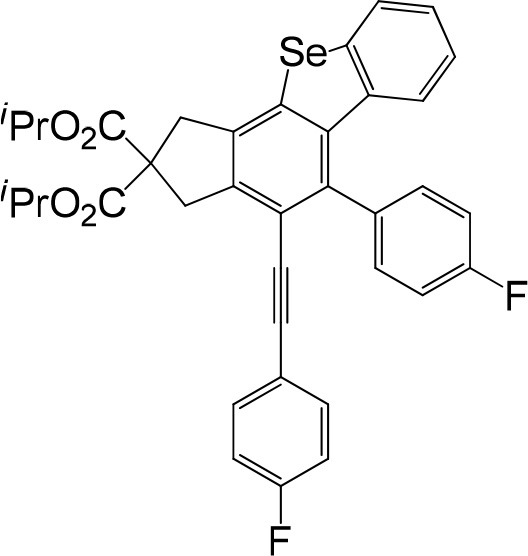 **3a** 83%	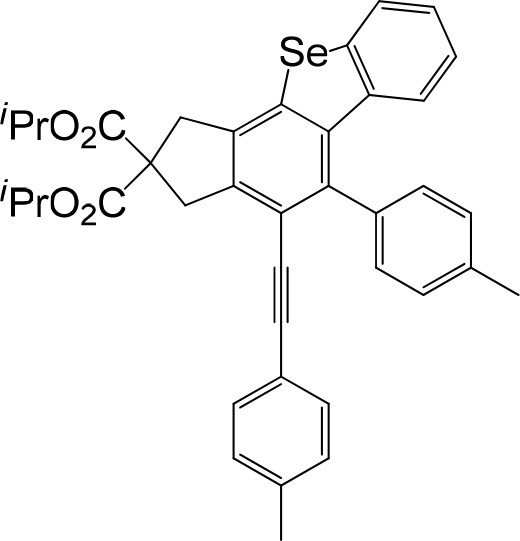 **3b** 87%	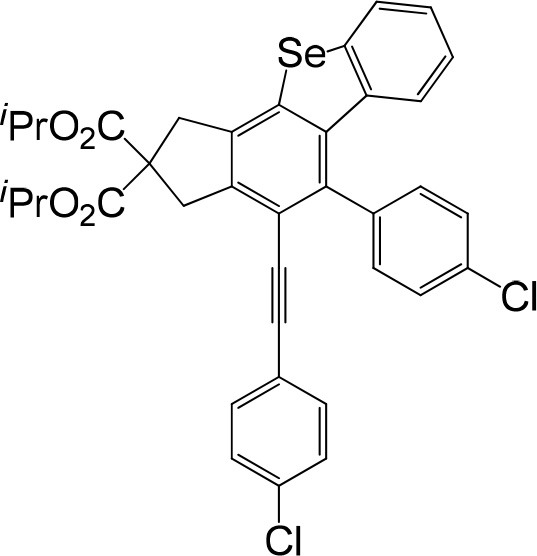 **3c** 81%
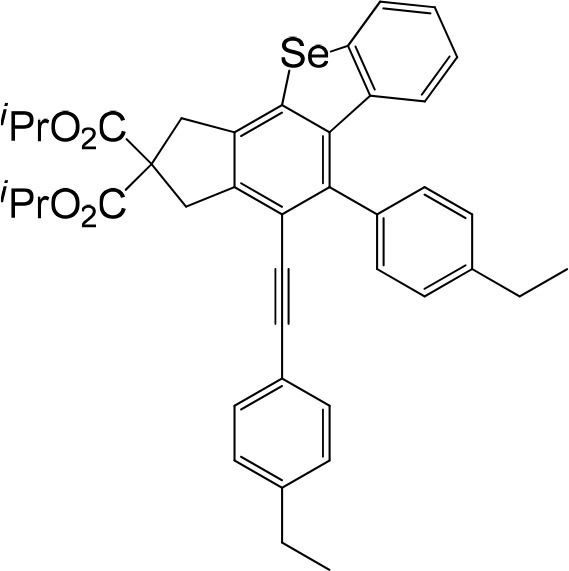 **3d** 78%	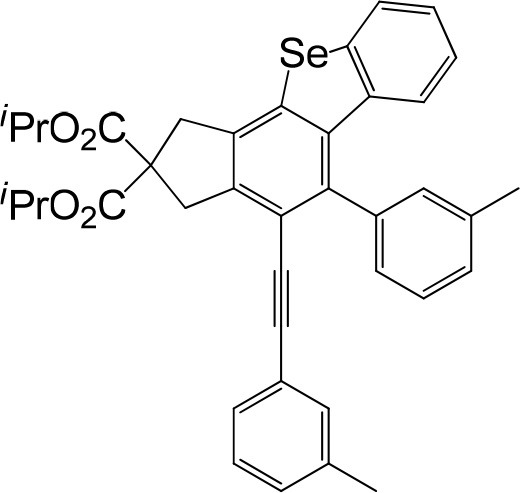 **3e** 78%	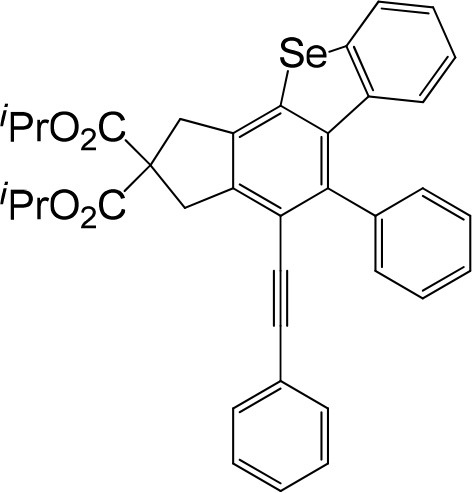 **3f** 80%
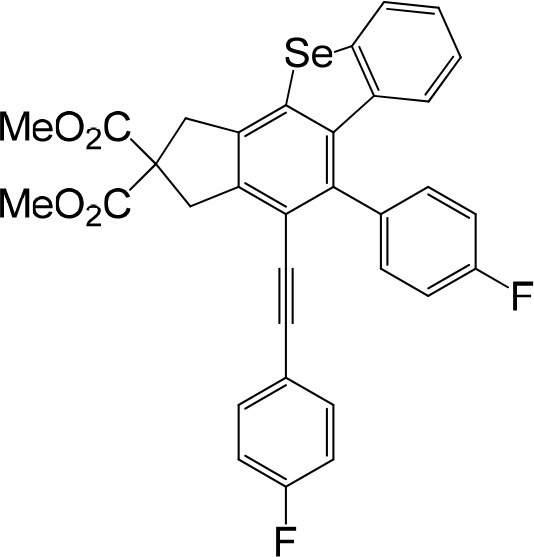 **3g** 79%	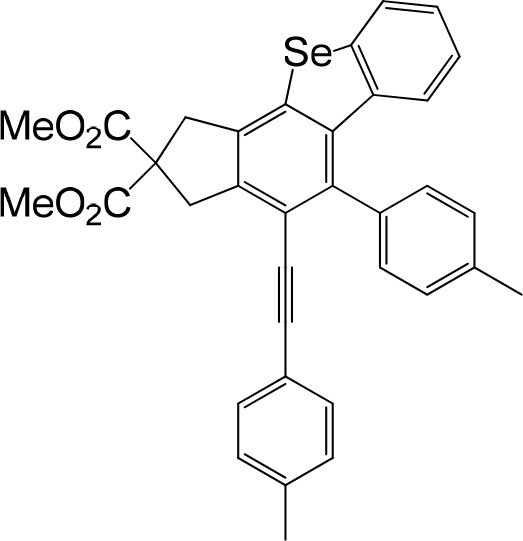 **3h** 80%	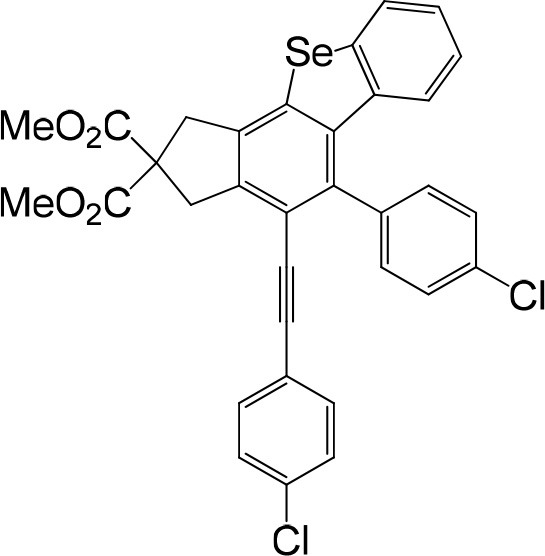 **3i** 72%
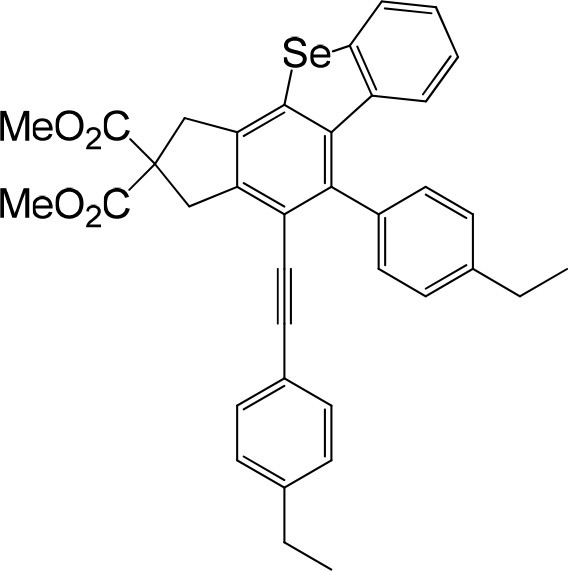 **3j** 79%	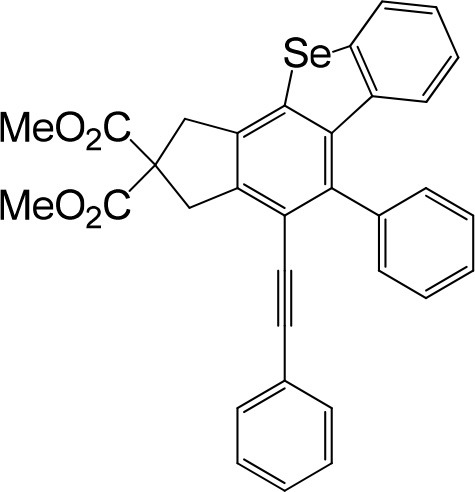 **3k** 80%	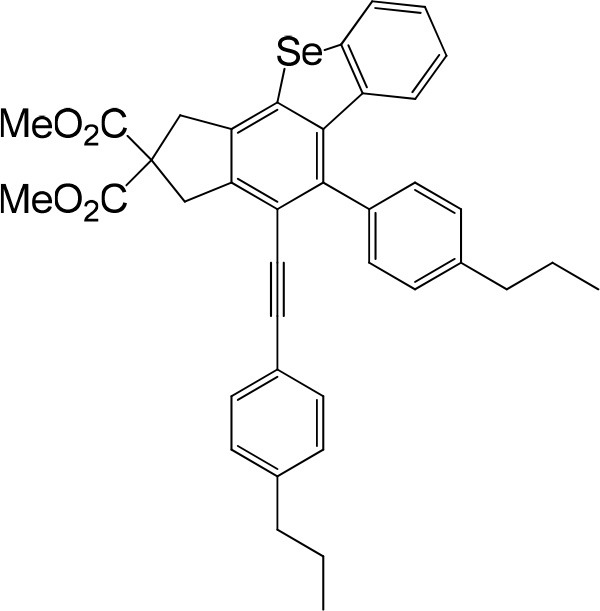 **3l** 72%
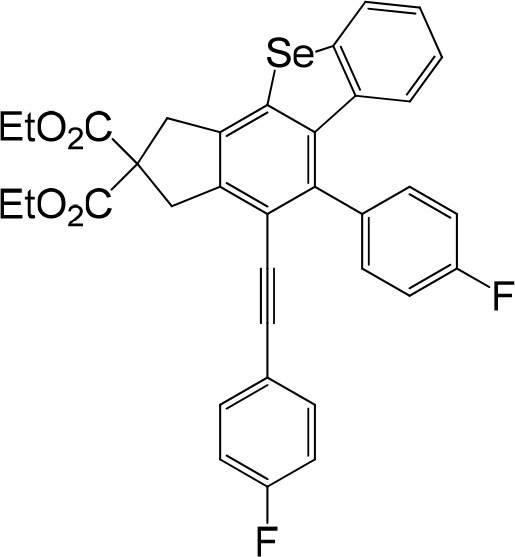 **3m** 82%	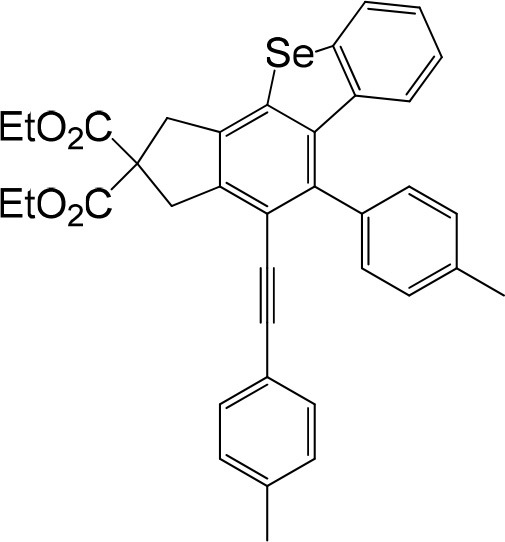 **3n** 75%	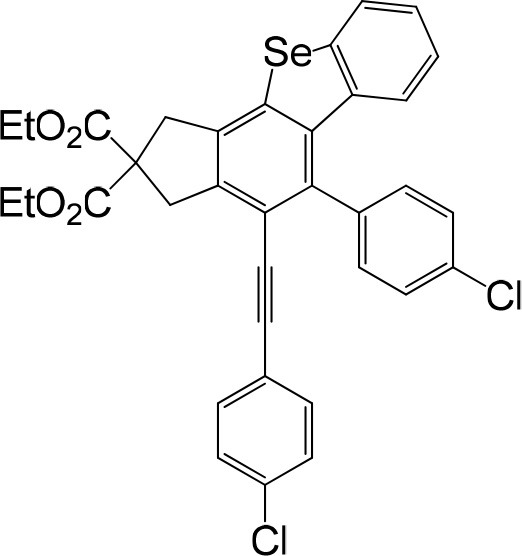 **3o** 81%
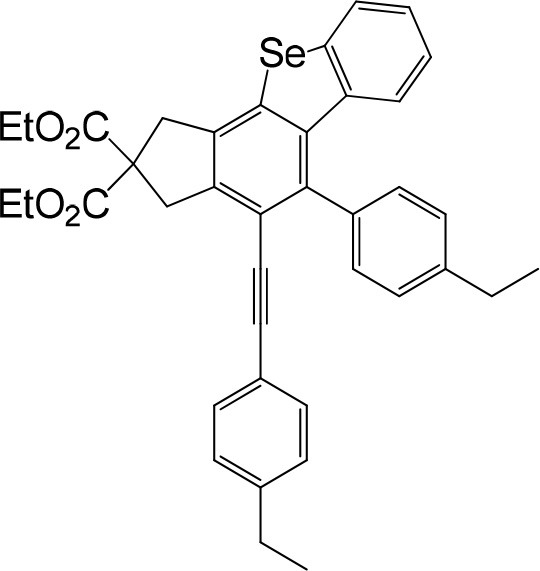 **3p** 74%	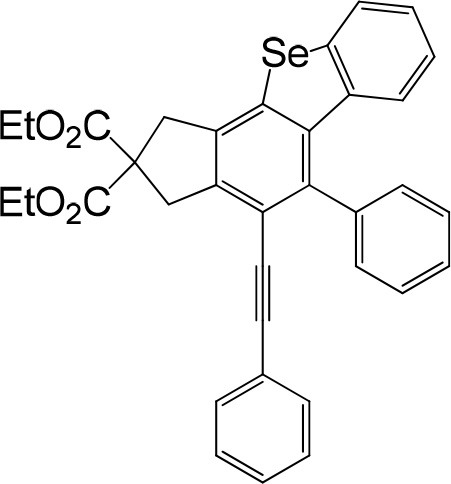 **3q** 80%	

a *Reaction scale: **1a–q** (1.0 equiv.), triphenylphosphine selenide (1.05 equiv.), 105°C, toluene 1.5 mL*.

b *Yield of the isolated product after flash column chromatography*.

## Results and Discussion

We are interested in the application of a Diels-Alder extension, such as the domino aryne scope shown in Table 1. In addition, a variety of tetrayne substrates were able to be applied to this cascade cyclization. Synthetic products ranging from substituted isopropylbenzoindenoselenophene to substituted diethylbenzoindenoselenophene were easy to isolate in high to excellent yields from a variety of multiynes with different substitution patterns. Either electron-withdrawing or electron-donating groups on the aryl ring of the tetraynes could be equally valid; for example, any one of the fluoro, chloro, ethyl, methyl, and propyl groups was applicable to the system. Reactions of diverse tetraynes with triphenylphosphine selenide afforded the dihydrobenzoindenoselenophene skeleton in good yields (e.g., 3a−3c, 3g, 3i, 3m, and 3o were prepared in yields >80%, as shown in Table 1). Among the obtained products, the isolated yield of compound 3b was the highest (87%). The yields of the domino reactions of substituted tetraynes 1a−1q with triphenylphosphine selenide were basically similar (e.g., 3d, 3e, 3h, 3j, 3l, 3n, and 3p were obtained in yields of 70 and 78%, as shown in Table 1). In the formation of highly substituted benzoselenophenes, these results gave evidence of the advantages of the direct hydrocarbon functionalization of unsaturated alkynes with triphenylphosphine selenide.

**Figure 1:**
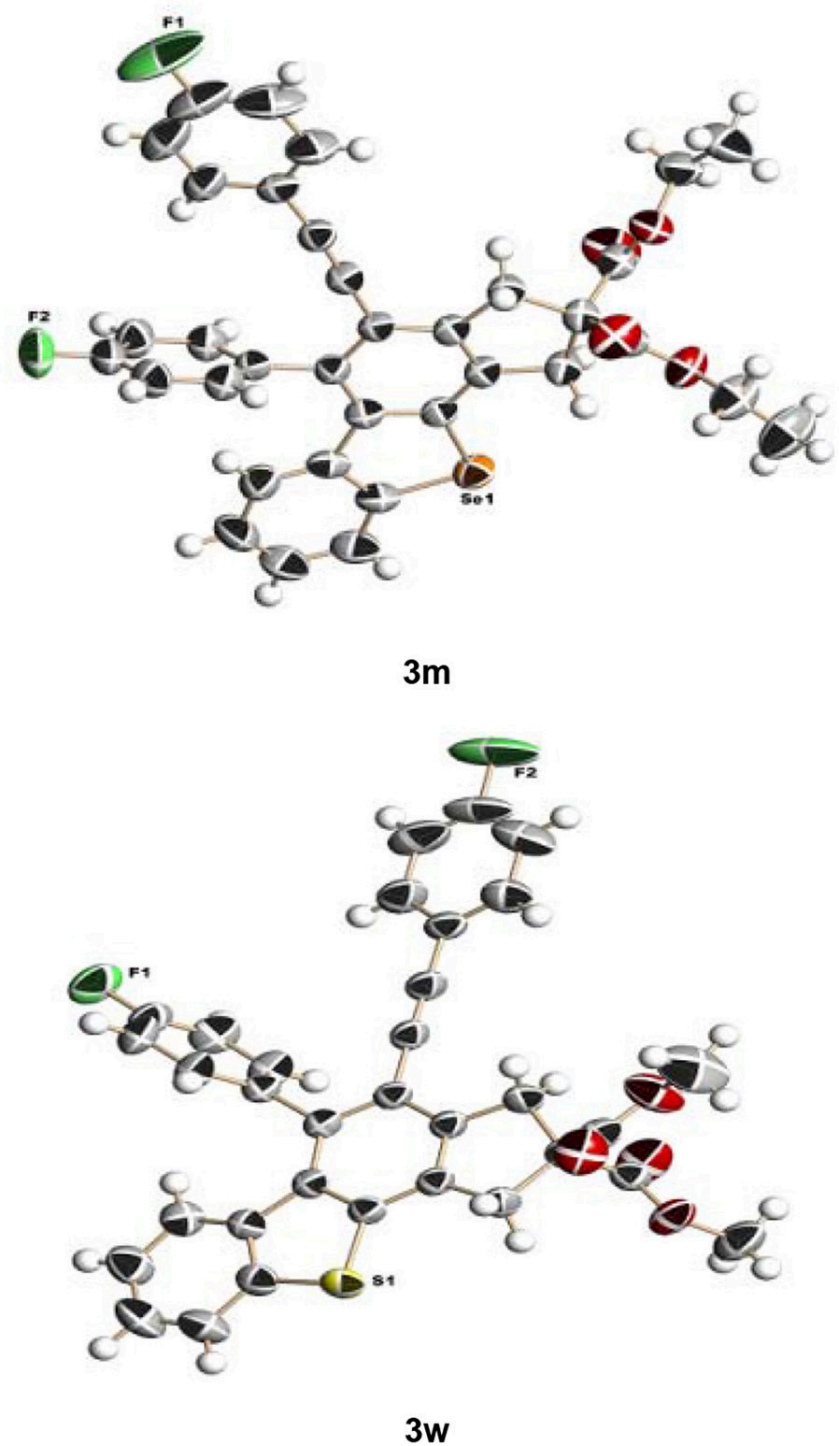
Fused benzothio[seleno]phene molecular structures of 3m and 3w.

In consideration of the high reactivity of arynes, we investigated several HDDA reactions with triphenylphosphine sulfide. Interestingly, highly substituted 5-phenyl-4-(phenylethynyl)-1H-benzoindenothiophene was formed in a high yield. The representative results are shown in Table 2. Substituents including p-fluorine (82%), hydrogen (80%), or p-methyl (83%) on the aryl rings of the tetraynes were extremely well-tolerated in the reaction with triphenylphosphine sulfide to produce the corresponding products with high yields (3s, 3v, and 3w). When substrates containing Me groups (Table 2, 3s−3u) instead of ^i^Pr groups (3r) were reacted with triphenylphosphine sulfide at the same temperature, those compounds of polyfunctionalized 5-phenyl-4-(phenylethynyl)-1H-benzoindenothiophene-2,2-dicarboxylate were isolated in good to high yields. The alkyne-functionalized tetracyclic thiophenes were believed to build via a benzyne intermediate followed by formal [2+2] cycloaddition and C–H migration. The overall reaction that produced highly substituted fused thiophenes proved excellent regioselectivity. The molecular structures of 3 m and 3 w were confirmed by X-ray diffraction (Figure 1).

**Table 2:** One-pot formation of highly substituted fused benzothiophenes^a^, ^b^.

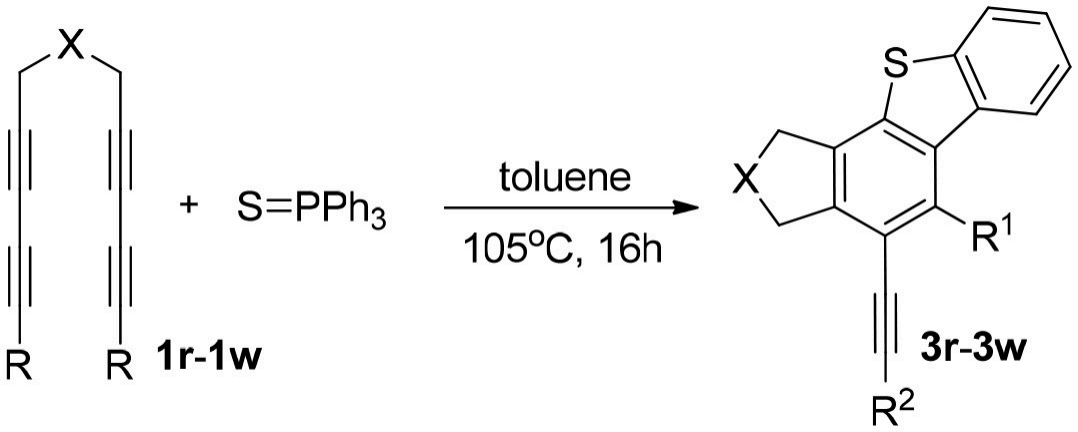
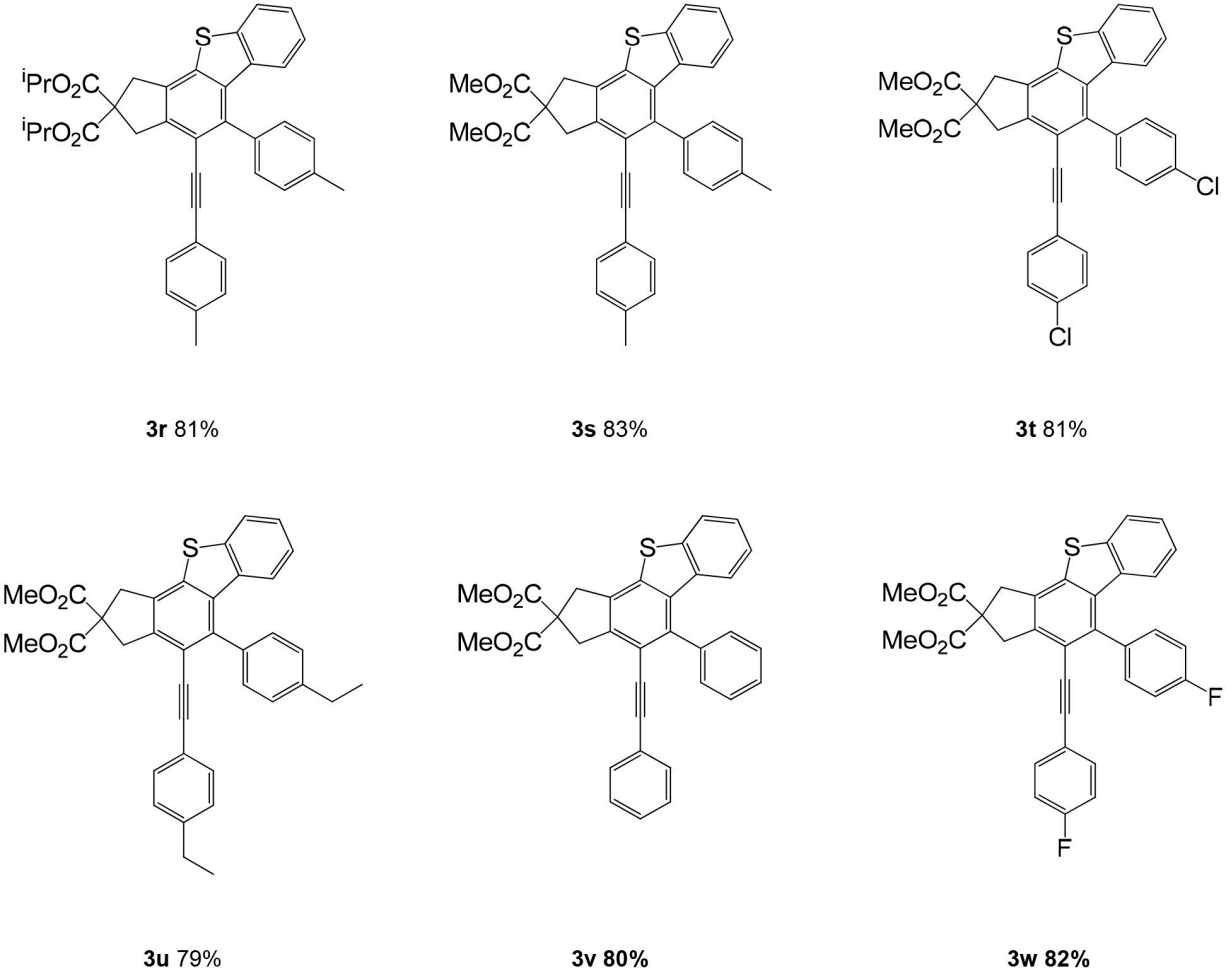

a *Reaction scale: **1a–h** (1.0 equiv), triphenylphosphine sulfide (1.05 equiv), 105°C, toluene 1.5 mL*.

b *Yield of the isolated product after flash column chromatography*.

**Scheme 2:**
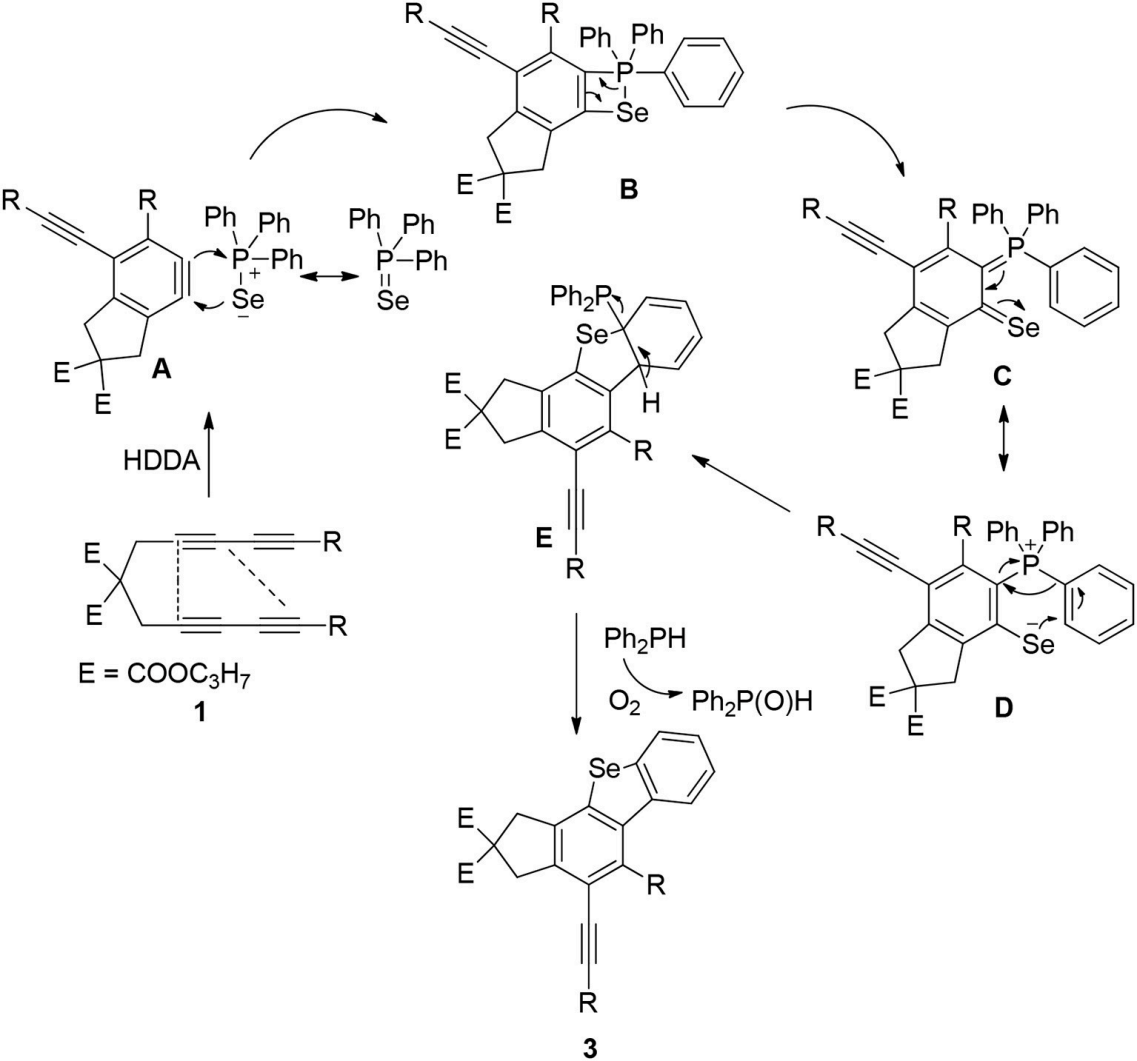
Mechanisms of fused benzoselenophene derivatives.

Scheme 2 shows the sequence of procedures involved in the formation of Caryl–S(Se)–Caryl and C–C bonds containing benzyne cycloaddition and an intramolecular C–H migration cascade. Aryne intermediate A, which is generated via the HDDA reaction of tetrayne 1, subsequently reacts with the negative selenium ion at the acetylene carbon atoms with small steric hindrance via a nucleophilic addition reaction producing a P-Se four-membered ring intermediate B (Wang and Hoye, 2016; Gupta et al., 2018). Intermediate B is converted to intermediate C via a 4π-electrocyclic ring-opening reaction involving the breaking of P–Se bonds (Kashiki et al., 2009; Yoshioka et al., 2011). The active intermediate C has a resonance structure D, and D then undergoes a intramolecular nucleophilic addition reaction to form E. A migration of an aromatic C-H bond (Yamamoto and Takimiya, 2007) and elimination of HPPh_2_ from intermediate E affords the final product three. HPPh_2_ was oxidized to Ph_2_P(O)H. Fortunately, diphenylphosphine was isolated from the reaction process, and this by-product was characterized by nuclear magnetic resonance and gas chromatography/mass spectrometry.

## Conclusions

We provide here, to the best of our knowledge, the first method for synthesizing highly substituted dibenzoselenophenes and dibenzothiophenes via triphenylphosphine selenide or triphenylphosphine sulfide with multiynes. The formation of C_aryl_-S(Se)–C_aryl_ and C–C bonds contains benzyne cycloaddition and an intramolecular C–H migration. The reactions produce all of the highly substituted targets and exhibit excellent regioselectivity as well, with the rare selenophene and thiophene derivatives giving good yields under atmospheric conditions. This method offers new opportunities for the synthesis of complex benzothio[seleno]phenes and building blocks for functional materials. Specifically, this approach is an inexpensive alternative to oxidative couplings using cycloaddition/σ-bond migration of an aromatic C–H bond and subsequent elimination; the novel method provides better yields and can be applied in anti-cancer drug discovery, the dye industry, and cell biology. Future studies will be focused on the construction of natural thiophene cores and development of a more efficient pathway for producing ubiquitous thiophenes and selenophenes.

## Methods

### Procedure for Substituted Dibenzothio[Seleno]Phenes

Tetraynes (1.0 equiv) and triphenylphosphine sulfide (1.05 equiv) were added to toluene (1.5 mL), and the mixture was stirred at room temperature for 0.5 h and then heated at 105°C for 16 h under an air atmosphere. The reaction mixture was cooled to room temperature, and the solvent evaporated *in vacuo*. The residue was purified by preparative thin-layer chromatography on silica gel with the appropriate mixture of petroleum ether and ethyl acetate to give the fused multifunctionalized dibenzoselenophene or dibenzothiophene derivatives.

## Data Availability

The authors declare that all relevant data supporting the findings of this study are available within the article and Supplementary Information Files. The X-ray crystallographic coordinates for structures reported in this study have been deposited at the Cambridge Crystallographic Data Center (CCDC), under deposition numbers 1878394 (3j), 1878395 (3k), 1878393 (3m), 1878397 (3v), and 1878396 (3w). These data can be obtained free of charge from the CCDC via http://www.ccdc.cam.ac.uk/data_request/cif/.

## Author Contributions

BL, YW, BF, and XX worked on the presented work under the guidance of YH. The manuscript was written by BL and QH with inputs from all authors.

### Conflict of Interest Statement

The authors declare that the research was conducted in the absence of any commercial or financial relationships that could be construed as a potential conflict of interest.
