# Supplementary material for: Versatile Dibenzothio[seleno]phenes via Hexadehydro-Diels–Alder Domino Cyclization
Source: Front Chem. 2019 May 24;7:374. doi: 10.3389/fchem.2019.00374 (PMC6543197; doi:10.3389/fchem.2019.00374)

Supporting Information for

Versatile Dibenzothio[seleno]phenes via Hexadehydro-Diels–Alder Domino Cyclizations

**Contents**

1. General Experimental Procedures..………………………………….…..…..……………S2

2. Characterization Data for the New Compounds..………………………………….…..…S3

3. X-Ray Structure for **3j**, **3k**, **3m**, **3v** and **3w**….……………..……………………………S13

4. ^1^H and ^13^C NMR Spectra for the Compounds..………………………………………..…S14

**1. General experimental procedures**

All the catalytic reactions were performed under an argon atmosphere using the oven-dried Schlenk flask. The chemicals were purchased from Alfa Aesar and Acros Chemicals. All solvents and materials were pre-dried, redistilled or recrystallized before use. ^1^H NMR (300 MHz) and ^13^C NMR (125 MHz) spectra were recorded on a Bruker Avance 300 spectrometer with CDCl_3_ as the solvent. Chemical shifts are reported in ppm by assigning TMS resonance in the ^1^H NMR spectra as 0.00 ppm and CDCl_3_ resonance in the ^13^C spectra as 77.0 ppm. All coupling constants (*J* values) were reported in Hertz (Hz). Column chromatography was performed on silica gel 300–400 mesh. Melting points were determined using a Gallenkamp melting point apparatus and are uncorrected. The FT-IR spectra were recorded from KBr pellets or thin film from CHCl_3_ on the NaCl window in the 4000-400 cm^-1^ ranges on a Nicolet 5DX spectrometer. All HRMS spectra were record using EI at 70 eV. X-ray Crystallography diffraction data of **3j**, **3k**, **3m**, **3v**, and **3w** were collected at room temperature with a Bruker SMART Apex CCD diffractometer with Mo-K*α* radiation (*λ* = 0.71073 Å ) with a graphite monochromator using the *ω*-scan mode. Data reductions and absorption corrections were performed with SAINT and SADABS software, respectively. The structure was solved by direct methods and refined on *F^2^* by full-matrix least squares using SHELXTL. All non-hydrogen atoms were treated anisotropically. The positions of hydrogen atoms were generated geometrically.

**General procedures:**

Typical experimental procedure: Tetraynes (1.0 equiv), triphenylphosphine sulfide (1.05 equiv), were added to toluene (1.5 mL), the mixture was stirred at room temperature for half an hour and then heated at 110 °C for 16 hours under air atmosphere. The reaction mixture was cooled to room temperature, and the solvent was evaporated in vacuo. The residue was purified by preparative thin-layer chromatography (TLC) on silica gel with the appropriate mixture of petroleum ether and ethyl acetate to give the fused multifunctionalized dibenzoselenophene or dibenzothiophene derivatives.

1. **Characterization Data for the New Compounds**

**Diisopropyl 5-(4-fluorophenyl)-4-((4-fluorophenyl)ethynyl)-1H-benzo[b]indeno[5,4-d]selenophene**

**-2,2(3H)-dicarboxylate (3a)**

White solid; 532 mg (83 % yield); m. p. 183-185℃; TLC (petroleum ether/EtOAc = 10:1): Rf = 0.45; ^1^H NMR (300 MHz, CDCl_3_): δ 7.87 (d, *J* = 7.5 Hz, 1H; Ar-H), 7.40 (t, *J* = 7.5 Hz, 2H; Ar-H), 7.32-7.26 (m, 3H; Ar-H), 7.17-7.06 (m, 3H; Ar-H), 6.98 (t, *J* = 9.0 Hz, 2H; Ar-H), 6.85 (t, d = 9 Hz, 1H; Ar-H), 5.17-5.09 (m, 2H; CH(CH_3_)_2_), 3.92 (s, 2H;CH_2_), 3.79 (s, 2H; CH_2_), 1.32 (d, *J* = 6.0 Hz, 12H; CH(CH3)2); ^13^C NMR (125 MHz, CDCl_3_): 170.9, 163.5 (d, *J* = 18.8 Hz), 161.5 (d, *J* = 21.3 Hz), 140.3, 139.6 (d, *J* = 5 Hz), 138.3, 136.2, 136.1 (d, *J* = 3.8 Hz), 136.0, 135.4, 133.1 (d, *J* = 7.5 Hz), 131.5 (d, *J* = 7.5 Hz), 126.5, 126.3, 126.1, 124.5, 119.4, 117.9, 115.8 (d, *J* = 21.3 Hz), 115.6 (d, *J* = 22.5 Hz), 95.9, 86.6, 69.6, 59.8, 41.9, 41.3, 21.6 ppm; FT-IR (KBr): ν 3454, 2970, 2926, 2359, 2340, 1726, 1599, 1506, 1460, 1371, 1261, 1190, 1155, 1103, 837, 812, 752, 521, cm-1; HRMS (APCI): m/z calcd for C_37_H_30_F_2_O_4_Se [M+H]+, 657.1350; found 657.1344.

**Diisopropyl 5-(p-tolyl)-4-(p-tolylethynyl)-1H-benzo[b]indeno[5,4-d]selenophene-2,2(3H)-dicarboxylate (3b)**

White solid; 570 mg (79 % yield); m. p. 159-160℃; TLC (petroleum ether/EtOAc = 10:1): Rf = 0.53; ^1^H NMR (300 MHz, CDCl_3_): δ 7.85 (d, *J* = 9.0 Hz, 1H; Ar-H), 7.50-7.43 (m, 1H; Ar-H), 7.37 (d, *J* = 9.0 Hz, 1H; Ar-H), 7.30-7.20 (m, 3H; Ar-H), 7.13-7.05 (m, 4H; Ar-H); 6.91 (d, *J* = 9.0 Hz, 1H; Ar-H), 5.14-5.09 (m, 2H; CH(CH_3_)_2_), 3.85 (d, *J* = 45.0 Hz, 2H;CH_2_), 3.70 (d, *J* = 72.0 Hz, 2H; CH_2_), 2.53 (s, 3H; CH_3_), 2.32 (s, 3H; CH_3_), 1.32-1.25 (m,12H; CH(CH_3_)_2_); ^13^C NMR (125 MHz, CDCl_3_): δ 171.0, 143.8, 143.1, 141.6, 141.2, 139.6, 139.6, 138.6, 138.4, 138.0, 137.2, 137.2, 137.1, 136.9, 135.7, 135.5, 135.3, 132.9, 132.7, 131.3, 131.2, 129.5, 129.4, 129.1, 129.0, 128.9, 128.6, 127.4, 127.1, 126.8, 126.0, 125.9, 124.4, 120.5, 120.4, 118.2, 117.5, 96.8, 86.7, 69.5, 69.3, 59.7, 58.9, 41.8, 41.7, 41.4, 21.6, 21.5, 21.5, 21.2 ppm; FT-IR (KBr): ν 3454, 2978, 2360, 2340, 1724, 1508, 1452, 1263, 1190, 1103, 815, 739, cm-1; HRMS (APCI): m/z calcd for C_39_H_36_O_4_Se [M+H]+, 649.1852; found 649.1848.

**Diisopropyl 5-(4-chlorophenyl)-4-((4-chlorophenyl)ethynyl)-1H-benzo[b]indeno[5,4-d]selenophene-2,2(3H)-**

**dicarboxylate (3c)**

Light yellow solid; 552 mg (81 % yield); m. p. 178-180℃; TLC (petroleum ether/EtOAc = 10:1): Rf = 0.56; ^1^H NMR (300 MHz, CDCl_3_): δ 7.85 (d, *J* = 9.0 Hz, 1H; Ar-H), 7.55 (d, *J* = 6.0 Hz, 1H; Ar-H), 7.37-7.24 (m, 5H; Ar-H), 7.11-7.04 (m, 3H; Ar-H), 6.88 (d, *J* = 9.0 Hz, 1H; Ar-H); 5.15-5.07 (m, 2H; CH(CH_3_)_2_), 3.89 (s, 2H;CH_2_), 3.76 (d, 2H; CH_2_), 1.29 (d, *J* = 6.0 Hz, 12H; CH(CH_3_)_2_); ^13^C NMR (125 MHz, CDCl3): 170.9, 140.1, 139.7, 139.6, 138.6, 138.1, 136.4, 136.2, 135.2, 134.2, 133.9, 132.5, 131.3, 129.1, 128.6, 126.5, 126.4, 126.1, 124.6, 121.7, 117.6, 96.0, 87.8, 69.6, 59.7, 41.8, 41.3, 21.6 ppm; FT-IR (KBr): ν 3454, 2978, 2360, 2340, 1726, 1489, 1246, 1184, 1101, 825, 743, cm-1; HRMS (APCI): m/z calcd for C_37_H_30_Cl_2_O_4_Se [M+H]+, 689.0759; found 689.0758.

**Diisopropyl 5-(4-ethylphenyl)-4-((4-ethylphenyl)ethynyl)-1H-benzo[b]indeno[5,4-d]selenophene-2,2(3H)-dicarboxylate (3d)**

Light yellow solid; 524 mg (78 % yield); m. p. 168-169℃; TLC (petroleum ether/EtOAc = 10:1): Rf = 0.55; ^1^H NMR (300 MHz, CDCl_3_): δ 7.84 (d, *J* = 9.0 Hz, 1H; Ar-H), 7.35(dd, *J* = 9.0, 24.0 Hz, 4H; Ar-H), 7.05 (t, *J* = 7.5 Hz, 5H; Ar-H), 6.91 (d, *J* = 6.0 Hz, 1H; Ar-H); 5.15-5.07 (m, 2H; CH(CH_3_)_2_), 3.92 (s, 2H;CH_2_), 3.76 (d, 2H; CH_2_), 2.83 (q, *J* = 7.5 Hz, 2H;CH_2_CH_3_ ), 2.61 (q, *J* = 7.5 Hz, 3H; CH_2_CH_3_), 1.39 (t, *J* = 7.5 Hz, 3H; CH_2_CH_3_), 1.29 (d, *J* = 6.0 Hz, 12H; CH(CH_3_)_2_), 1.20 (t, *J* = 7.5 Hz, 2H; CH_2_CH_3_); ^13^C NMR (125 MHz, CDCl3): 171.0, 144.4, 143.8, 141.8, 139.5, 139.5, 138.6, 137.5, 135.6, 135.5, 135.3, 131.4, 129.6, 128.3, 127.7, 126.8, 126.0, 125.9, 124.4, 120.7, 118.3, 97.0, 86.8, 69.5, 59.8, 41.8, 41.4, 29.0, 28.8, 21.6, 16.1, 15.4 ppm; FT-IR (KBr): ν 3454, 2960, 2927, 1728, 1510, 1458, 1388, 1261, 1188, 1103, 1060, 833, 750, cm-1; HRMS (APCI): m/z calcd for C_41_H_40_O_4_Se [M+H]+, 677.2165; found 677.2168.

**Diisopropyl 5-(m-tolyl)-4-(m-tolylethynyl)-1H-benzo[b]indeno[5,4-d]selenophene-2,2(3H)-dicarboxylate (3e)**

Light yellow solid; 501 mg (78 % yield); m. p. 198-199℃; TLC (petroleum ether/EtOAc = 10:1): Rf = 0.53 ^1^H NMR (300 MHz, CDCl_3_): δ 7.84 (d, *J* = 6.0 Hz, 1H; Ar-H), 7.45 (t, *J* = 7.5 Hz, 1H; Ar-H), 7.35 (d, *J* = 9.0 Hz, 1H; Ar-H), 7.26-7.20 (m, 3H; Ar-H), 7.13 (t, *J* = 7.5 Hz, 1H; Ar-H), 7.04 (t, *J* = 9.0 Hz, 2H; Ar-H), 6.90 (t, *J* = 10.5 Hz, 3H; Ar-H), 5.15-5.07 (m, 2H; CH(CH_3_)_2_), 3.92 (s, 2H;CH_2_), 3.76 (s, 2H; CH_2_), 2.44 (s, 3H; CH_3_), 2.29 (s, 3H; CH_3_), 1.30 (d, *J* = 6.0 Hz, 12H; CH(CH_3_)_2_); ^13^C NMR (125 MHz, CDCl3): 171.0, 141.8, 140.0, 139.6, 139.5, 138.6, 138.3, 137.8, 135.9, 135.6, 135.2, 132.0, 130.3, 128.8, 128.7, 128.4, 128.3, 128.0, 126.9, 126.7, 126.1, 125.9, 124.4, 123.4, 117.9, 105.0, 97.0, 87.0, 69.5, 59.8, 41.9, 41.4, 21.6, 21.2 ppm; FT-IR (KBr): ν 3454, 2978, 2922, 1728, 1600, 1386, 1373, 1282, 1188, 1099, 1064, 746, 690, cm-1; HRMS (APCI): m/z calcd for C_39_H_36_O_4_Se [M+H]+, 649.1852; found 649.1850.

**Diisopropyl 5-phenyl-4-(phenylethynyl)-1H-benzo[b]indeno[5,4-d]selenophene-2,2(3H)-dicarboxylate (3f)**

White solid; 514 mg (80 % yield); m. p. 164-165℃; TLC (petroleum ether/EtOAc = 8:1): Rf = 0.51; ^1^H NMR (300 MHz, CDCl_3_): δ 7.84 (d, *J* = 9.0 Hz, 1H; Ar-H), 7.55 (s, 3H; Ar-H), 7.42 (t, *J* = 4.5 Hz, 1H; Ar-H), 7.26-7.23 (m, 4H; Ar-H), 7.12- 7.00 (m, 3H; Ar-H), 6.83 (d, *J* = 9.0 Hz, 1H; Ar-H), 5.16-5.08 (m, 2H; CH(CH_3_)_2_), 3.93 (s, 2H;CH_2_), 3.77 (s, 2H; CH_2_), 1.30 (d, *J* = 6.0 Hz, 12H; CH(CH_3_)_2_); ^13^C NMR (125 MHz, CDCl3): 171.0, 141.6, 140.2, 139.6, 139.5, 138.4, 136.0, 135.7, 135.3, 131.4, 129.7, 128.8, 128.1, 128.0, 127.8, 126.8, 126.1, 126.0, 124.5, 123.5, 117.9, 96.8, 87.2, 69.5, 59.8, 41.9, 41.4, 21.6 ppm; FT-IR (KBr): ν 3454, 2982, 2361, 1726, 1489, 1458, 1369, 1259, 1190, 1103, 753, 687, 529 cm-1; HRMS (APCI): m/z calcd for C_37_H_32_O_4_Se [M+H]+, 621.1539; found 621.1535.

**Dimethyl 5-(4-fluorophenyl)-4-((4-fluorophenyl)ethynyl)-1H-benzo[b]indeno[5,4-d]selenophene-2,2(3H)-dicarboxylate (3g)**

White solid; 493 mg (82 % yield); m. p. 209-210℃; TLC (petroleum ether/EtOAc = 8:1): Rf = 0.28; ^1^H NMR (300 MHz, CDCl_3_): δ 7.85 (d, *J* = 9.0 Hz, 1H; Ar-H), 7.38 (t, *J* = 7.5 Hz, 2H; Ar-H), 7.30-7.22 (m, 3H; Ar-H), 7.15-7.04 (m, 3H; Ar-H), 6.96 (t, *J* = 7.5 Hz, 2H; Ar-H), 6.83 (d, *J* = 9.0 Hz, 1H; Ar-H), 3.95 (s, 2H; CH_2_), 3.83 (s, 2H; CH_2_), 3.83 (s, 6H; OCH3); ^13^C NMR (125 MHz, CDCl_3_): δ 171.8, 163.5 (d, *J* = 17.5 Hz), 161.5 (d, *J* = 20.0 Hz), 140.3, 139.6, 139.4, 138.2, 136.2, 136.0(d, *J* = 3.8 Hz) , 135.8, 135.5, 133.1 (d, *J* = 8.8 Hz), 131.5(d, *J* = 8.8 Hz) , 131.0 (d, *J* = 8.8 Hz), 130.9, 126.5, 126.4, 126.1, 124.6, 119.3, 117.9, 115.9(d, *J* = 20 Hz) , 115.6 (d, *J* = 21.3 Hz), 96.0, 86.5, 59.6, 53.3, 53.2, 53.1, 42.0, 41.6 ppm; FT-IR (KBr): ν 3479, 2954, 2360, 2341, 1734, 1251, 1215, 1153, 1070, 835, 746, 518 cm-1; HRMS (APCI): m/z calcd for C_33_H_22_F_2_O_4_Se [M+H]+, 601.0724; found 601.0716.

**Dimethyl 5-(p-tolyl)-4-(p-tolylethynyl)-1H-benzo[b]indeno[5,4-d]selenophene-2,2(3H)-dicarboxylate (3h)**

White solid; 462 mg (78 % yield); m. p. 166-167℃; TLC (petroleum ether/EtOAc = 8:1): Rf = 0.33; ^1^H NMR (300 MHz, CDCl_3_): δ 7.85 (d, *J* = 9.0 Hz, 1H; Ar-H), 7.38 (d, *J* = 9.0 Hz, 2H; Ar-H), 7.31-7.23 (m, 3H; Ar-H), 7.09-7.05 (m, 5H; Ar-H), 6.93 (t, *J* = 9 Hz, 1H; Ar-H), 3.97 (s, 2H; CH_2_), 3.83(s, 2H; CH_2_), 3.83 (s, 6H; OCH3), 2.54(s, 3H; CH_3_), 2.33(s, 3H; CH_3_); ^13^C NMR (125 MHz, CDCl_3_): δ 171.9, 141.7, 139.5, 139.3, 138.6, 138.1, 137.3, 137.1, 135.7, 135.4, 135.2, 131.2, 129.5, 129.4, 128.9, 126.8, 126.1, 125.9, 124.4, 120.4, 118.2, 97.0, 86.6, 59.6, 53.3, 42.0, 41.6, 21.5 ppm; FT-IR (KBr): 3479, 2358, 1732, 1487, 1436, 1301, 1267,1195,1168,1089,1068, 827, 754,cm-1 HRMS (APCI): m/z calcd for C_35_H_28_O_4_Se [M+H]+, 593.1226; found 593.1233.

**Dimethyl 5-(4-chlorophenyl)-4-((4-chlorophenyl)ethynyl)-1H-benzo[b]indeno[5,4-d]selenophene-2,2(3H)-dicarboxylate (3i)**

White solid; 518 mg (82 % yield); m. p. 207-208℃; TLC (petroleum ether/EtOAc = 8:1): Rf = 0.35; ^1^H NMR (300 MHz, CDCl_3_): δ 7.86 (d, *J* = 6.0 Hz, 1H; Ar-H), 7.56 (d, *J* = 9.0 Hz, 2H; Ar-H), 7.36 (d, *J* = 9.0 Hz, 2H; Ar-H), 7.30-7.24 (m, 3H; Ar-H), 7.12-7.04 (m, 3H; Ar-H), 6.88 (d, *J* = 9.0 Hz, 1H; Ar-H), 3.94 (s, 2H; CH_2_), 3.83(s, 2H; CH_2_), 3.83 (s, 6H; OCH3); ^13^C NMR (125 MHz, CDCl_3_): δ 171.8, 140.1, 139.6, 139.4, 138.6, 138.1, 136.4, 136.0, 135.3, 134.2, 133.9, 132.5, 131.2, 129.1, 128.7, 126.5, 126.5, 126.1, 124.7, 121.6, 117.6, 105.0, 96.1, 87.7, 59.6, 53.3, 42.0, 41.5 ppm; FT-IR (KBr): ν 3479, 2951, 2360, 1735, 1491, 1259, 1157, 1087，825, 744, 667 cm-1；HRMS (APCI): m/z calcd for C_33_H_22_Cl_2_O_4_Se [M+H]+, 633.0133; found 633.0130.

**Dimethyl 5-(4-ethylphenyl)-4-((4-ethylphenyl)ethynyl)-1H-benzo[b]indeno[5,4-d]selenophene-2,2(3H)-dicarboxylate (3j)**

White solid; 492 mg (79 % yield); m. p. 192-194℃; TLC (petroleum ether/EtOAc = 8:1): Rf = 0.37; ^1^H NMR (300 MHz, CDCl_3_): δ 7.84 (d, *J* = 6.0 Hz, 1H; Ar-H), 7.39 (d, *J* = 6.0 Hz, 2H; Ar-H), 7.32-7.22 (m, 3H; Ar-H), 7.04 (dd, *J* = 9.0, 15.0 Hz, 5H; Ar-H), 6.92 (d, *J* = 9.0 Hz, 1H; Ar-H), 3.97 (s, 2H; CH_2_), 3.82(s, 2H; CH_2_), 3.82 (s, 6H; OCH_3_), 2.84(q, *J* = 7.5 Hz, 2H; CH_2_CH_3_), 2.61(q, *J* = 7.5 Hz, 2H; CH_2_CH_3_), 1.39 (t, *J* = 7.5 Hz, 3H; CH_2_CH_3_), 1.20 (t, *J* = 7.5 Hz, 3H; CH_2_CH_3_); ^13^C NMR (125 MHz, CDCl_3_): δ 172.0, 144.5, 143.8, 141.9, 139.5, 139.2, 138.6, 137.4, 135.6, 135.5, 135.3, 131.4, 129.6, 128.3, 127.7, 127.4, 126.9, 126.1, 125.9, 124.5, 120.6, 118.3, 97.1, 86.7, 59.7, 53.2, 42.0, 41.6, 29.0, 28.8, 16.1, 15.4 ppm; FT-IR (KBr): ν 3479, 2960, 1732, 1508, 1429, 1278, 1249, 1197, 1155, 1047, 829，748, cm-1; HRMS (APCI): m/z calcd for C_37_H_32_O_4_Se [M+H]+, 621.1539; found 621.1540.

**Dimethyl 5-phenyl-4-(phenylethynyl)-1H-benzo[b]indeno[5,4-d]selenophene-2,2(3H)-dicarboxylate (3k)**

White solid; 489 mg (80 % yield); m. p. 202-203℃; TLC (petroleum ether/EtOAc = 8:1): Rf = 0.39; ^1^H NMR (300 MHz, CDCl_3_): δ 7.86 (d, *J* = 6.0 Hz, 1H; Ar-H), 7.59-7.57 (m, 3H; Ar-H), 7.44-7.42 (m, 2H; Ar-H), 7.32-7.25 (m, 4H; Ar-H), 7.14-7.12 (m, 2H; Ar-H), 7.04 (t, *J* = 7.5 Hz, 1H; Ar-H), 6.85 (d, *J* = 9.0 Hz, 1H; Ar-H), 4.00 (s, 2H; CH_2_), 3.84 (s, 2H; CH_2_), 3.84 (s, 6H; OCH_3_); ^13^C NMR (125 MHz, CDCl_3_): δ 172.3, 142.1, 140.5, 134.0, 139.8, 138.8, 136.4, 135.9, 135.8, 131.7, 130.0, 129.2, 128.6, 128.4, 128.2, 127.1, 126.6, 126.4, 124.9, 123.7, 118.3, 97.3, 87.5, 60.0, 53.6, 42.4, 42.0 ppm; FT-IR (KBr): ν 3479, 2953, 2360, 2339, 1735, 1440, 1274, 1244, 1157, 1072, , 744, 704 cm-1; HRMS (APCI): m/z calcd for C_33_H_24_O_4_Se [M+H]+, 565.0913; found 565.0912.

**Diethyl 5-(4-propylphenyl)-4-((4-propylphenyl)ethynyl)-1H-benzo[b]indeno[5,4-d]selenophene-2,2(3H)-dicarboxylate (3l)**

White solid; 480 mg (72% yield); m. p. 179-181℃; TLC (petroleum ether/EtOAc = 10:1): R_f_ = 0.43; ^1^H NMR (300 MHz, CDCl_3_): *δ* 7.83 (d, *J* = 9.0 Hz; 1H, Ar-H), 7.33 (dd, *J* = 7.8, 19.5 Hz; 4H; Ar-H), 7.22 (d, *J* = 9.0 Hz; 1H; Ar-H), 7.12-7.00 (m, 5H; Ar-H), 6.86 (d, 1H, *J* = 6.0 Hz; Ar-H), 4.28 (q, 4H, *J* = 6.9 Hz; OCH_2_CH_3_), 3.96 (s, 2H; CH_2_), 3.80 (s, 2H; CH_2_), 2.77 (t, 2H, *J* = 7.2 Hz; CH_2_CH_2_CH_3_), 2.54 (t, 2H, *J* = 7.2 Hz; CH_2_CH_2_CH_3_), 1.80 (q, 2H, *J* = 7.2 Hz; CH_2_CH_2_CH_3_), 1.60 (q, 2H, *J* = 7.5 Hz; CH_2_CH_2_CH_3_),1.31 (t, *J* = 6.9 Hz; 6H; CH_2_CH_3_), 1.04 (t, *J* = 7.2 Hz; 3H; CH_2_CH_2_CH_3_), 0.91 (t, *J* = 7.2 Hz; 3H; CH_2_CH_2_CH_3_); ^13^C NMR (125 MHz, CDCl_3_): δ 171.5, 142.9, 142.1, 141.8, 139.5, 139.4, 138.6, 137.4, 135.6, 135.4, 135.4, 131.3, 129.5, 128.9, 128.3, 126.8, 126.1, 125.9, 124.4, 120.7, 118.2, 97.0, 86.7, 62.0, 59.7, 41.9, 41.5, 38.0, 24.8, 24.4, 14.1, 13.7 ppm; FT-IR (KBr): *ν* 3446, 2960, 2927, 2360, 2240，1728, 1510, 1458, 1259, 1182, 1161, 1089, 1068, 750 cm^-1^; HRMS (APCI): *m/z* calcd for C_41_H_40_O_4_Se [M + H]^+^,677.2165; found: 677.2171.

**Diethyl 5-(4-fluorophenyl)-4-((4-fluorophenyl)ethynyl)-1H-benzo[b]indeno[5,4-d]selenophene-2,2(3H)-dicarboxylate (3m)**

Light yellow solid; 516 mg (82 % yield); m. p. 173-175℃; TLC (petroleum ether/EtOAc = 10:1): *R*_f_ = 0.41; ^1^H NMR (300 MHz, CDCl_3_): *δ* 7.87 (d, *J* = 7.8 Hz, 1H; Ar-H), 7.39 (dd, *J* = 5.7, 7.8 Hz, 2H; Ar-H), 7.31-7.26 (m, 3H; Ar-H), 7.16-7.05 (m, 3H;Ar-H), 6.97 (t, *J* = 7.5 Hz, 2H; Ar-H), 6.84 (d, *J* = 6.0 Hz, 1H; Ar-H), 4.29 (q, *J* =7.2 Hz, 4H; OCH_2_CH_3_), 3.95 (s, 2H; CH_2_), 3.82 (s, 2H; CH_2_), 1.32 (t, *J* = 7.2 Hz, 6H; OCH_2_CH_3_); ^13^C NMR (125 MHz, CDCl_3_): 171.4, 163.5(d, *J* = 17.5 Hz), 161.5(d, *J* = 20.0 Hz), 140.3, 139.6, 139.5, 138.2, 136.1(d, *J* = 2.5 Hz), 135.9, 135.4, 133.2, 133.1 (d, *J* = 8.8 Hz), 131.5 (d, *J* = 7.5 Hz), 126.5, 126.3, 126.1, 124.6, 119.3(d, *J* = 2.5 Hz), 117.9, 115.8 (d, *J* = 21.3 Hz), 115.6 (d, *J* = 22.5 Hz), 95.9, 86.6, 62.1, 59.7, 41.9, 41.4, 14.1 ppm; FT-IR (KBr): *ν* 3446, 2980, 2360, 1726, 1598, 1506, 1259, 1234, 1186, 1155, 1089, 1066, 837, cm^-1^; HRMS (APCI): *m/z* calcd for C_35_H_26_F_2_O_4_Se [M+H]^+^, 629.1037; found 629.1035.

**Diethyl 5-(p-tolyl)-4-(p-tolylethynyl)-1H-benzo[b]indeno[5,4-d]selenophene-2,2(3H)-dicarboxylate (3n)**

White solid; 464 mg (75 % yield); m. p. 151-152℃; TLC (petroleum ether/EtOAc = 10:1): *R*_f_ = 0.42; ^1^H NMR (300 MHz, CDCl_3_): *δ* 7.84 (d, *J* = 9.0 Hz, 1H; Ar-H), 7.49-7.42 (m, 1H; Ar-H), 7.36 (d, *J* = 9.0 Hz, 1H; Ar-H), 7.29-7.19 (m, 5H;Ar-H), 6.12-7.03 (m, 4H; Ar-H), 6.91 (d, *J* = 6.0 Hz, 1H; Ar-H), 4.28 (q, *J* =6.0 Hz, 4H; OCH_2_CH_3_), 3.95 (s, 2H; CH_2_), 3.80 (s, 2H; CH_2_), 2.53 (s, 3H; CH_3_), 2.32 (s, 3H; CH_3_), 1.31 (t, *J* = 6.0 Hz, 6H; OCH_2_CH_3_); ^13^C NMR (125 MHz, CDCl_3_):δ 171.5, 141.7, 139.5, 139.5, 138.6, 138.1, 137.3, 137.1, 135.7, 133.0, 132.7, 131.3, 131.3, 129.5, 129.4, 129.1, 128.9, 128.6, 128.2, 127.4, 126.8, 126.1, 125.9, 124.4, 120.5, 118.3, 117.0, 116.9, 96.9, 86.7, 62.0, 61.9, 59.8, 41.9, 41.5, 21.5, 14.1, 14.0 ppm; FT-IR (KBr): *ν* 3446, 2978, 2920, 2360, 2340, 1726, 1508, 1458, 1261, 1226, 1184, 1068, 815, 732, cm^-1^; HRMS (APCI): *m/z* calcd for C_37_H_32_O_4_Se [M+H]^+^, 621.1539; found 621.1542.

**Diethyl 5-(4-chlorophenyl)-4-((4-chlorophenyl)ethynyl)-1H-benzo[b]indeno[5,4-d]selenophene-2,2(3H)-dicarboxylate (3o)**

Light yellow solid; 536mg (81 % yield); m. p. 188-189℃; TLC (petroleum ether/EtOAc = 10:1): *R*_f_ = 0.45; ^1^H NMR (300 MHz, CDCl_3_): *δ* 7.85 (d, *J* = 9.0 Hz, 1H; Ar-H), 7.55 (d, *J* = 9.0 Hz, 2H; Ar-H), 7.35 (d, *J* = 9.0 Hz, 2H; Ar-H), 7.28-7.23 (m, 3H;Ar-H), 7.17-7.04 (m, 3H;Ar-H),6.89 (d, *J* = 9.0 Hz, 1H; Ar-H), 4.28 (q, *J* =7.0 Hz, 4H; OCH_2_CH_3_), 3.93 (s, 2H; CH_2_), 3.80 (s, 2H; CH_2_), 1.31 (t, *J* = 7.0 Hz, 6H; OCH_2_CH_3_); ^13^C NMR (125 MHz, CDCl_3_): δ 171.4, 140.1, 139.6, 138.6, 138.1, 136.4, 136.1, 135.2, 134.2, 133.9, 132.6, 132.5, 131.3, 130.6, 129.1, 128.7, 128.6, 128.1, 126.5, 126.4, 126.1, 124.6, 121.6, 117.6, 96.0, 87.8, 62.1, 59.7, 41.9, 41.4, 14.1 ppm; FT-IR (KBr): *ν* 3446, 2980, 2360, 2440，1726, 1489, 1261, 1186, 1166, 1089, 1014，827，cm^-1^; HRMS (APCI): *m/z* calcd for C_35_H_26_Cl_2_O_4_Se [M+H]^+^, 661.0446; found 661.0444.

**Diethyl 5-(4-ethylphenyl)-4-((4-ethylphenyl)ethynyl)-1H-benzo[b]indeno[5,4-d]selenophene-2,2(3H)-dicarboxylate (3p)**

White solid; 482mg (74% yield); m. p. 165-166℃; TLC (petroleum ether/EtOAc = 10:1): R_f_ = 0.43; ^1^H NMR (300 MHz, CDCl_3_): *δ* 7.84 (d, *J* = 9.0 Hz, 1H, ; Ar-H), 7.40-7.26 (m, 5H; Ar-H), 7.04-7.03 (d, *J* = 3.0 Hz, 5H; Ar-H), 6.91 (d, *J* = 9.0 Hz, 1H, Ar-H), 4.28 (q, *J* = 6.0 Hz, 4H, OCH_2_CH_3_), 3.95 (s, 2H; CH_2_), 3.80 (s, 2H; CH_2_), 2.83 (q, *J* = 7.3 Hz, 4H, CH_2_CH_3_), 2.62 (q, *J* = 7.3 Hz, 4H, CH_2_CH_3_), 1.39 (t, *J* = 7.3 Hz, 3H; CH_2_CH_3_), 1.31 (t, *J* = 6.0 Hz, 6H; OCH_2_CH_3_), 1.39 (t, *J* = 6.0 Hz, 3H; CH_2_CH_3_); ^13^C NMR (125 MHz, CDCl_3_): δ 170.5, 143.4, 142.8, 140.8, 138.5, 138.3, 137.6, 136.4, 134.6, 134.4, 130.3, 128.6, 128.3, 127.2, 126.8, 126.7, 126.3, 125.8, 125.1, 124.9, 123.4, 119.7, 117.3, 96.0, 85.7, 61.0, 58.7, 40.9, 40.4, 27.9, 27.8, 15.1, 14.4, 13.1 ppm; FT-IR (KBr): *ν* 3446,3047, 2962, 2927, 2361, 1724, 1510, 1458, 1261, 1186, 1068, 1018, 827, 750 cm^-1^; HRMS (APCI): *m/z* calcd for C_39_H_36_O_4_Se [M + H]^+^, 649.1852; found: 649.1845.

**Diethyl 5-phenyl-4-(phenylethynyl)-1H-benzo[b]indeno[5,4-d]selenophene-2,2(3H)-dicarboxylate (3q)**

White solid; 480 mg (80% yield); m. p. 172-173℃; TLC (petroleum ether/EtOAc = 10:1): R_f_ = 0.40; ^1^H NMR (300 MHz, CDCl_3_): *δ* 7.85 (d, 1H, *J* = 9.0 Hz; Ar-H), 7.55 (s, 3H; Ar-H), 7.42 (t, *J* = 3.0 Hz, 2H; Ar-H), 7.26-7.11 (m, 6H, Ar-H) 7.02 (t, *J* = 7.5 Hz, 1H; Ar-H), 6.84 (d, *J* = 9.0 Hz, 1H, Ar-H), 4.29 (q, *J* = 7.5 Hz, 4H, ; OCH_2_CH_3_), 3.97 (s, 2H; CH_2_), 3.81 (s, 2H; CH_2_), 1.32 (t, *J* = 7.5 Hz, 6H; CH_2_CH_3_); ^13^C NMR (125 MHz, CDCl_3_): δ 171.5, 141.7, 140.2, 139.6, 138.4, 136.0, 135.6, 135.3, 131.3, 129.7, 128.8, 128.2, 128.0, 127.8, 126.7, 126.2, 126.0, 124.5, 123.4, 117.9, 96.9, 87.1, 62.1, 59.7, 41.9, 41.5, 14.1 ppm; FT-IR (KBr): *ν* 3446,2978, 2360, 1726, 1441, 1259, 1186, 1068, 1018, 754, 705, 689, cm^-1^; HRMS (APCI): *m/z* calcd for C_35_H_28_O_4_Se [M + H]^+^, 593.1226; found: 593.1227.

**Diisopropyl 5-(p-tolyl)-4-(p-tolylethynyl)-1H-benzo[b]indeno[5,4-d]thiophene-2,2(3H)-dicarboxylate (3r)**

Light yellow solid;; 512 mg (81 % yield); m. p. 141-143℃; TLC (petroleum ether/EtOAc = 10:1): Rf = 0.51; 1H NMR (300 MHz, CDCl_3_): δ 7.81 (d, *J* = 9.0 Hz, 1H; Ar-H), 7.35 (t, *J* = 9.0 Hz, 5H; Ar-H), 7.12-7.05 (m, 5H; Ar-H), 6.93 (d, *J* = 9.0 Hz, 1H; Ar-H), 5.15-5.07 (m, 2H; CH(CH_3_)_2_), 3.93 (s, 2H;CH_2_), 3.82 (s, 2H; CH_2_), 2.53 (s, 3H; CH_3_), 2.32 (s, 3H; CH_3_), 1.30 (d, *J* = 6.0 Hz, 12H; CH(CH_3_)_2_); ^13^C NMR (125 MHz, CDCl3): 171.1, 140.5, 140.2, 139.5, 138.0, 137.3, 136.7, 135.9, 135.1, 132.9, 132.4, 131.3, 129.5, 129.3, 129.2, 129.0, 128.9, 128.6, 126.00, 124.9, 124.1, 122.7, 120.5, 117.3, 96.6, 86.6, 69.5, 59.9, 41.4, 40.5, 21.6, 21.5 ppm; FT-IR (KBr): ν 3454, 2980, 2918, 2360, 1726, 1508, 1462, 1419, 1371, 1261, 1190, 1103, 815, 752, cm-1; HRMS (APCI): m/z calcd for C_39_H_36_O_4_S [M+H]+, 601.2094; found 601.2092.

**Dimethyl 5-(p-tolyl)-4-(p-tolylethynyl)-1H-benzo[b]indeno[5,4-d]thiophene-2,2(3H)-dicarboxylate (3s)**

Light yellow solid; 493 mg (83 % yield); m. p. 149-150℃; TLC (petroleum ether/EtOAc = 8:1): Rf = 0.35; ^1^H NMR (300 MHz, CDCl_3_): δ 7.81 (d, *J* = 9.0 Hz, 1H; Ar-H), 7.34 (t, *J* = 7.5 Hz, 2H; Ar-H), 7.30-7.22 (m, 3H; Ar-H), 7.15-7.06 (m, 5H; Ar-H), 6.96 (t, *J* = 9 Hz, 1H; Ar-H), 3.98 (s, 2H; CH_2_), 3.88(s, 2H; CH_2_), 3.82 (s, 6H; OCH3), 2.53(s, 3H; CH_3_), 2.32(s, 3H; CH_3_); ^13^C NMR (125 MHz, CDCl_3_): δ 172.0, 140.6, 139.9, 139.5, 138.1, 137.4, 136.6, 135.8, 135.1, 133.0, 132.1, 131, 129.5, 129.4, 128.9, 126.1, 125.0, 124.1, 122.7, 120.4, 117.3, 96.7, 86.5, 59.8, 53.2, 41.6, 40.6, 21.5 ppm; FT-IR (KBr): ν 3479, 2953, 2358, 2339, 1728, 1508, 1436, 1263, 1195, 1166, 1095, 1070, 815, 785, 754, 513 cm-1; HRMS (APCI): m/z calcd for C_35_H_28_O_4_S [M+H]+, 545.1781; found 545.1788.

**Dimethyl 5-(4-chlorophenyl)-4-((4-chlorophenyl)ethynyl)-1H-benzo[b]indeno[5,4-d]thiophene-2,2(3H)-dicarboxylate (3t)**

Light yellow solid; 517 mg (81 % yield); m. p. 177-178℃; TLC (petroleum ether/EtOAc = 8:1): Rf = 0.34; ^1^H NMR (300 MHz, CDCl_3_): δ 7.85 (d, *J* = 9.0 Hz, 1H; Ar-H), 7.59 (d, *J* = 9.0 Hz, 2H; Ar-H), 7.42-7.26 (m, 4H; Ar-H), 7.16-7.09 (m, 3H; Ar-H), 6.94 (d, *J* = 6.0 Hz, 1H; Ar-H), 3.98 (s, 2H; CH_2_), 3.90(s, 2H; CH_2_), 3.85 (s, 6H; OCH3); ^13^C NMR (125 MHz, CDCl_3_): δ 171.8, 140.0, 139.6, 139.1, 138.1, 135.8, 135.4, 134.3, 134.0, 132.9, 132.8, 132.5, 131.3, 129.0, 128.7, 126.4, 124.7, 124.4, 122.9, 121.6, 116.7, 95.8, 87.7, 59.8, 53.3, 41.5, 40.6 ppm; FT-IR (KBr): ν 3479, 2953, 1726, 1489, 1431, 1251, 1157, 1085, 829, 740, cm-1; HRMS (APCI): m/z calcd for C_33_H_22_Cl_2_O_4_S [M+H]+, 585.0689; found 585.0685.

**Dimethyl 5-(4-ethylphenyl)-4-((4-ethylphenyl)ethynyl)-1H-benzo[b]indeno[5,4-d]thiophene-2,2(3H)-dicarboxylate (3u)**

Light yellow solid; 486 mg (79 % yield); m. p. 150-152℃; TLC (petroleum ether/EtOAc = 8:1): Rf = 0.42; ^1^H NMR (300 MHz, CDCl_3_): δ 7.81 (d, *J* = 9.0 Hz, 1H; Ar-H), 7.41-7.29 (m, 5H; Ar-H), 7.05 (s, 5H; Ar-H), 6.95 (d, *J* = 6.0 Hz, 1H; Ar-H), 3.98 (s, 2H; CH_2_), 3.88(s, 2H; CH_2_), 3.82 (s, 6H; OCH3), 2.84(q, *J* = 7.5 Hz, 2H; CH_2_CH_3_), 2.61(q, *J* = 7.5 Hz, 2H; CH_2_CH_3_), 1.40 (t, *J* = 7.5 Hz, 3H; CH_2_CH_3_), 1.21 (t, *J* = 7.5 Hz, 3H; CH_2_CH_3_); ^13^C NMR (125 MHz, CDCl_3_): δ 172.0, 144.5, 143.9, 140.7, 139.7, 139.5, 136.9, 135.8, 135.1, 133.0, 132.2, 131.4, 129.6, 128.2, 127.7, 126.1, 125.0, 124.1, 122.7, 120.6, 117.4, 96.8, 86.6, 77.3, 77.0, 76.8, 59.8, 53.2, 41.5, 40.6, 28.9, 28.8, 16.0, 15.4 ppm; FT-IR (KBr): ν 3479, 2962, 2360, 2342, 1734, 1429, 1280, 1257, 1197, 1157, 1093, 829, 800, 748 cm-1; HRMS (APCI): m/z calcd for C_37_H_32_O_4_S [M+H]+, 573.2094; found 573.2094.

**Dimethyl 5-phenyl-4-(phenylethynyl)-1H-benzo[b]indeno[5,4-d]thiophene-2,2(3H)-dicarboxylate (3v)**

White solid; 493 mg (80 % yield); m. p. 183-184℃; TLC (petroleum ether/EtOAc = 8:1): Rf = 0.38; ^1^H NMR (300 MHz, CDCl_3_): δ 7.82 (d, *J* = 9.0 Hz, 1H; Ar-H), 7.56 (s, 3H; Ar-H), 7.45 (t, *J* = 3.0 Hz, 2H; Ar-H), 7.33 (t, *J* = 7.5 Hz, 1H; Ar-H), 7.26-7.13 (m, 4H; Ar-H), 7.06 (t, *J* = 9.0 Hz, 1H; Ar-H), 6.87 (d, *J* = 9.0 Hz, 1H; Ar-H), 4.00 (s, 2H; CH_2_), 3.89 (s, 2H; CH_2_), 3.83 (s, 6H; OCH_3_); ^13^C NMR (125 MHz, CDCl_3_): δ 172.0, 140.6, 139.9, 139.7, 139.6, 135.7, 135.4, 132.9, 132.4, 131.4, 129.7, 128.74, 128.2, 128.0, 127.9, 126.2, 124.9, 124.2, 123.4, 122.8, 117.0, 96.7, 87.0, 59.8, 53.3, 41.6, 40.7 ppm; FT-IR (KBr): ν 3479, 3057, 2953, 2360, 2341, 1759, 1735, 1489, 1440, 1274, 1244, 1155, 1072, 746, 706, cm-1; HRMS (APCI): m/z calcd for C_33_H_24_O_4_S [M+H]+,517.1468; found 517.1464.

**Dimethyl 5-(4-fluorophenyl)-4-((4-fluorophenyl)ethynyl)-1H-benzo[b]indeno[5,4-d]thiophene-2,2(3H)-dicarboxylate (3w)**

White solid; 512 mg (82 % yield); m. p. 196-197℃; TLC (petroleum ether/EtOAc = 8:1): Rf = 0.34; ^1^H NMR (300 MHz, CDCl_3_): δ 7.83 (d, *J* = 9.0 Hz, 1H; Ar-H), 7.44-7.38 (m, 3H; Ar-H), 7.35-7.26 (m, 2H; Ar-H), 7.17-7.07 (m, 3H; Ar-H), 6.97 (t, *J* = 9.0 Hz, 2H; Ar-H), 6.87 (d, *J* = 9.0 Hz, 1H; Ar-H), 3.97 (s, 2H; CH_2_), 3.88 (s, 2H; CH_2_), 3.83 (s, 6H; OCH_3_); ^13^C NMR (125 MHz, CDCl_3_): δ 171.9, 163.5(d, *J* = 21.3 Hz), 161.5(d, *J* = 23.8 Hz), 139.9, 139.6, 139.2, 135.5, 135.5, 133.2 (d, *J* = 8.8 Hz), 133.0, 132.7, 131.5 (d, *J* = 8.8 Hz), 126.3, 124.6, 124.3, 122.9, 119.3, 117.0, 115.7 (t, *J* = 21.3 Hz), 95.7, 86.4, 59.8, 53.3, 53.3, 41.5, 40.6 ppm; FT-IR (KBr): ν 3479, 2947, 2360, 2339, 1739, 1600, 1506, 1435, 1249, 1232, 1155, 1074, 835, 748, 522,cm-1; HRMS (APCI): m/z calcd for C_33_H_22_F_2_O_4_S [M+H]+, 553.1280; found 553.1287.

**3. X-Ray Structure for 3j, 3k, 3m, 3v and 3w**


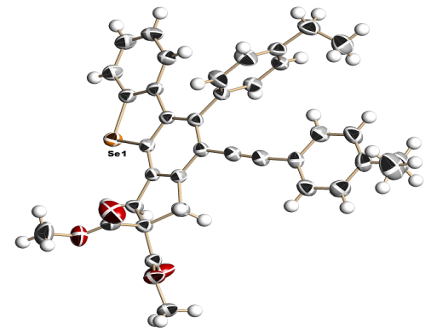


**3j**


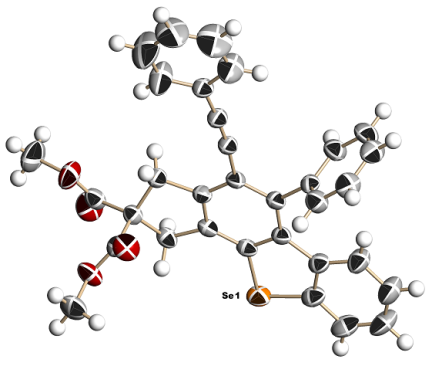


**3k**


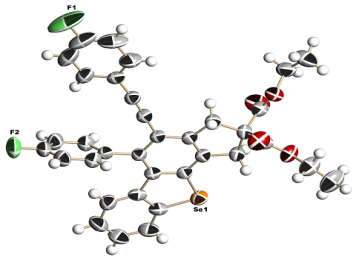


**3m**


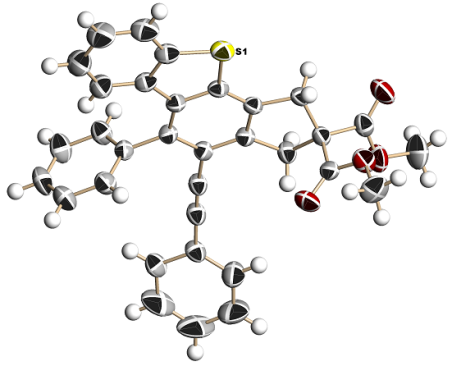


**3v**


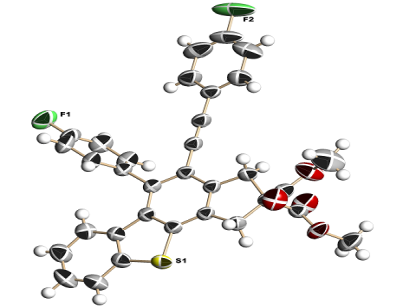


**3w**

**4. ^1^H NMR & ^13^C NMR Spectra for New Compounds**


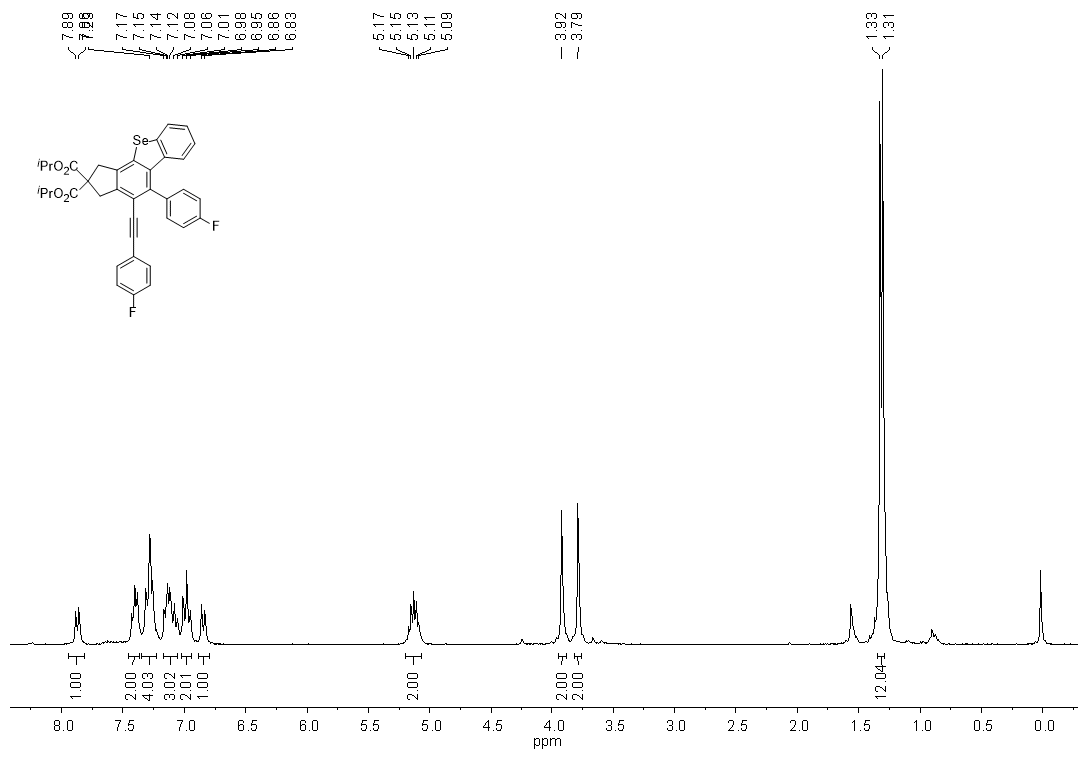


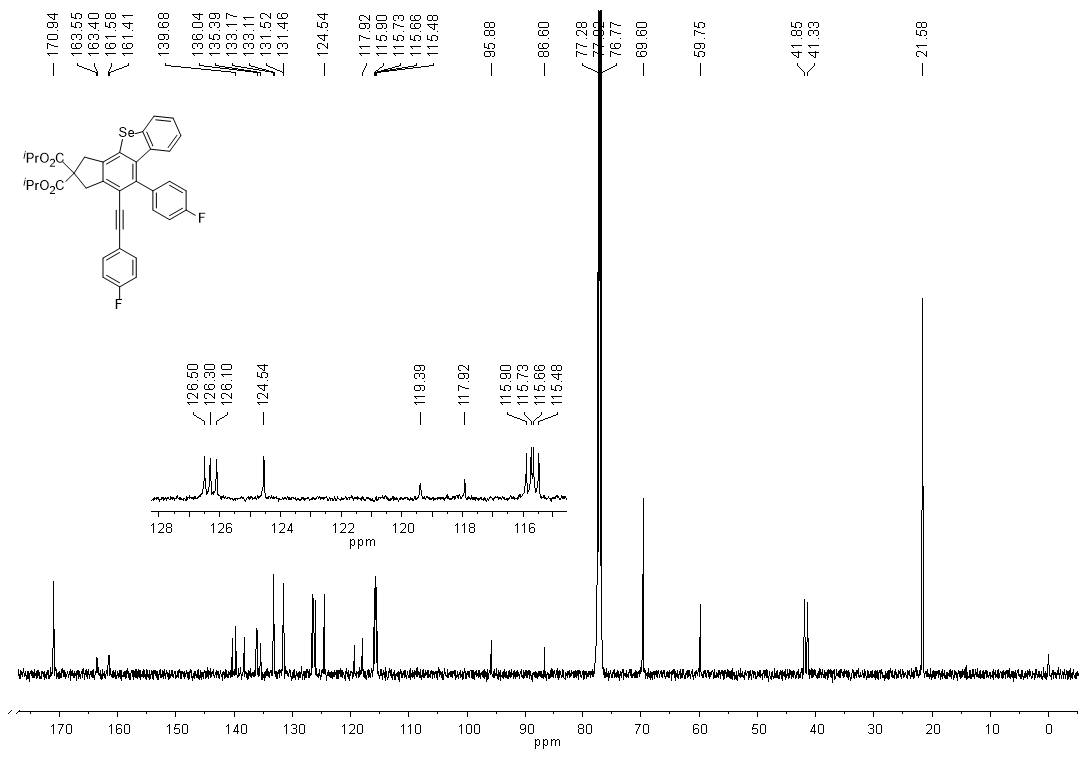


**
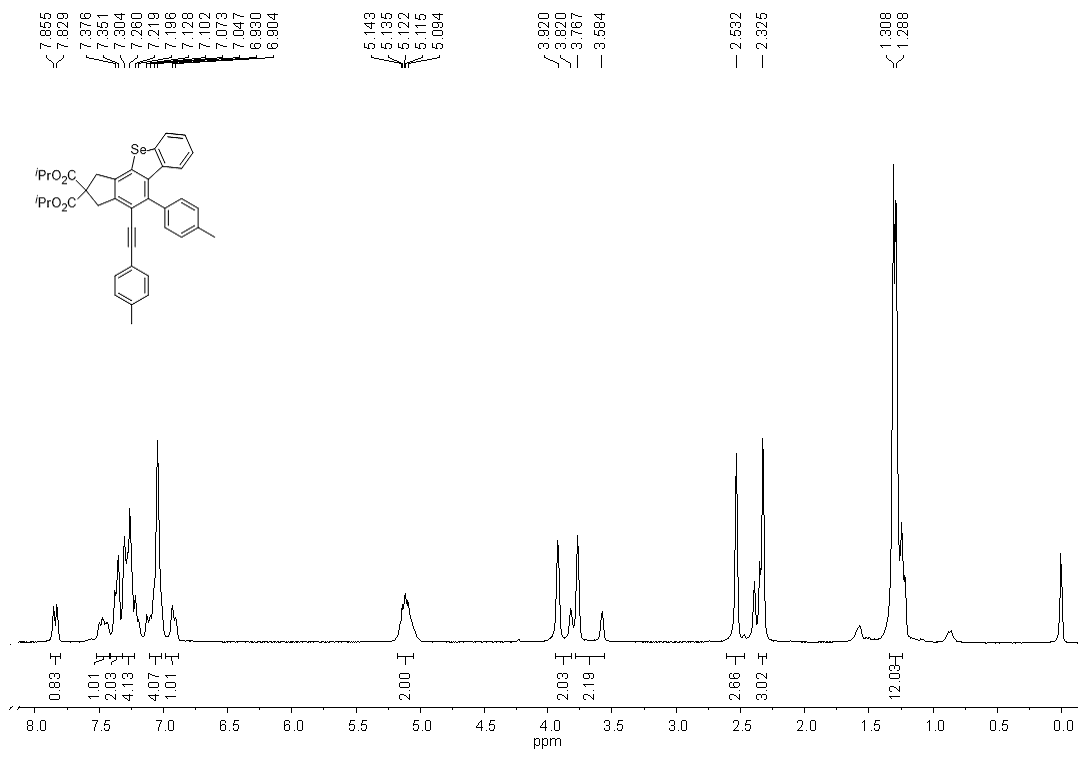
**


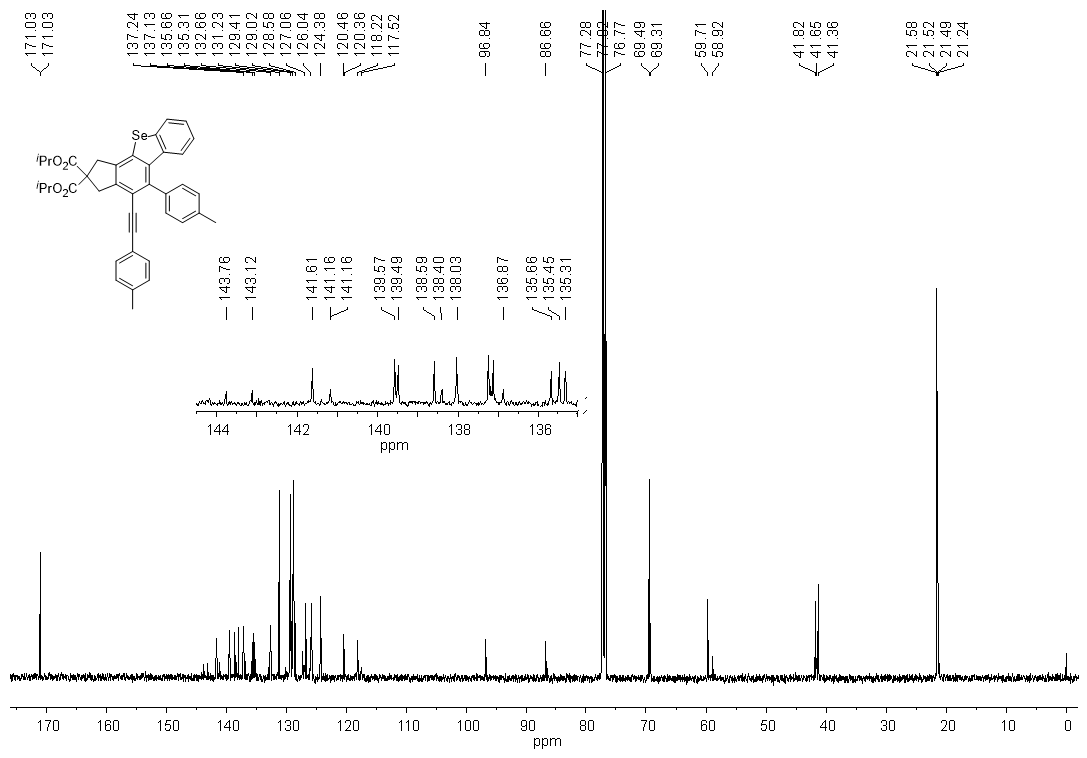


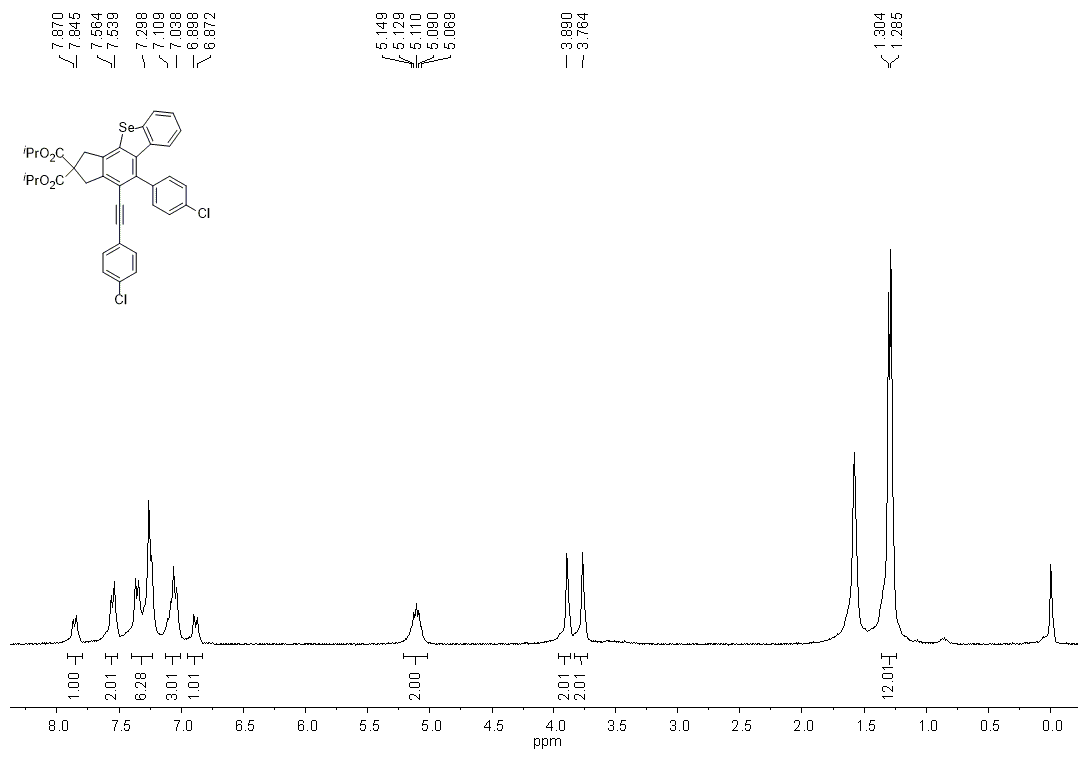

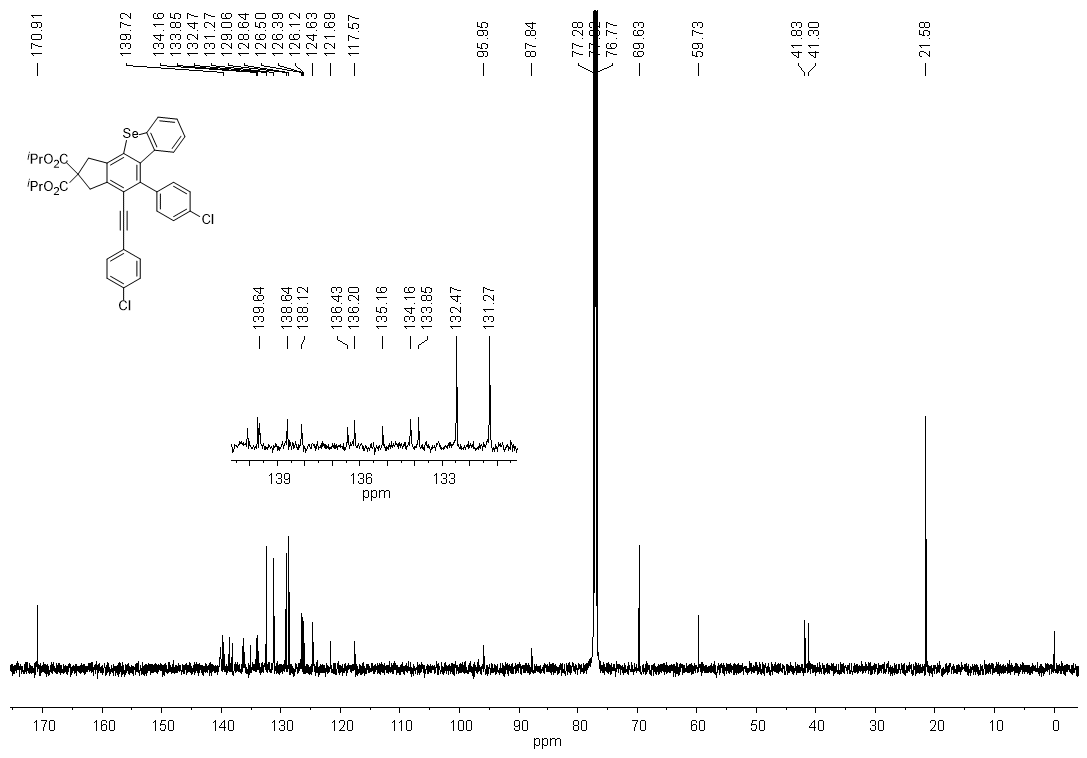


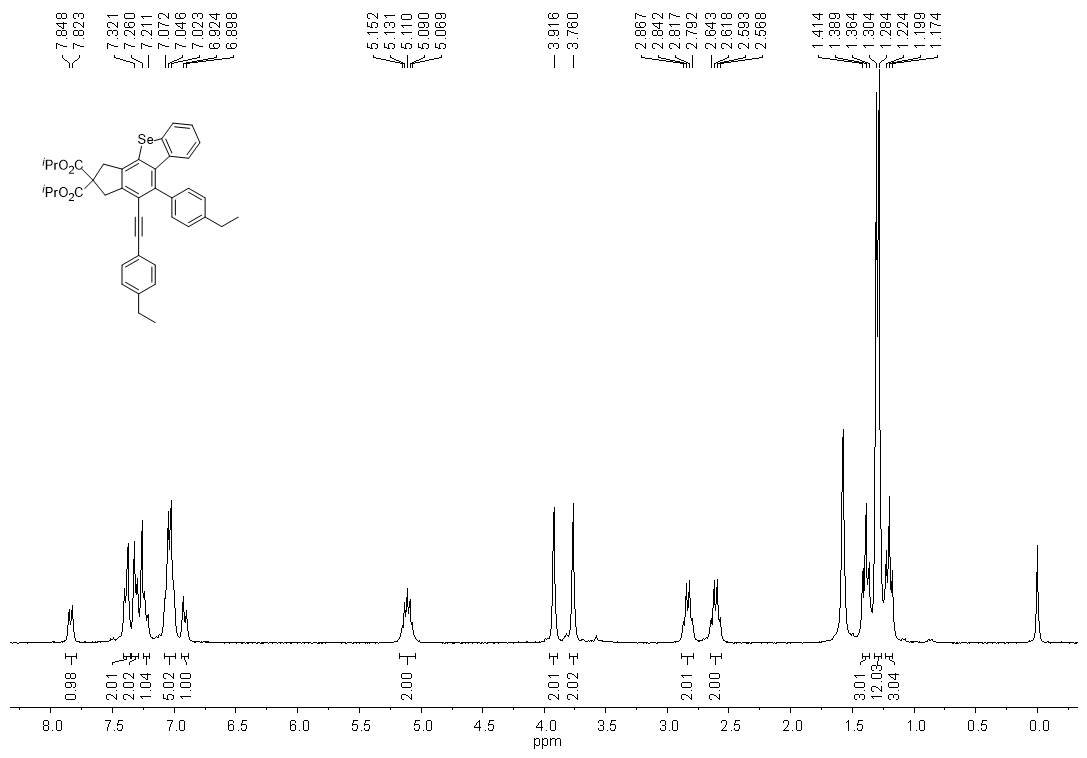

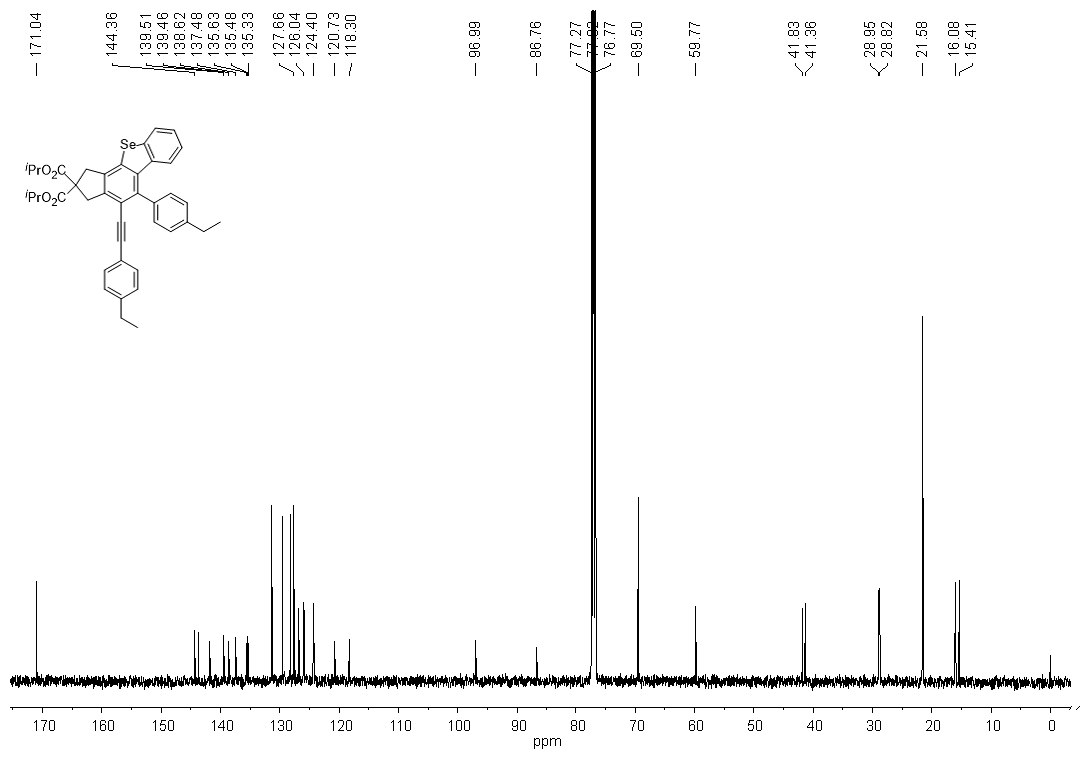


**
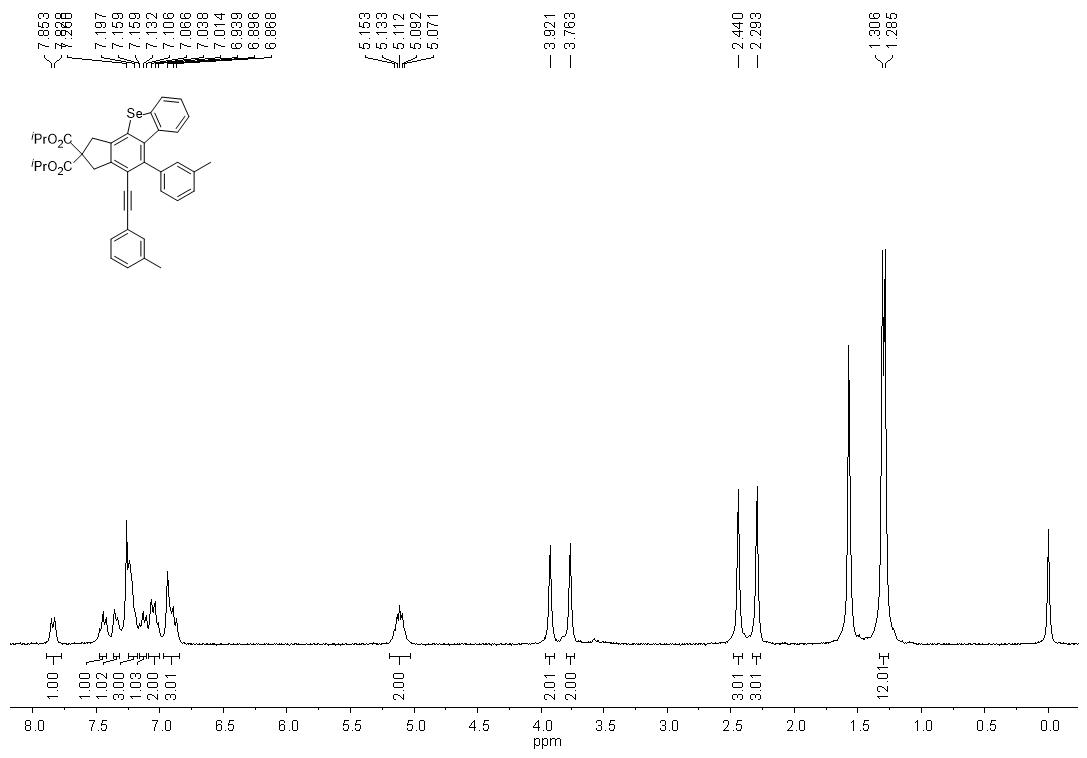
**


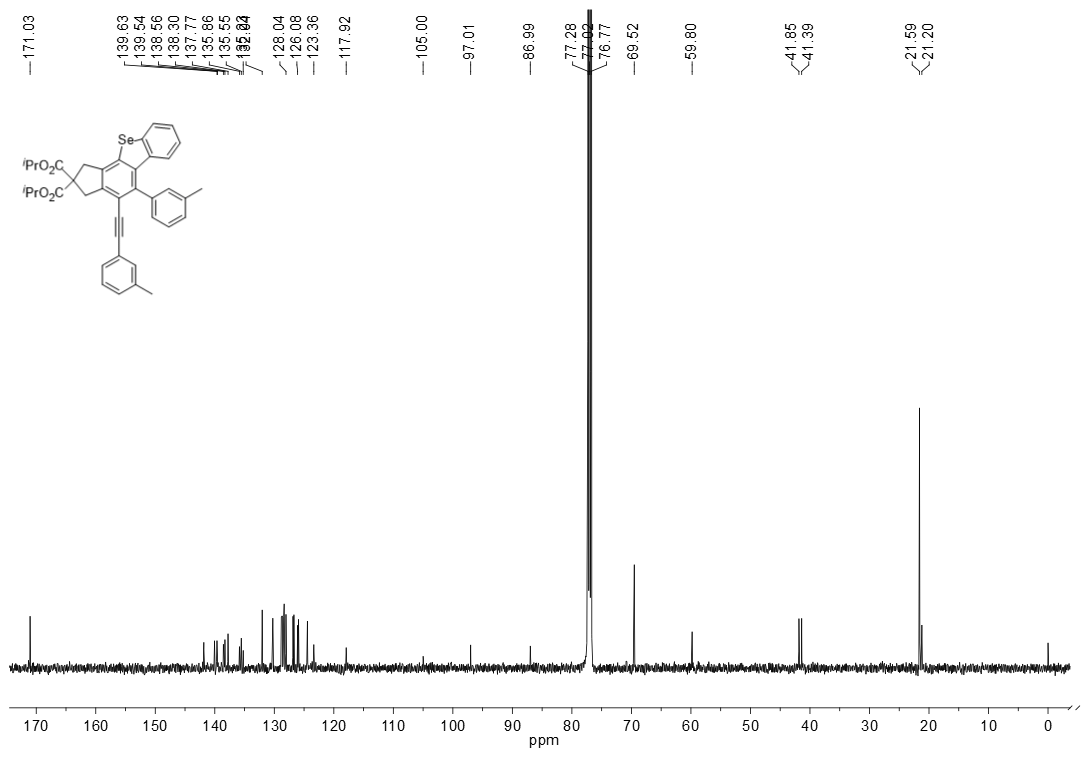


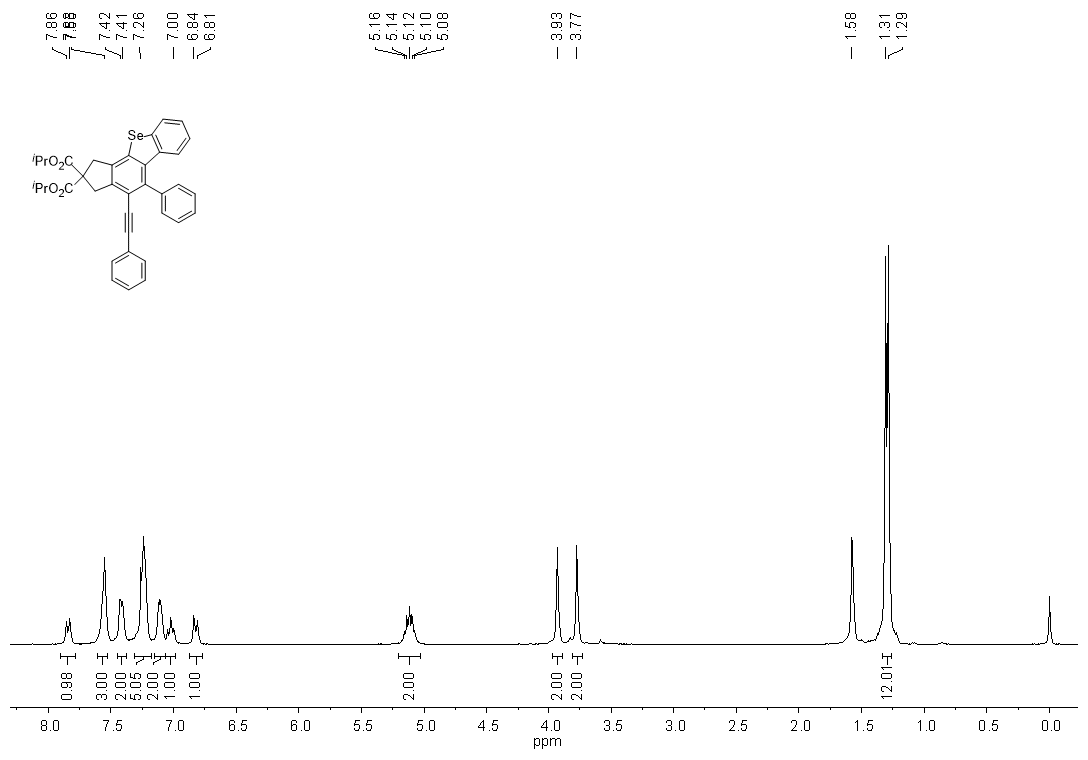


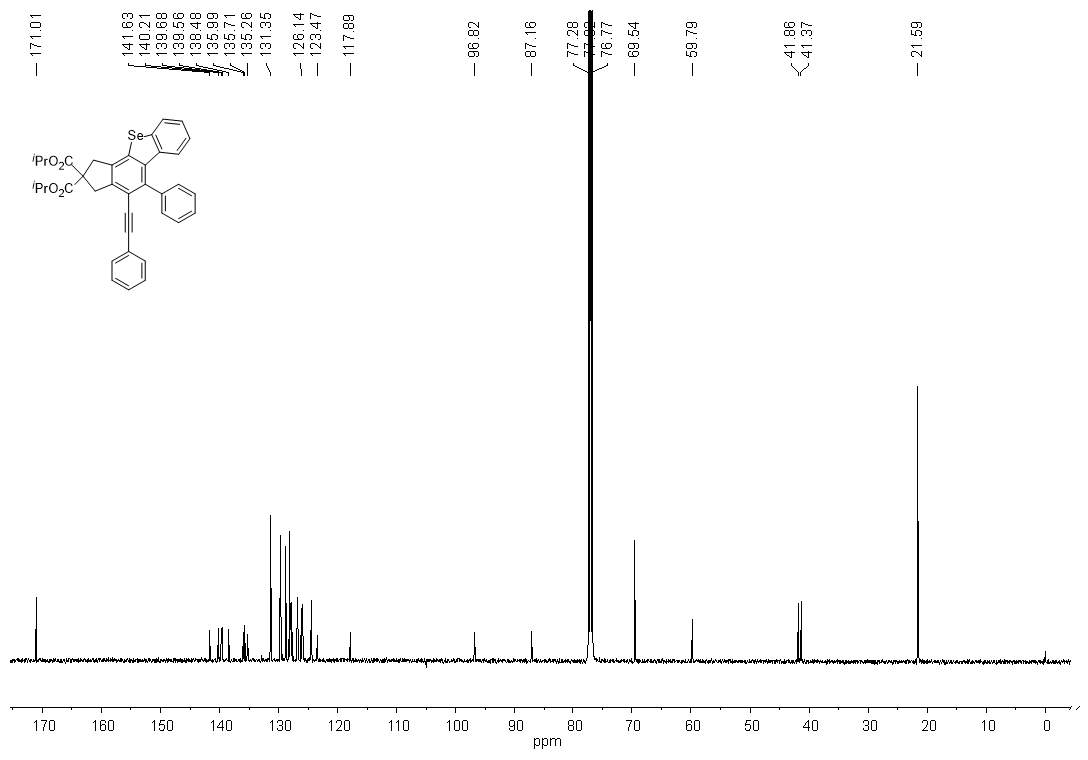


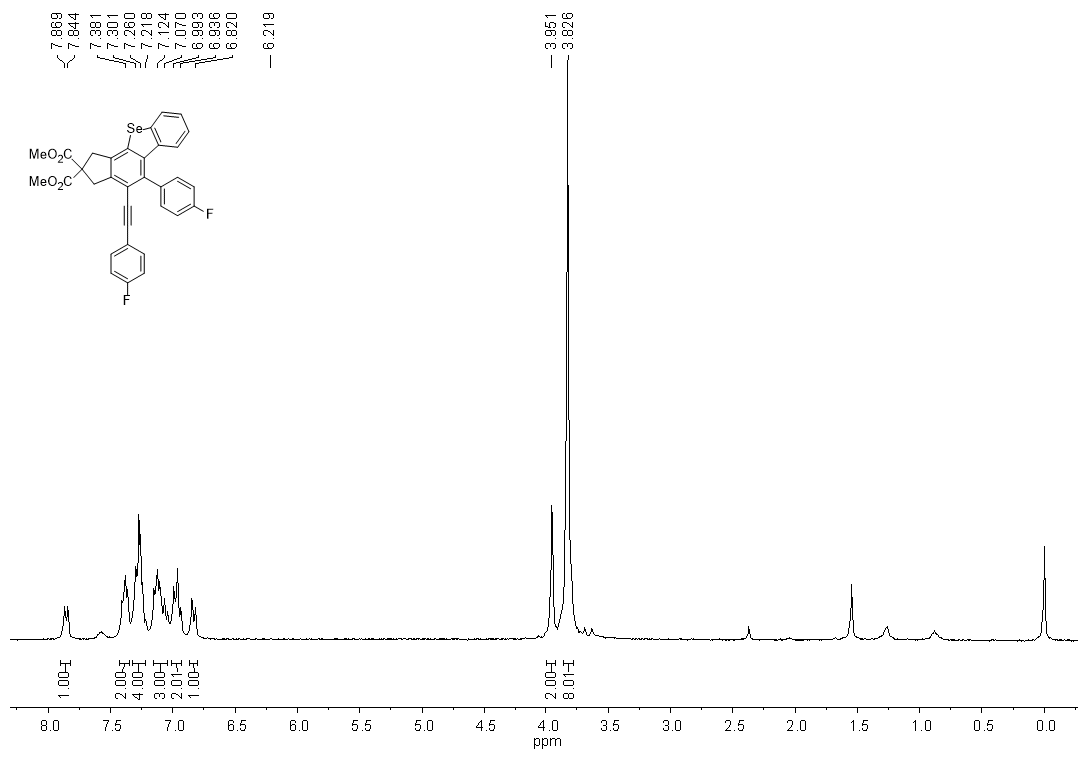

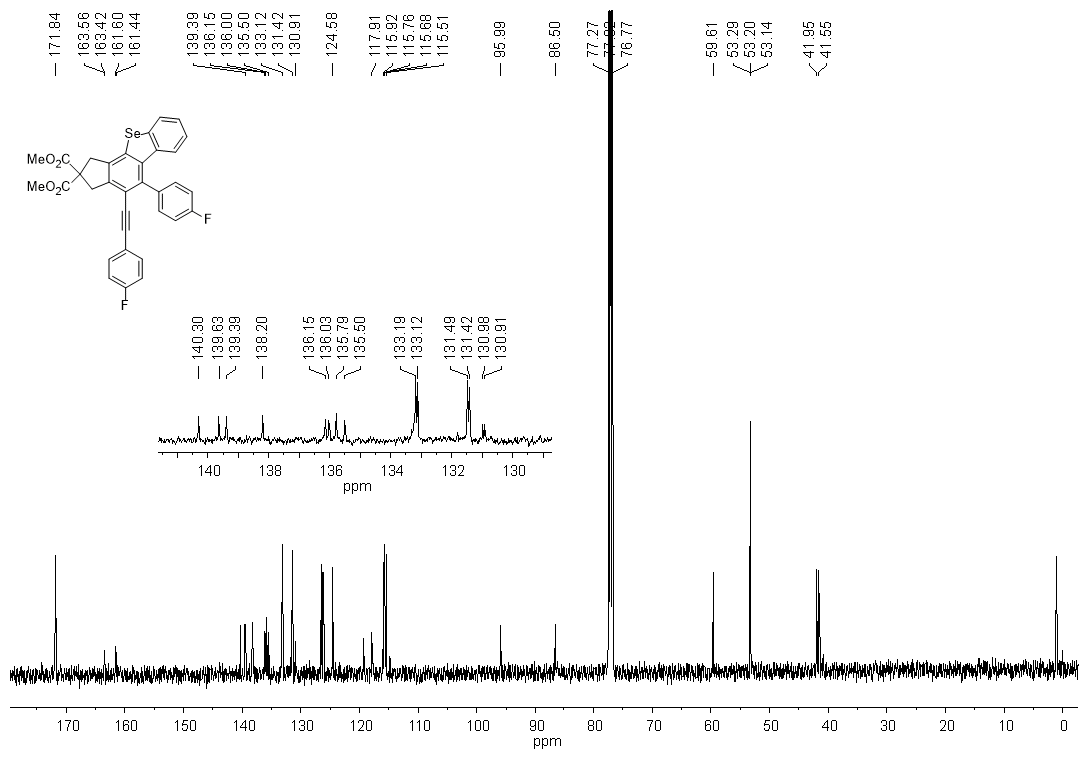


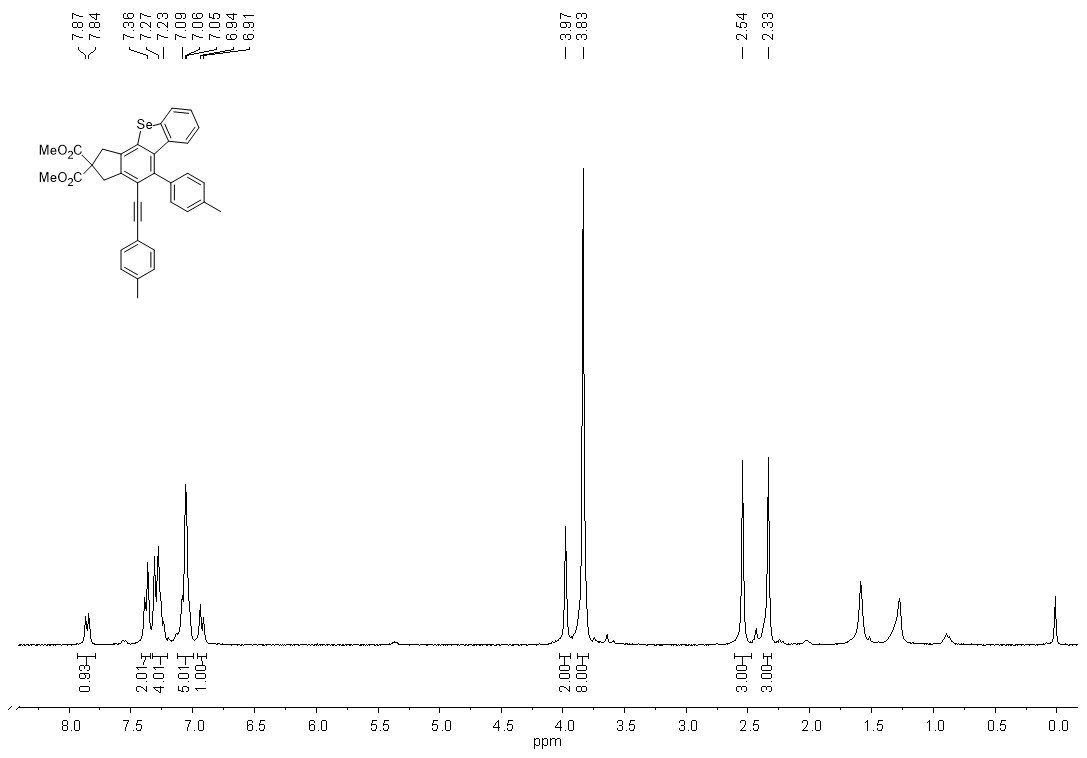


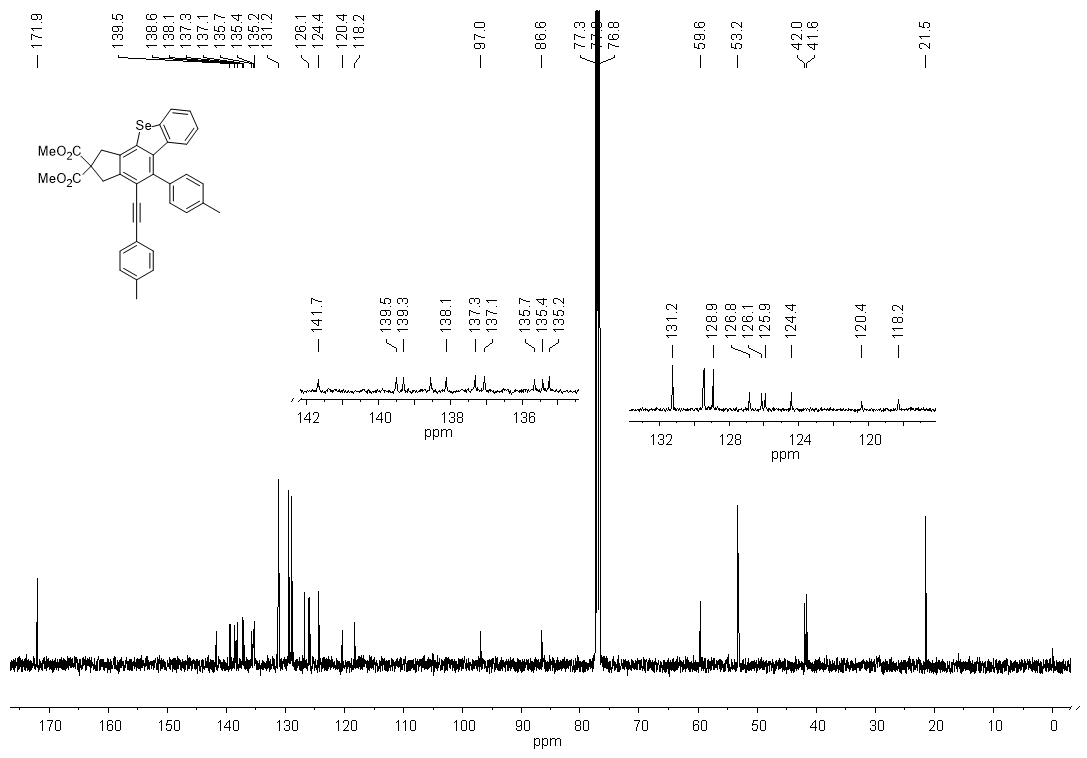


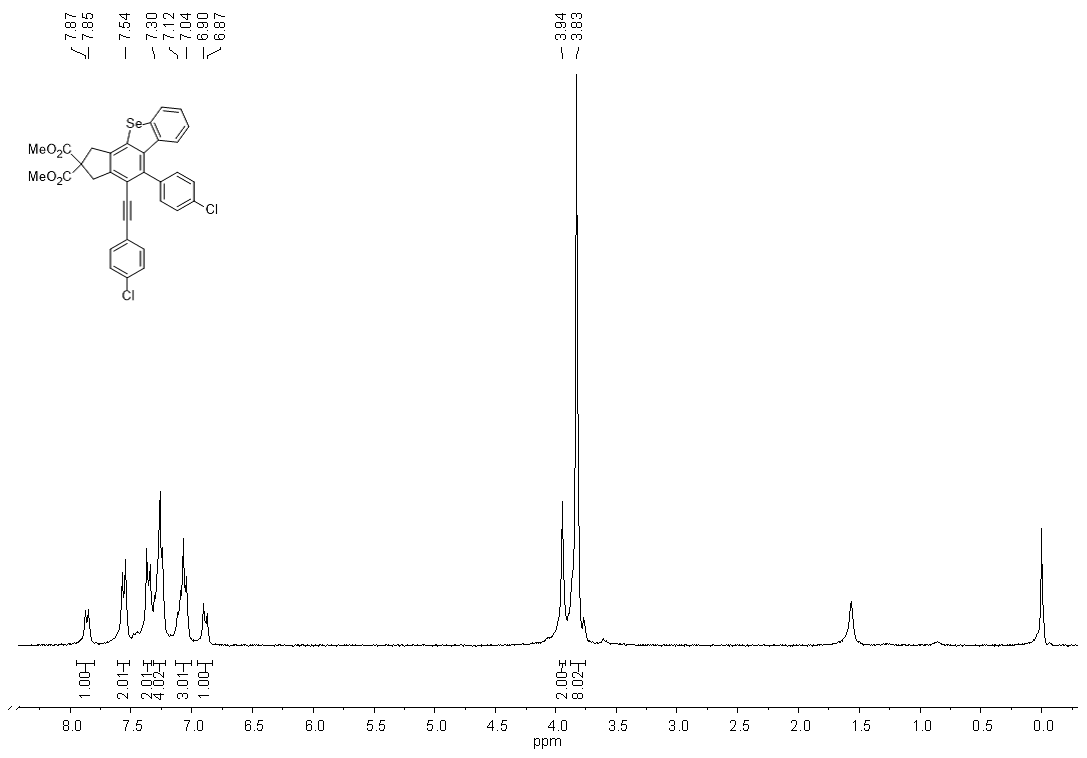

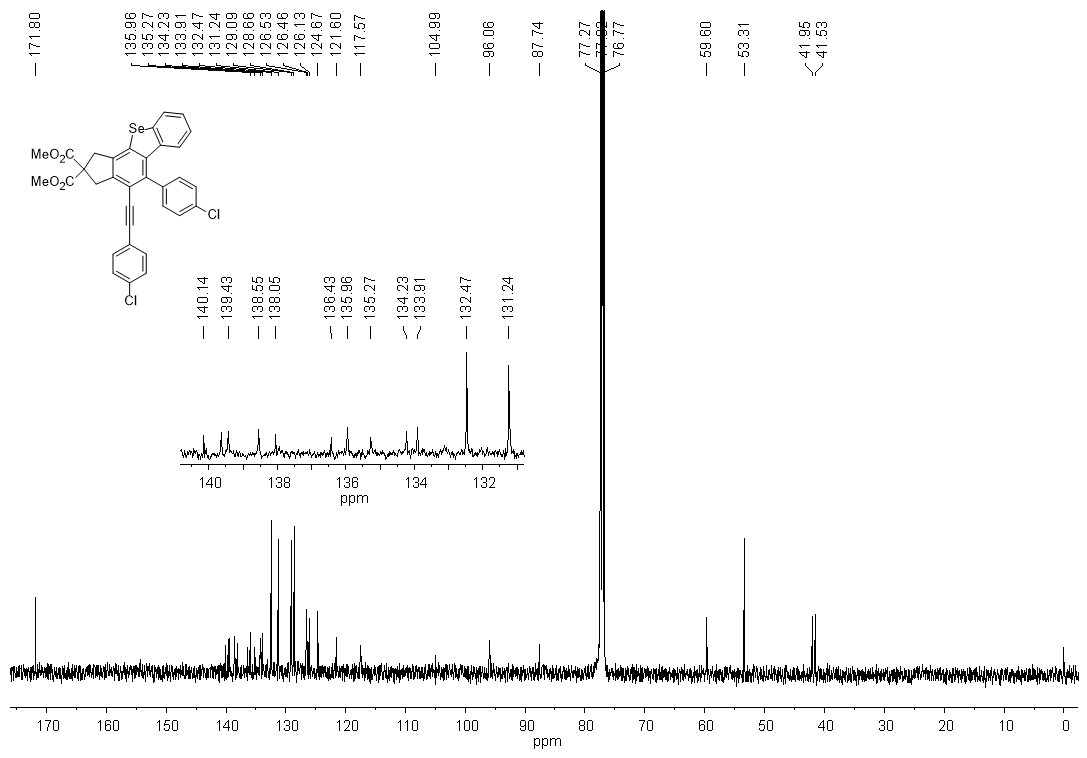


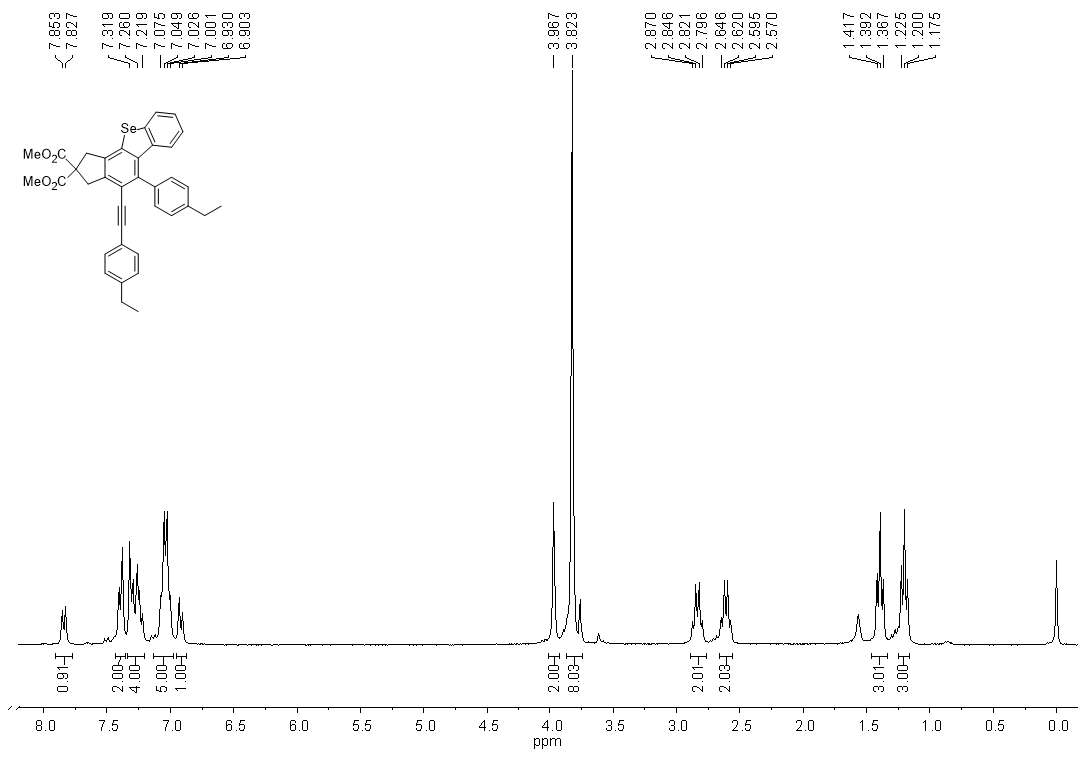


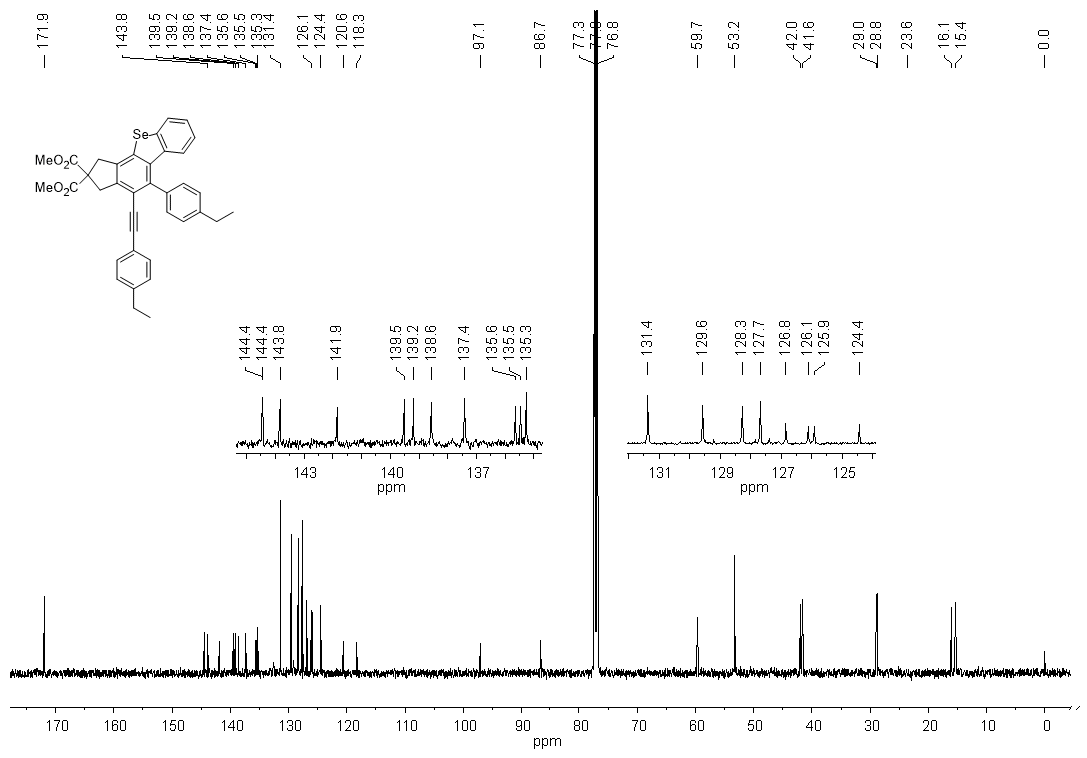


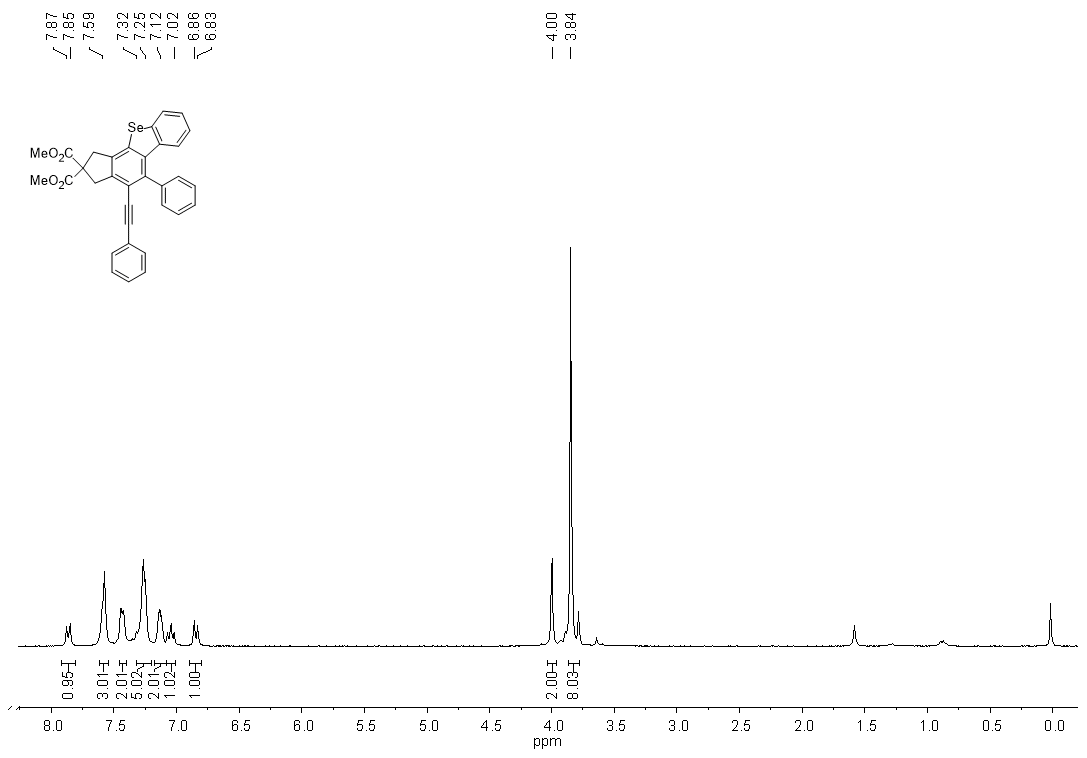

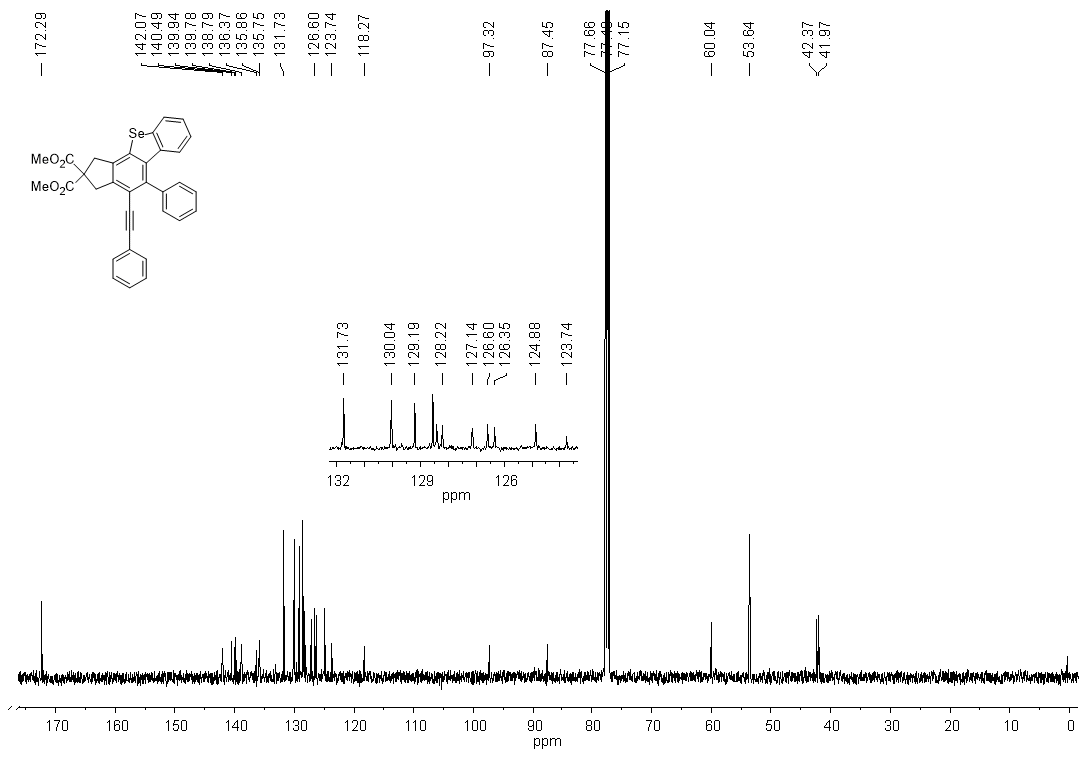

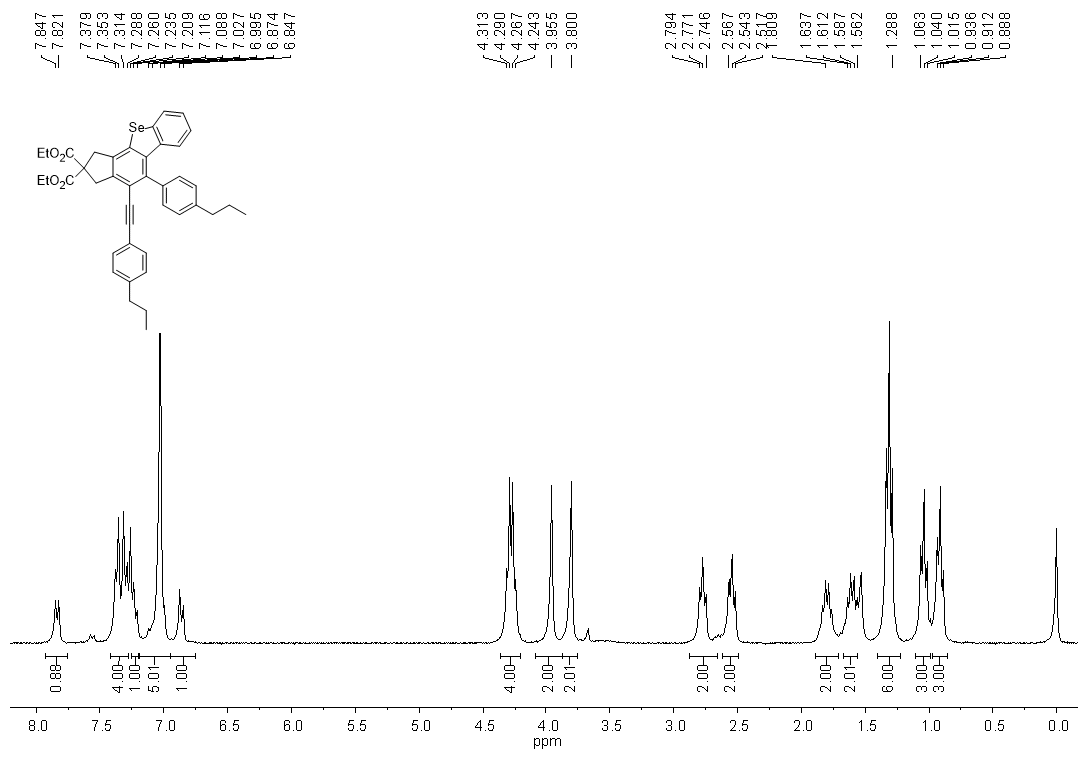

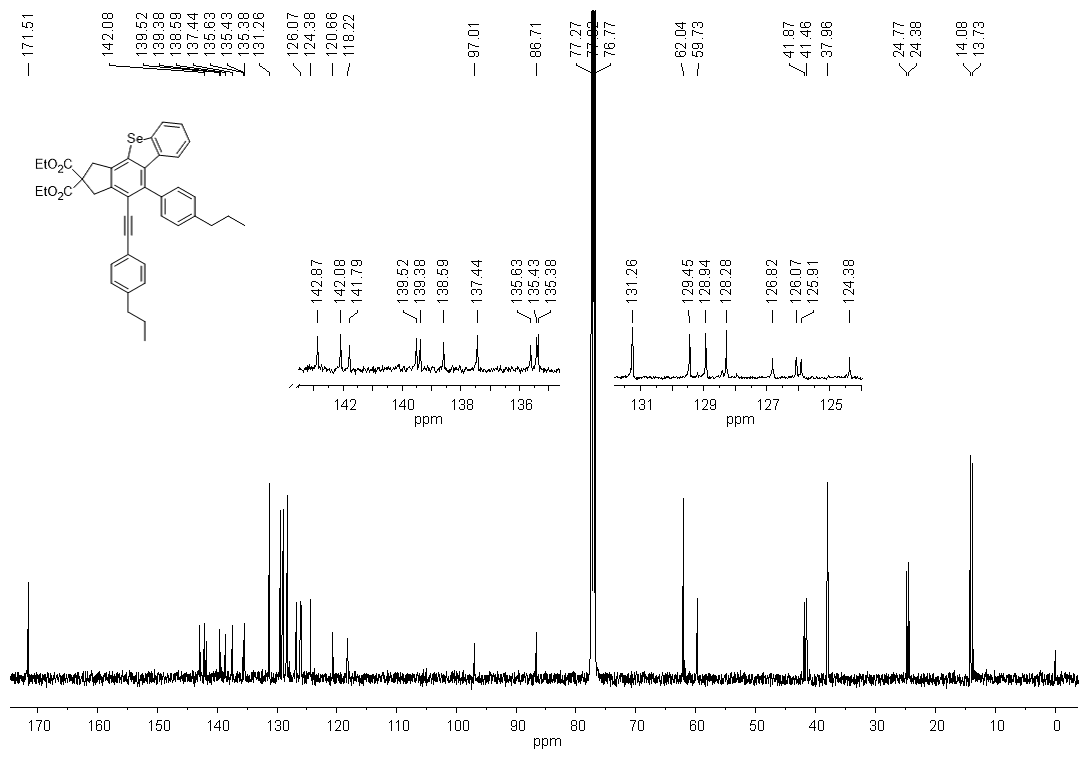

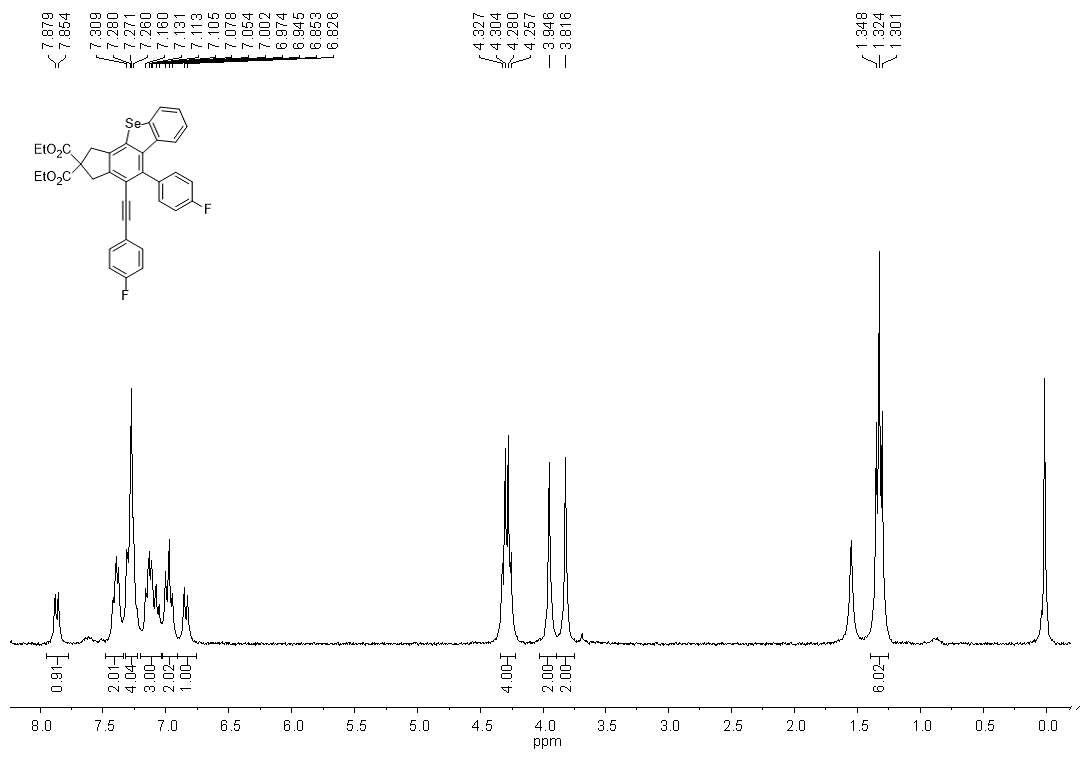

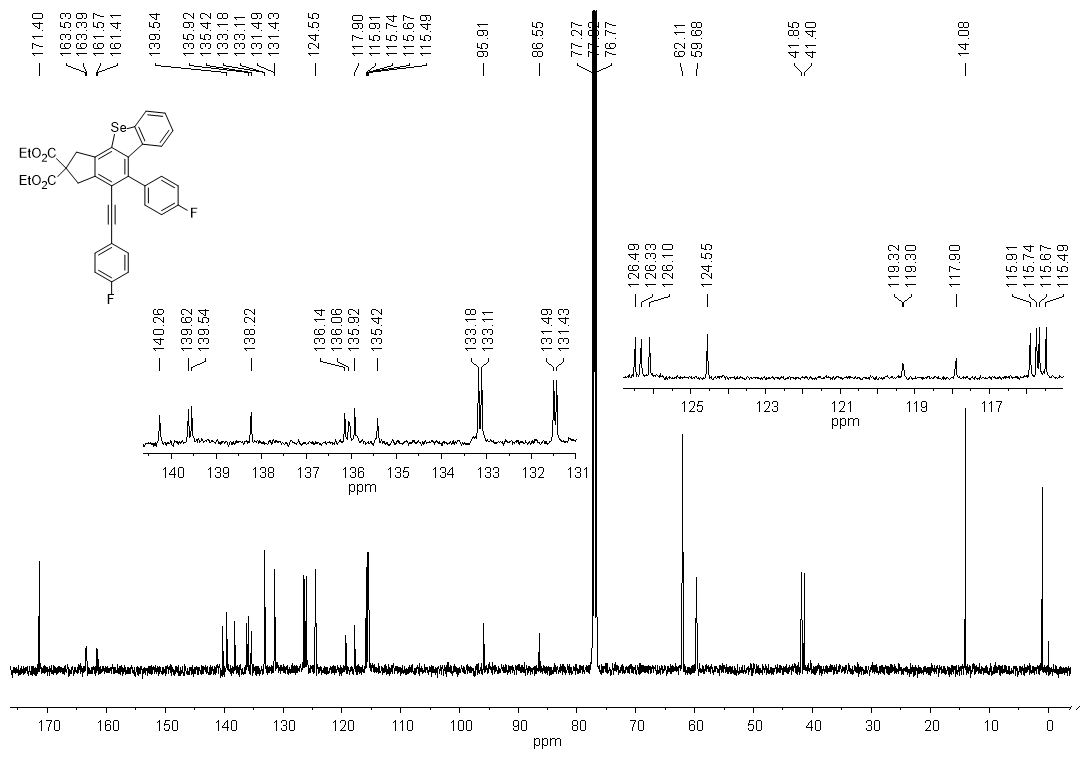

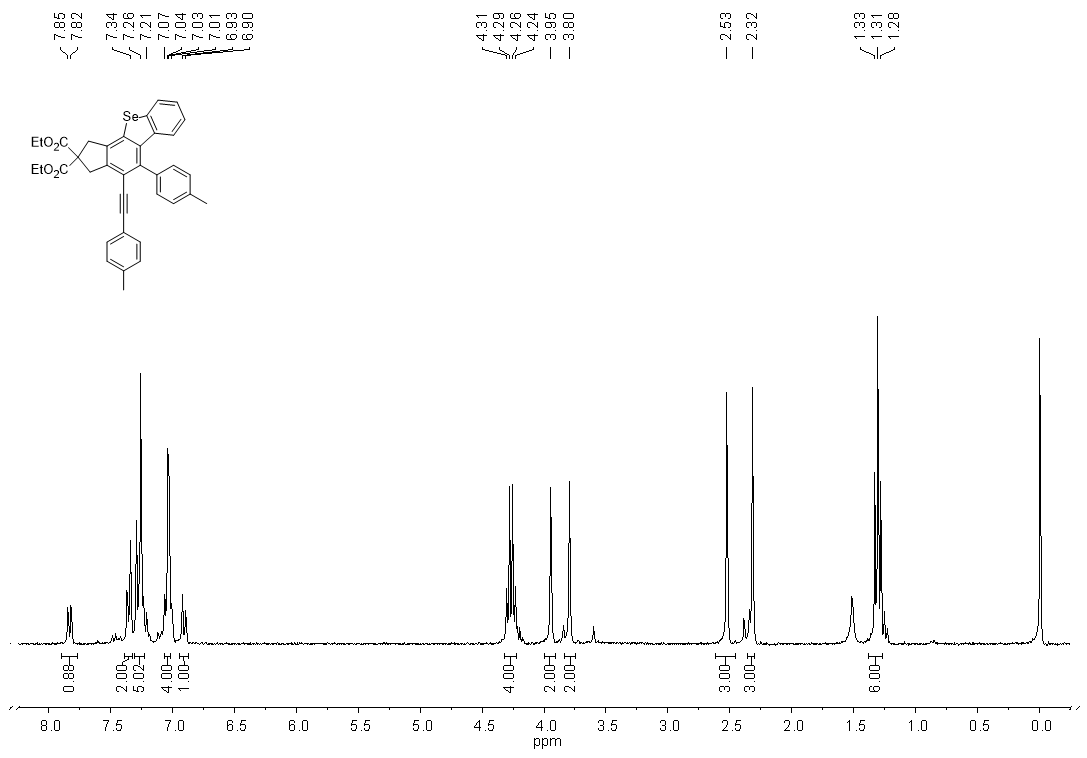

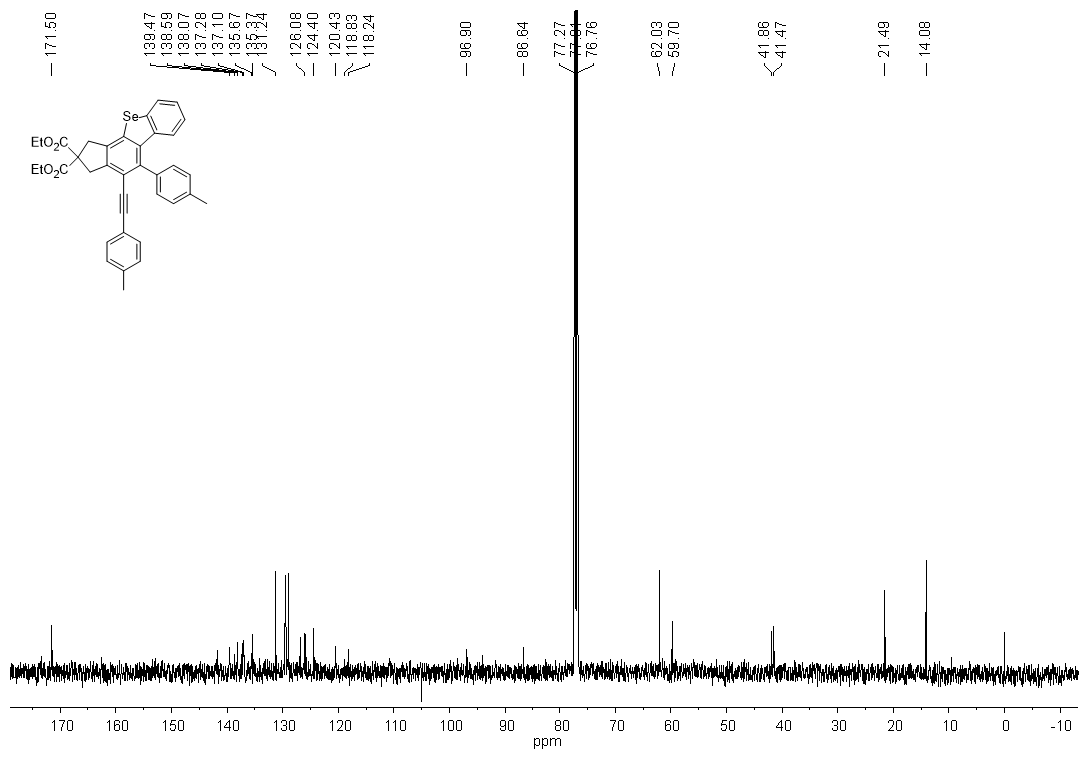


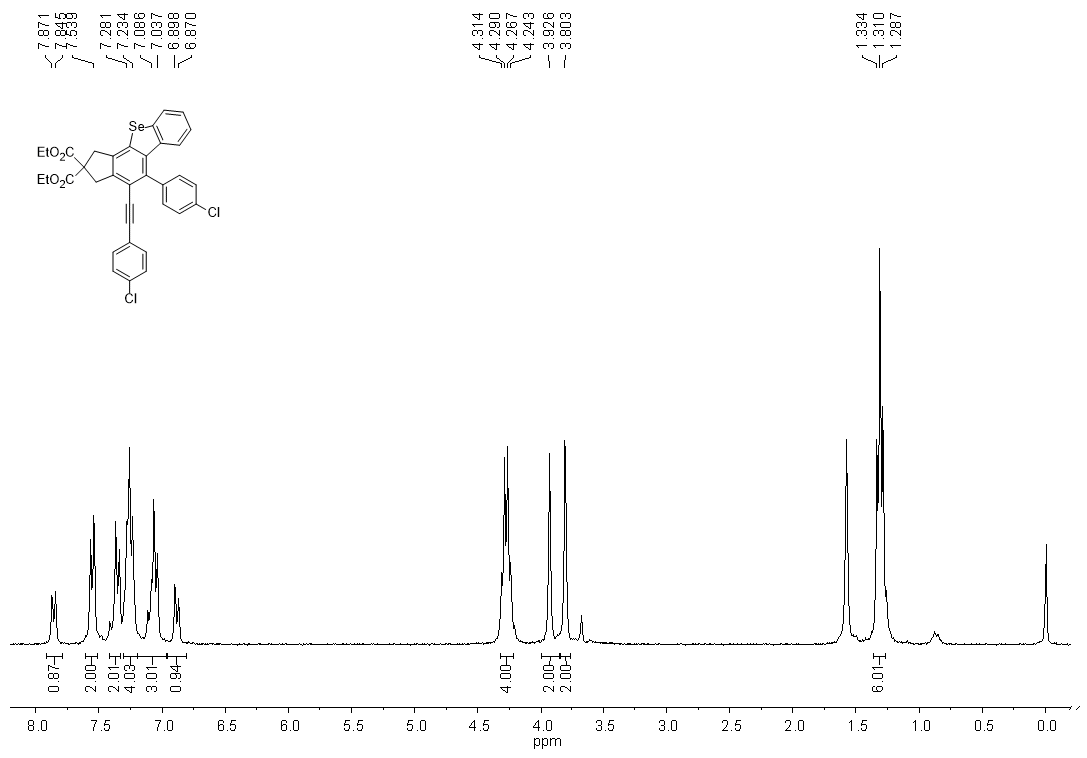


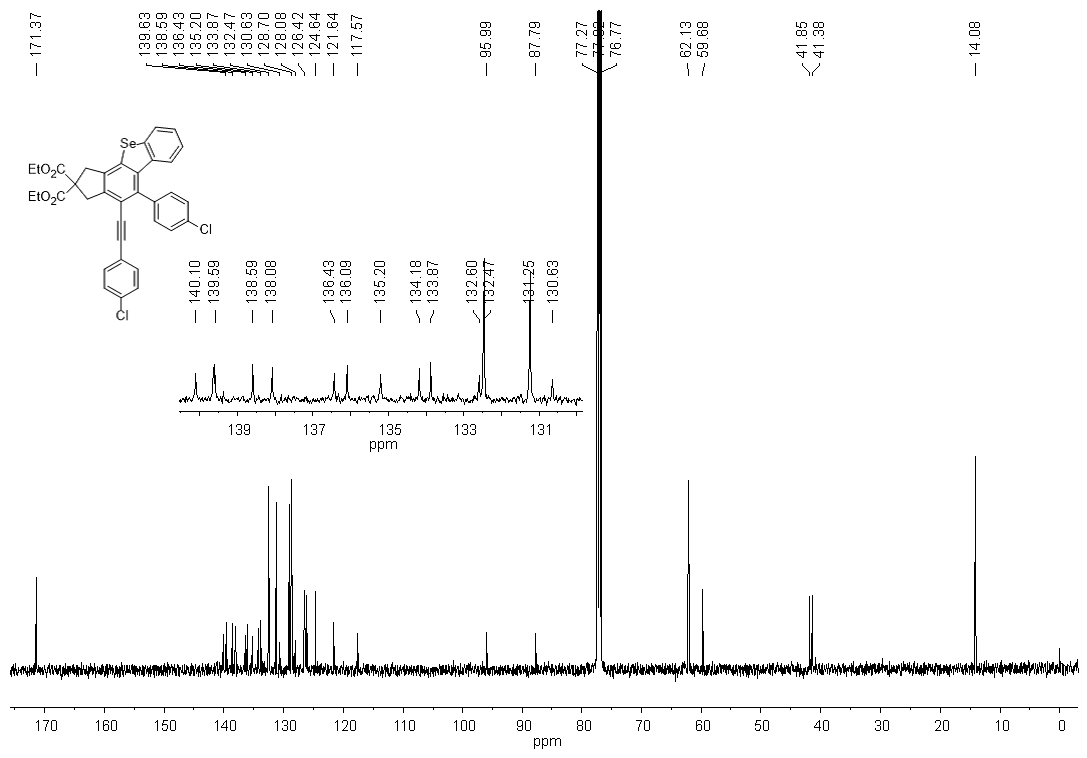


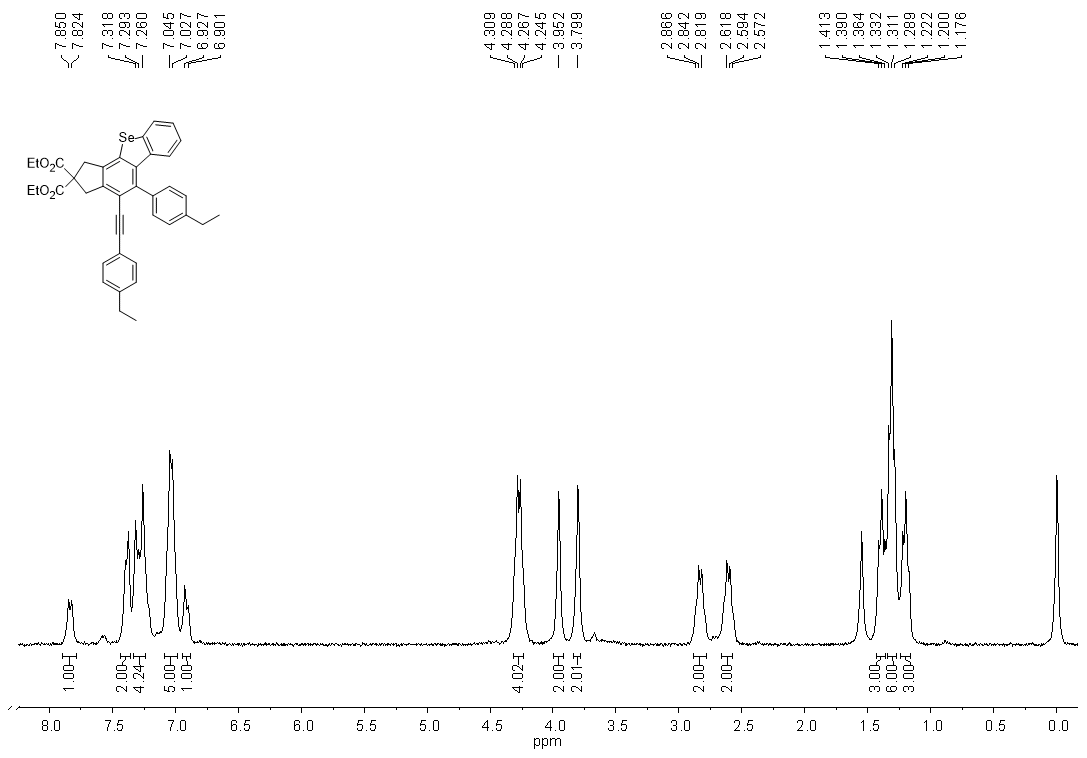

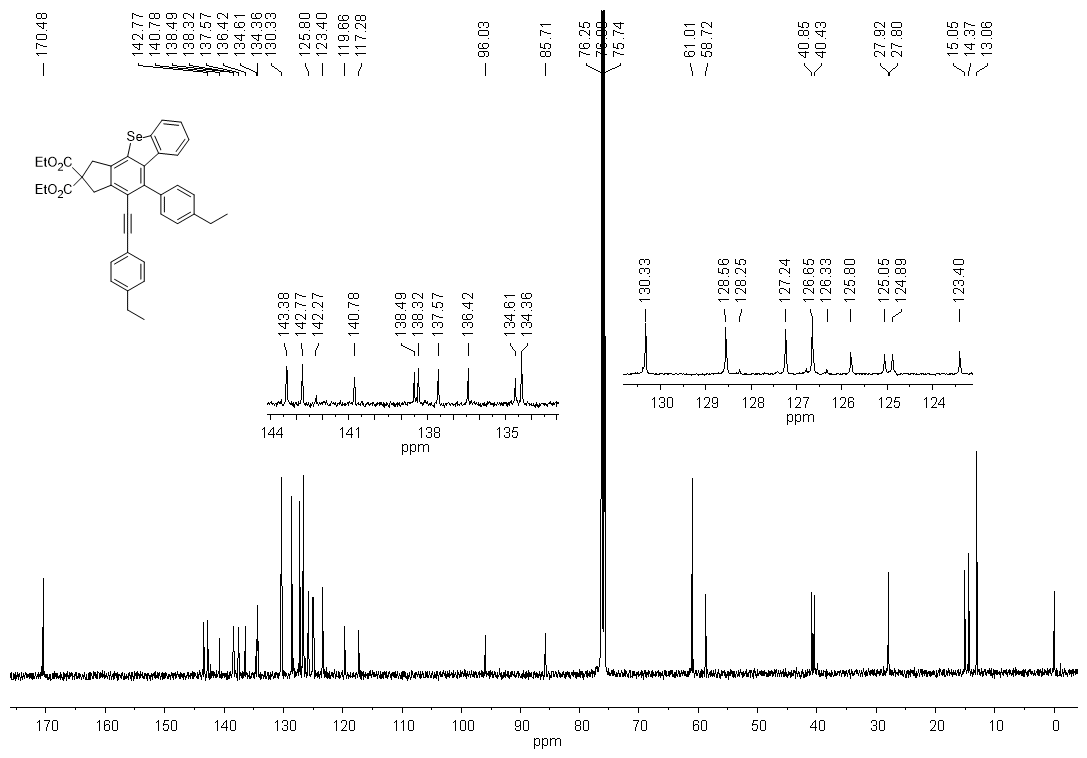

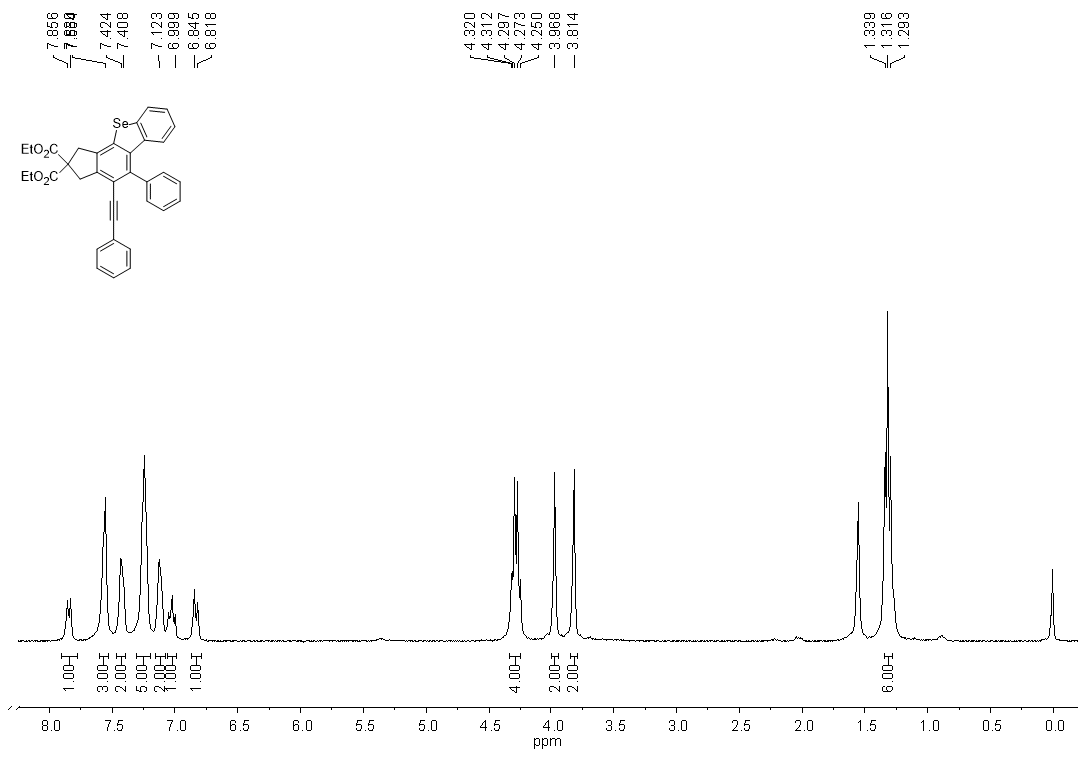


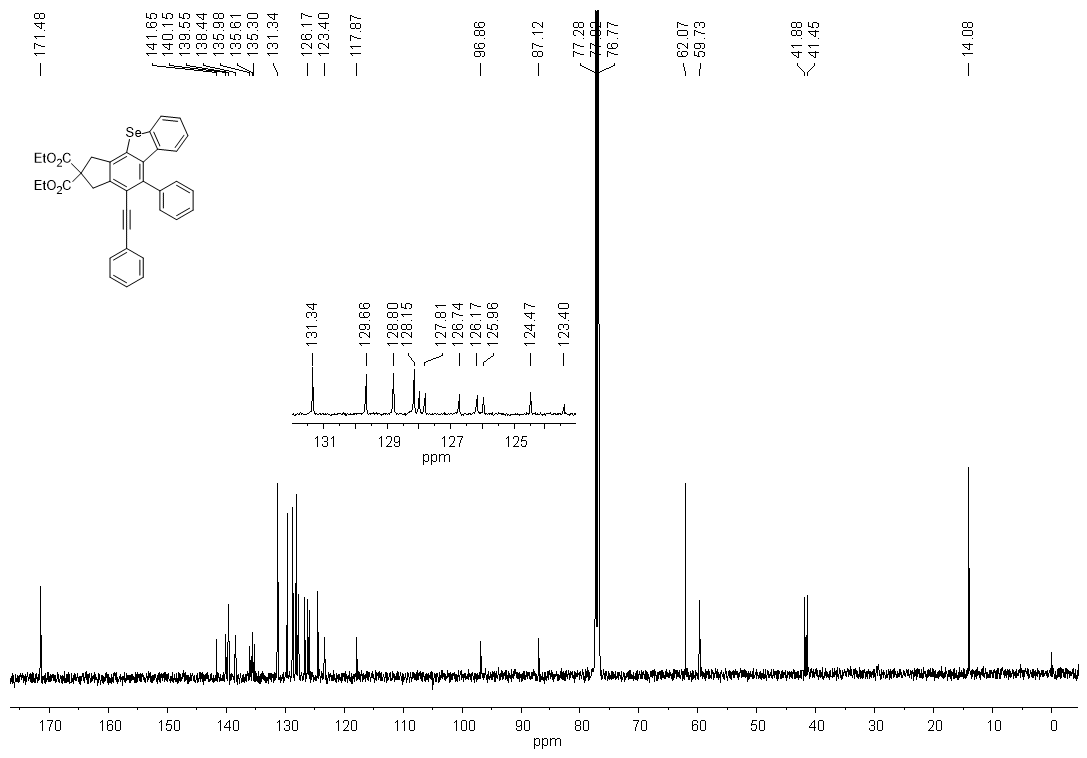


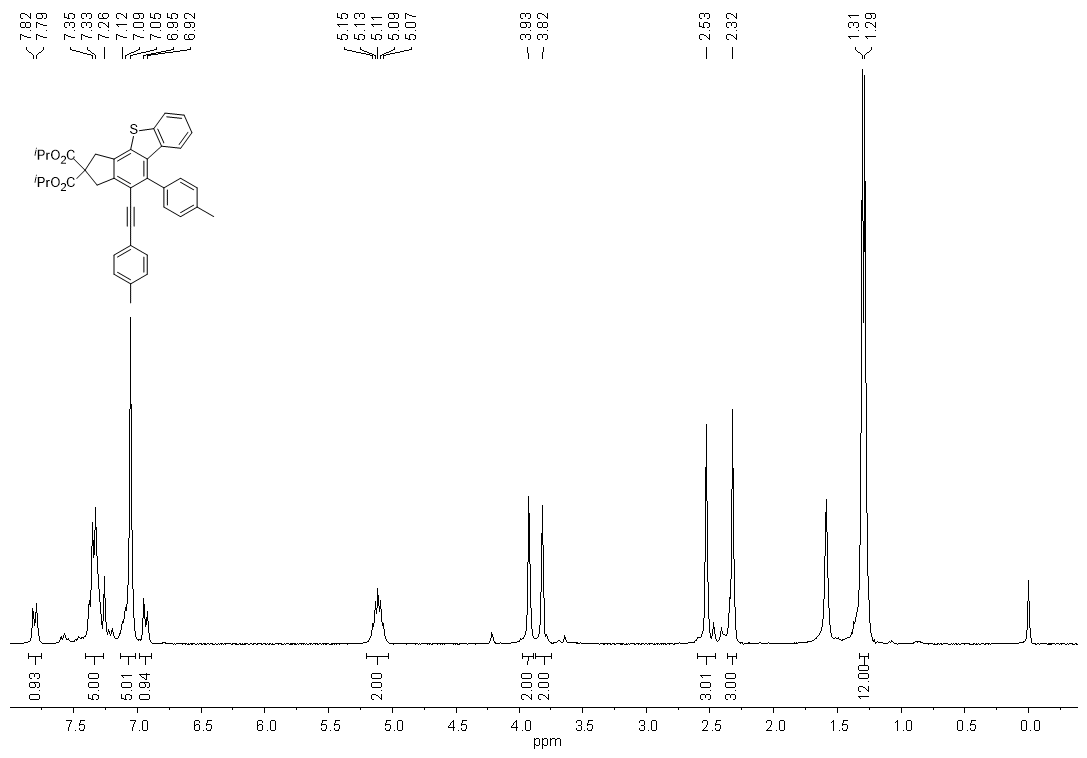

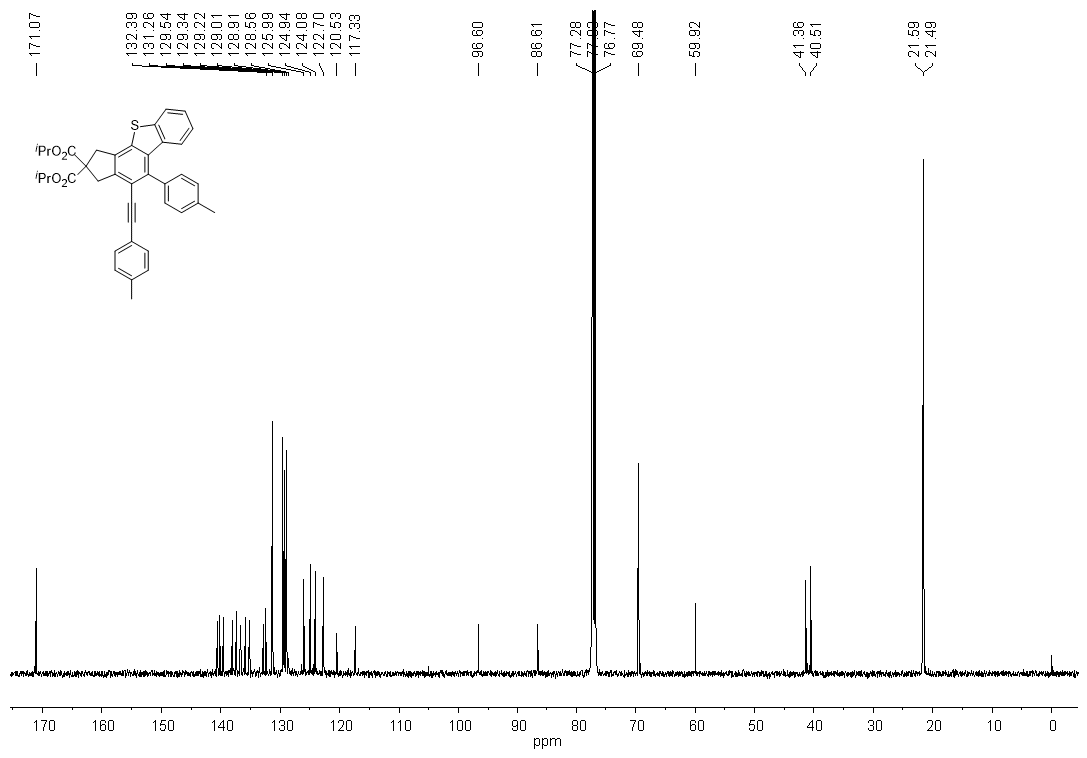

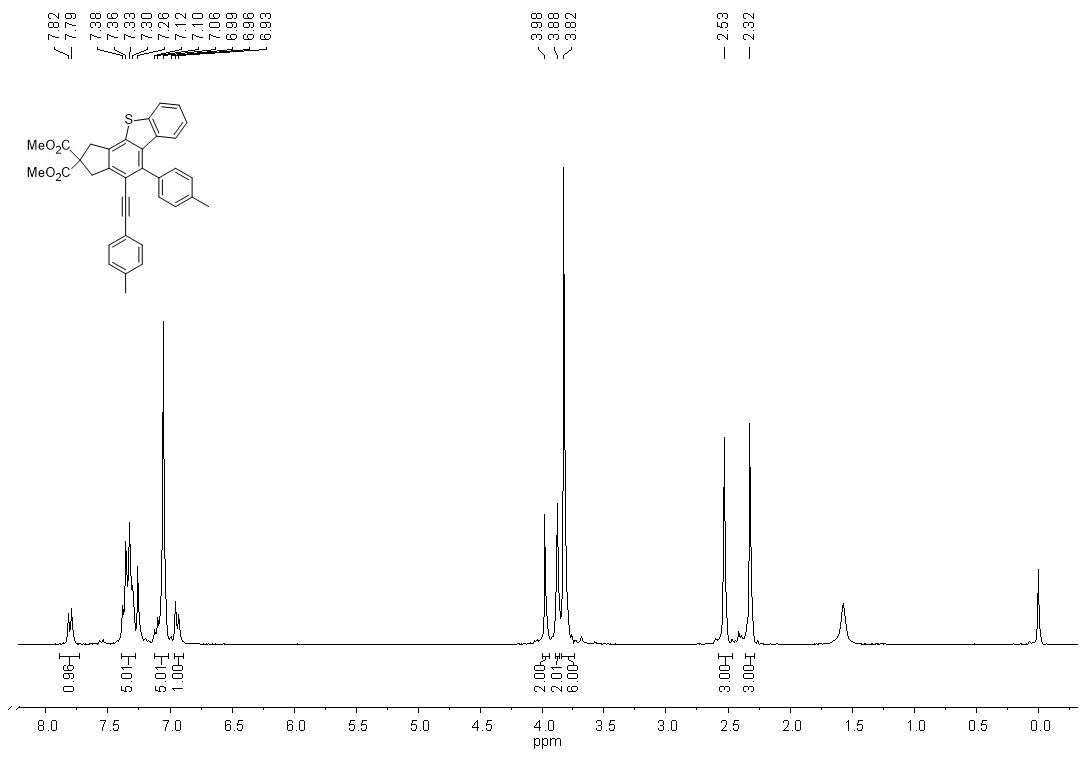


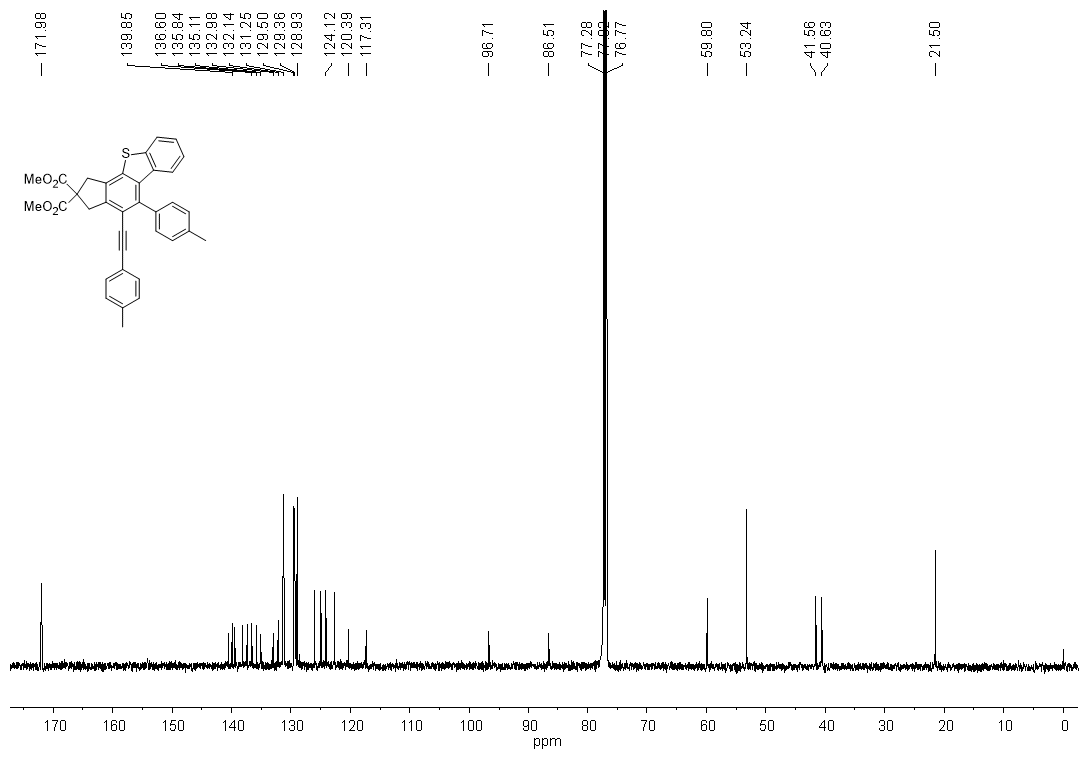


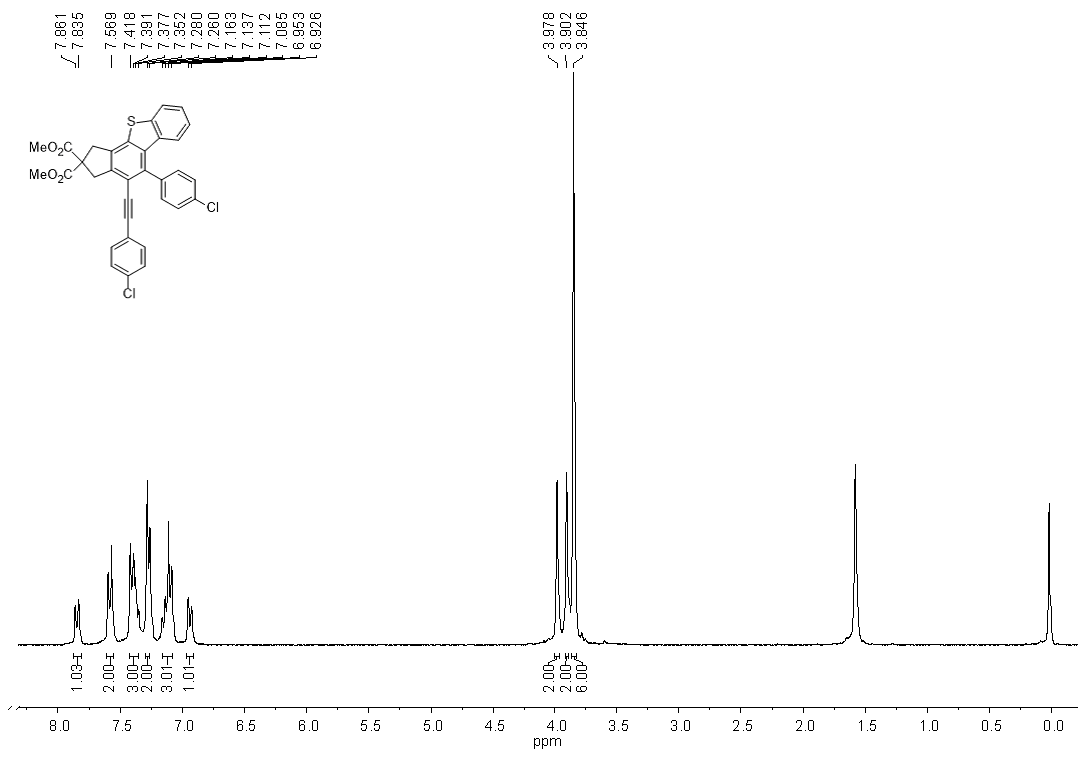

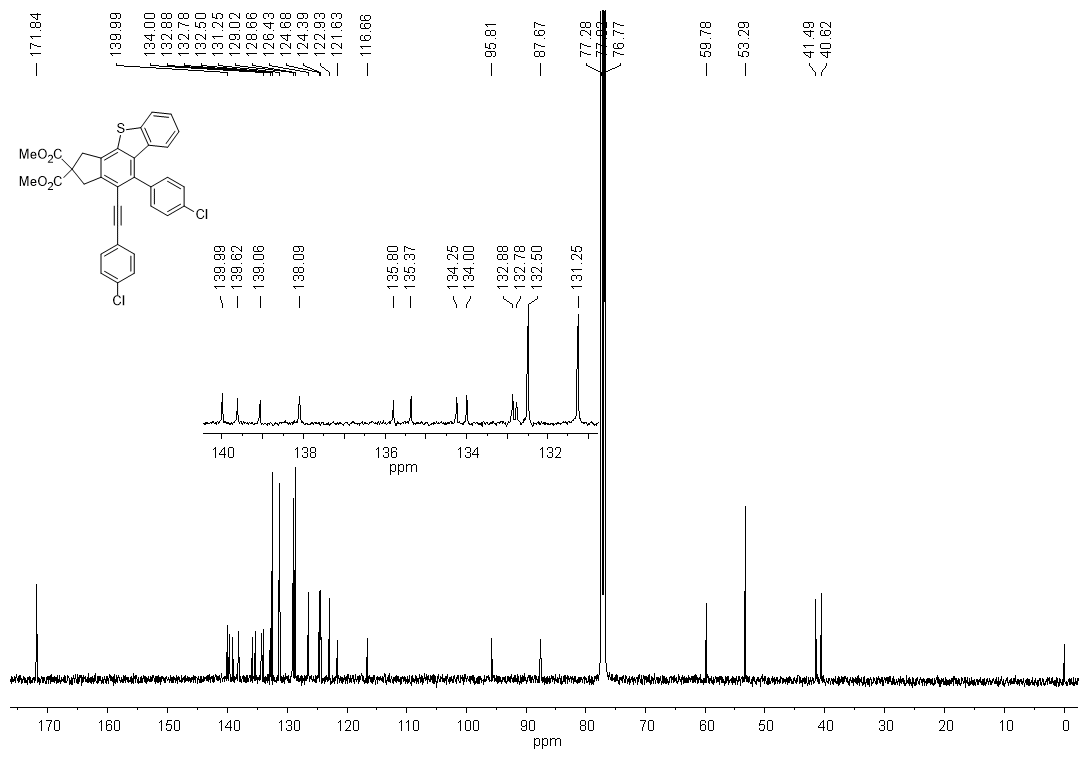

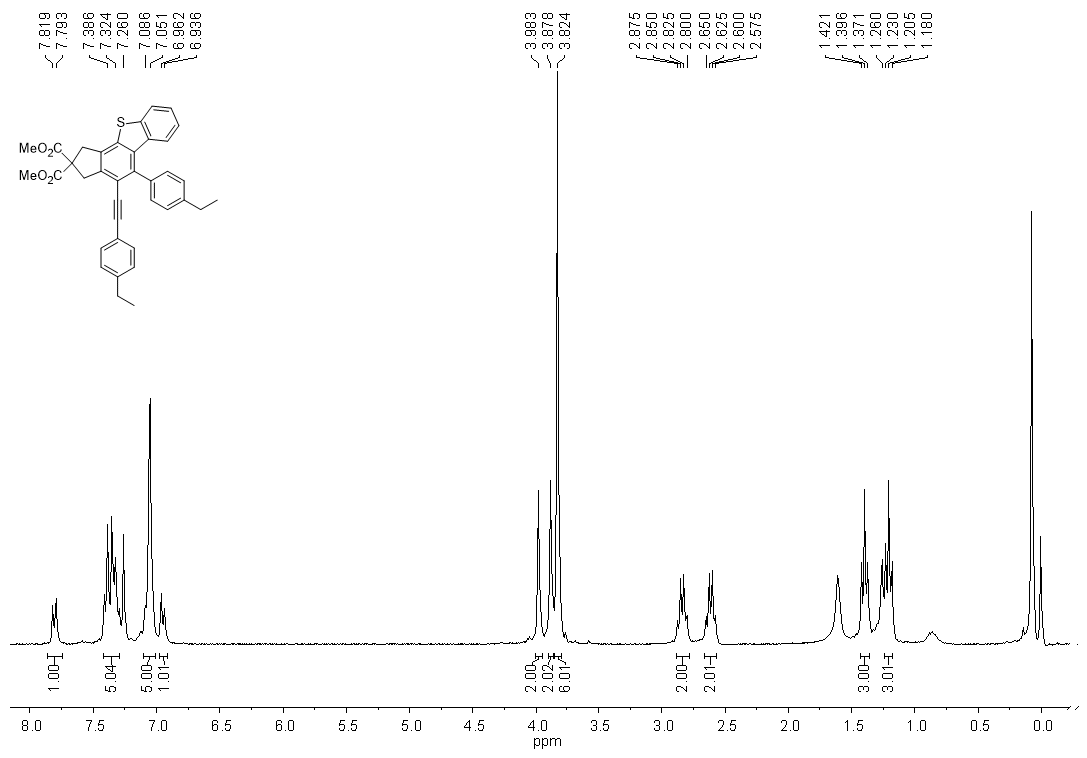

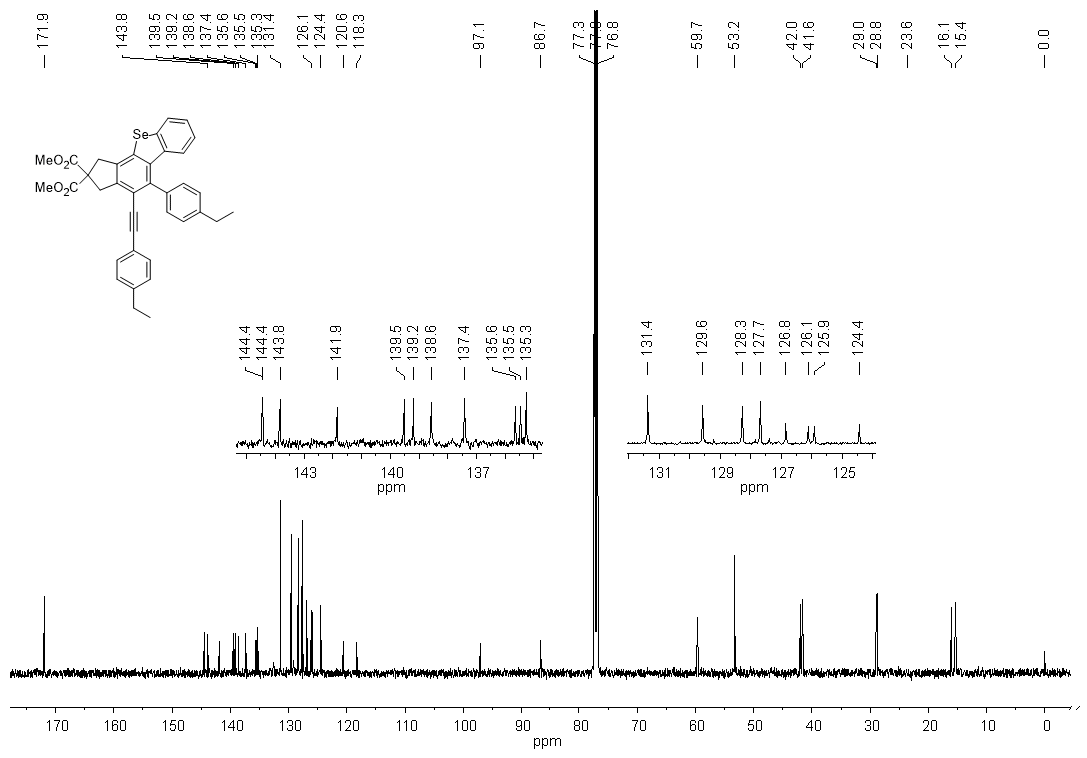

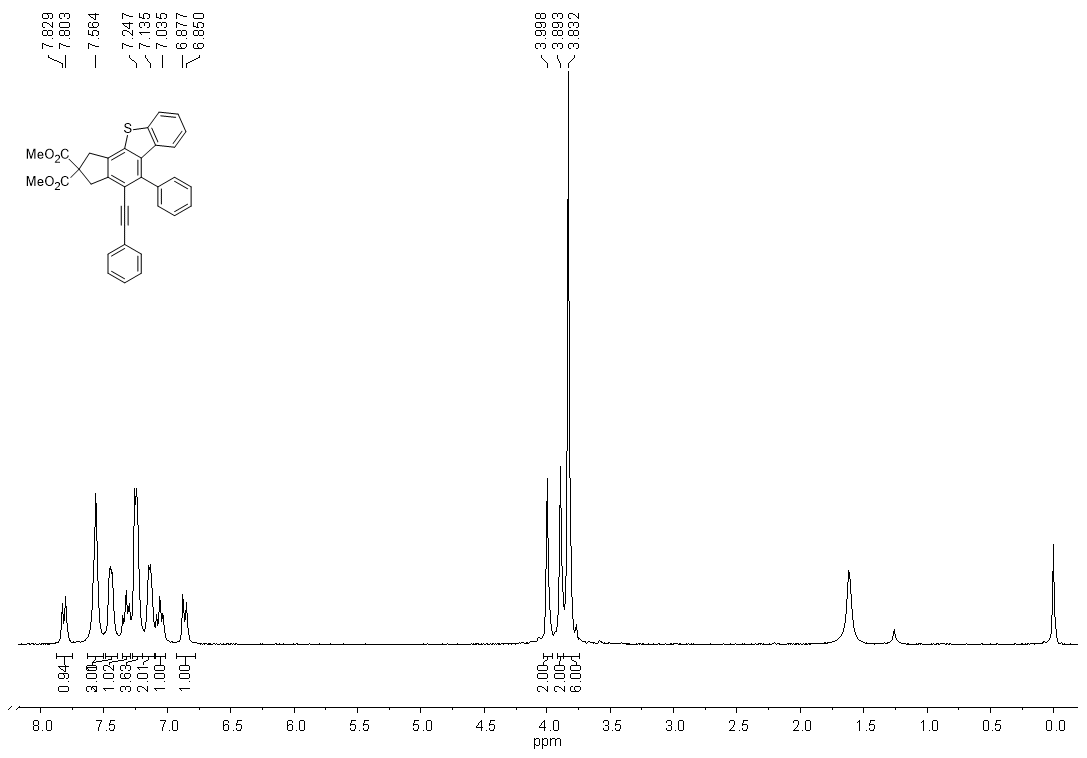


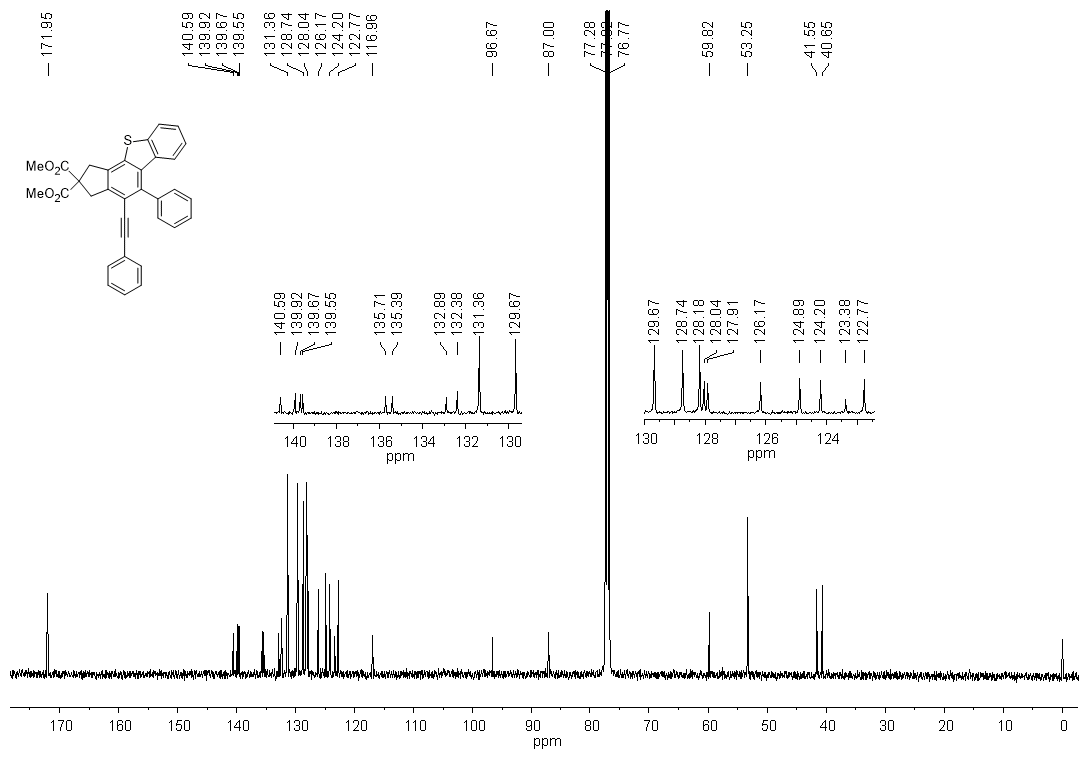


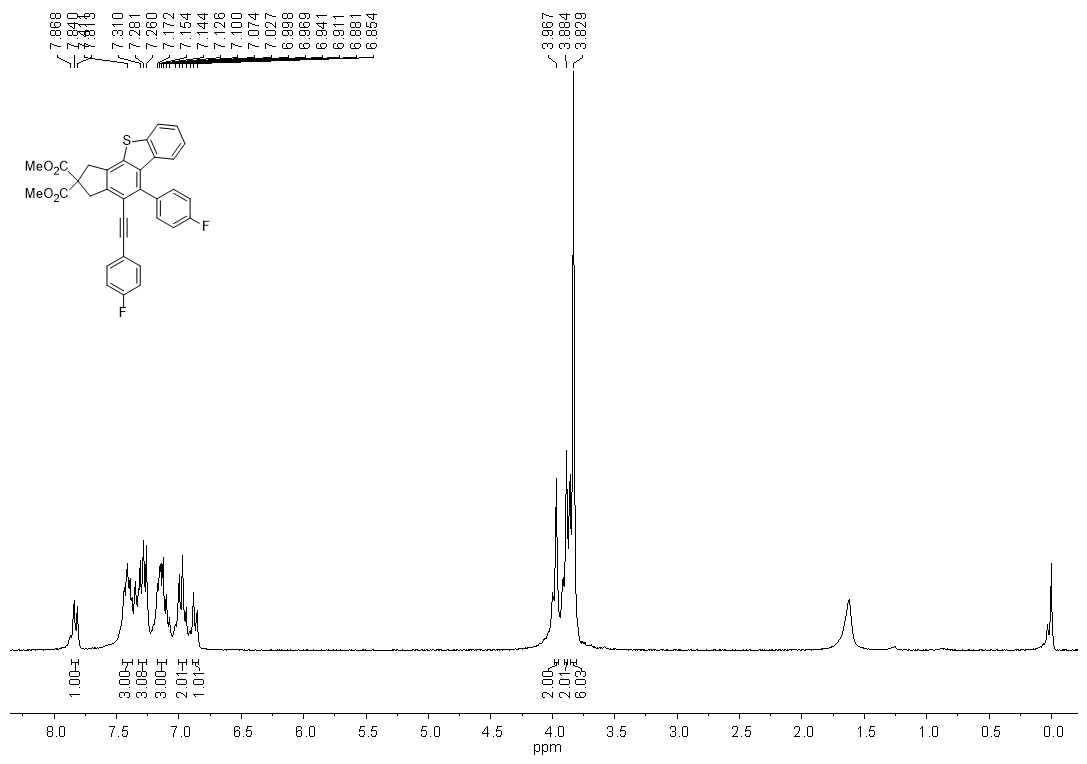


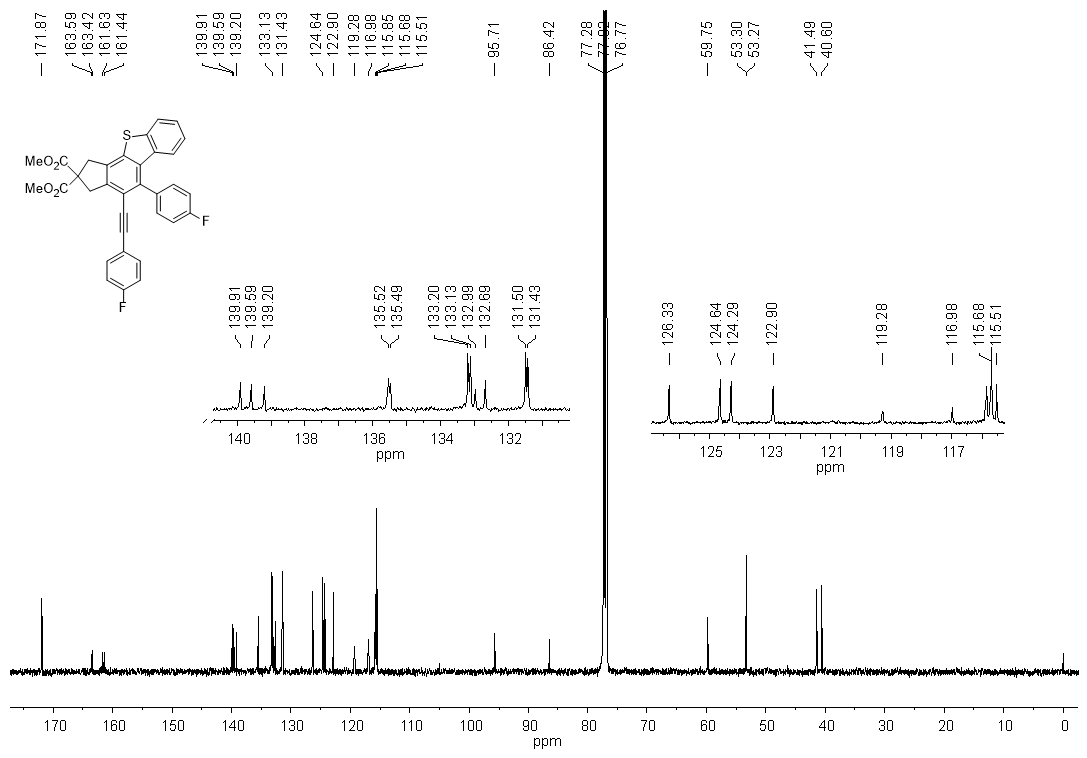


GC-MS Spectra for HPPh_2_ and Ph_2_P(O)H:


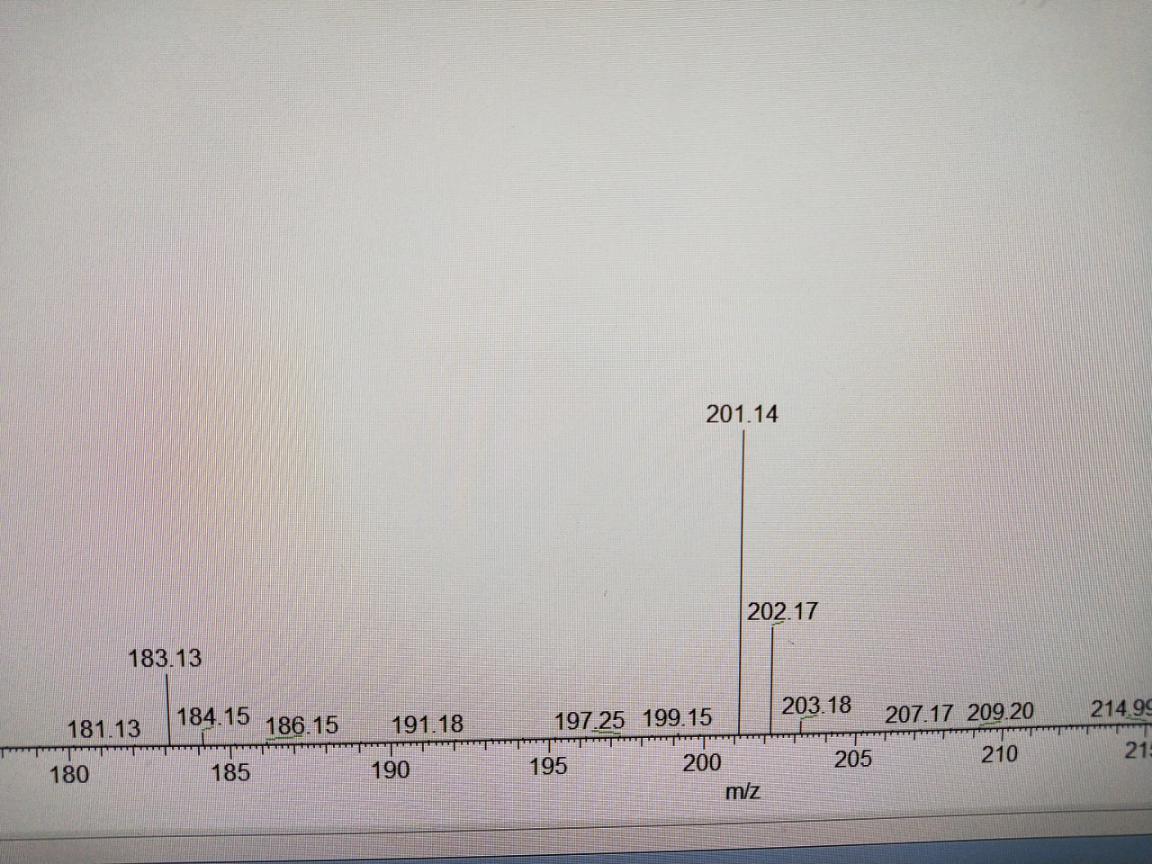


^1^H and ^31^P NMR Spectra for HPPh_2_:


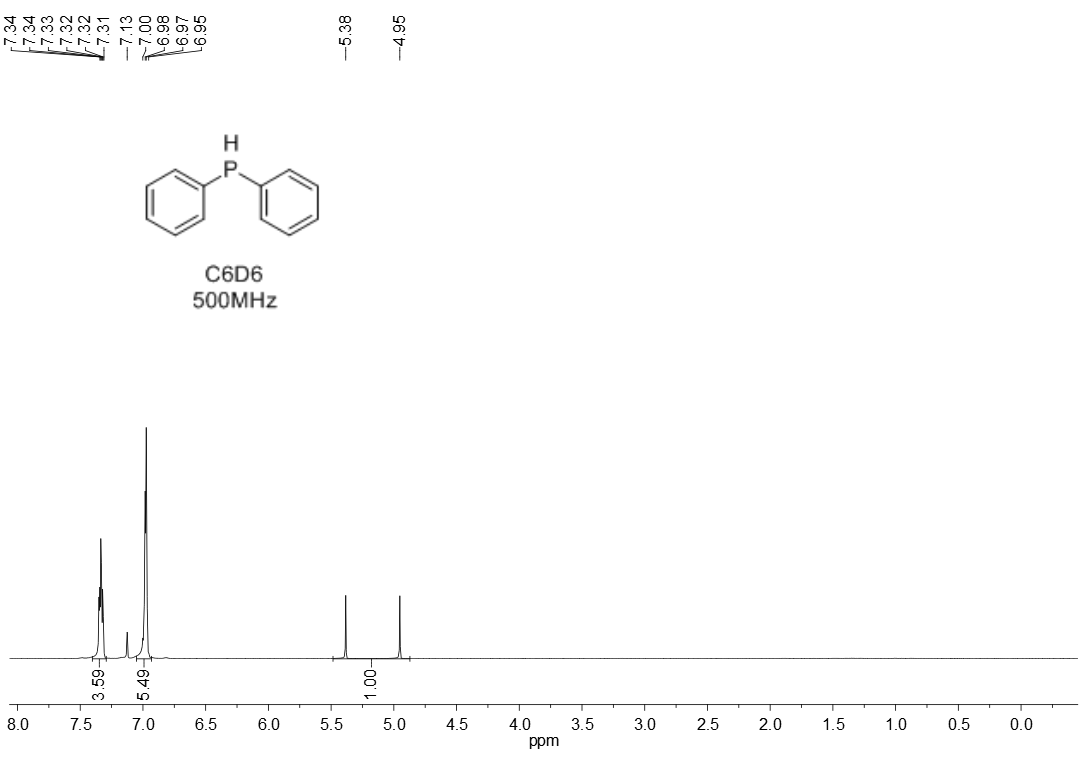


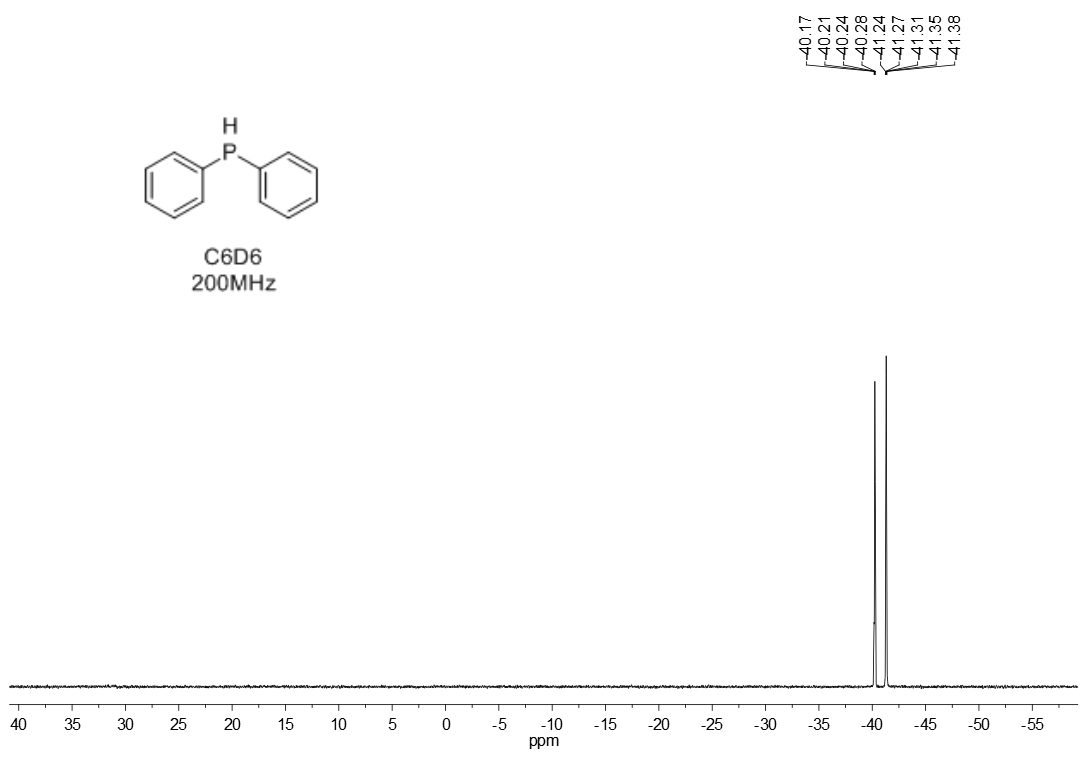


^1^H and ^31^P NMR Spectra for Ph_2_P(O)H:


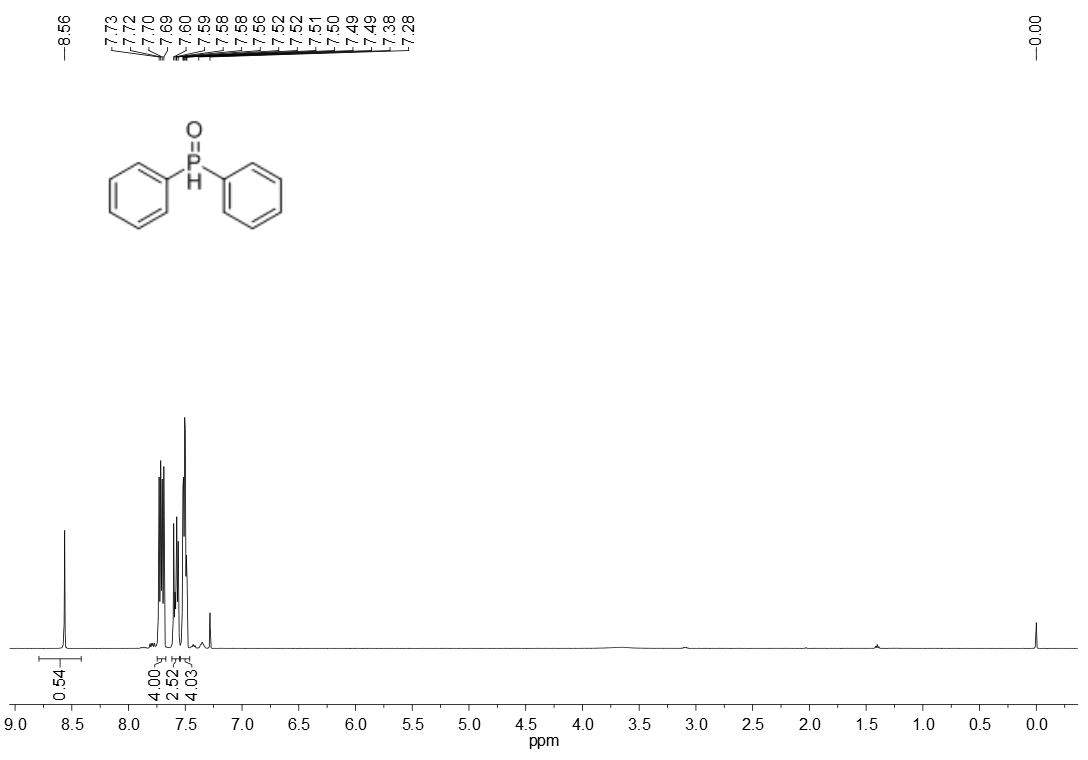

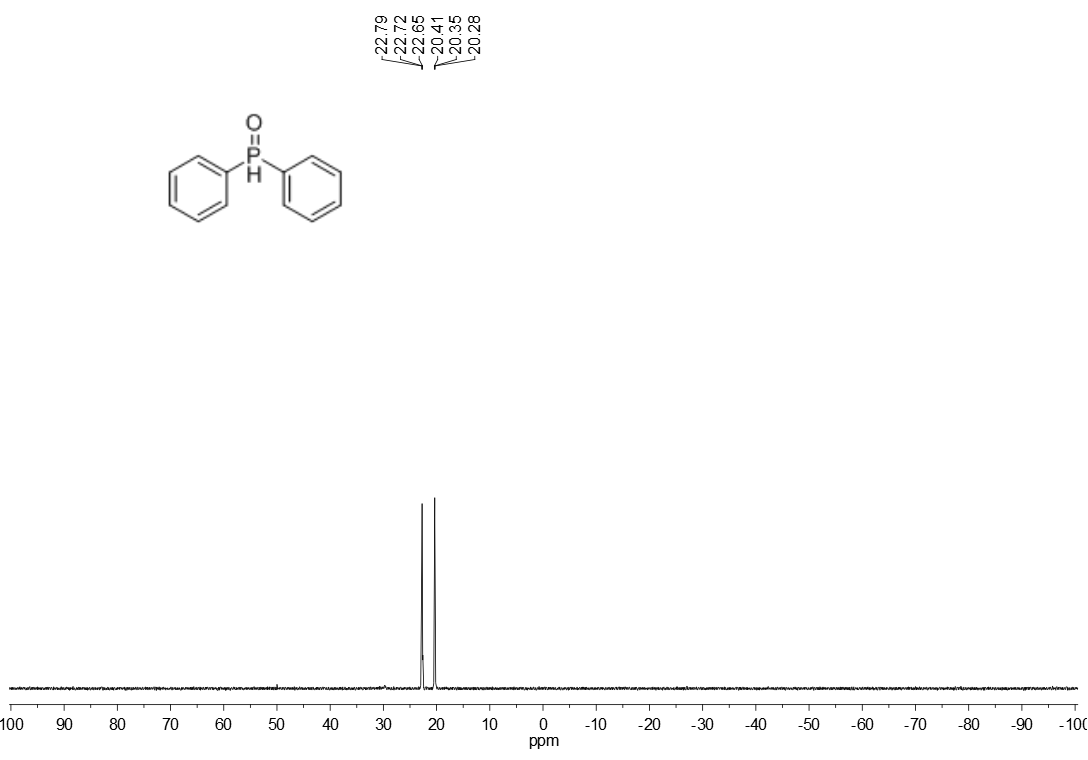

Supplement: Supplementary file 1 [file Table_1.DOC]
