# Supplementary material for: Versatile Dibenzothio[seleno]phenes via Hexadehydro-Diels–Alder Domino Cyclization
Source: Front Chem. 2019 May 24;7:374. doi: 10.3389/fchem.2019.00374 (PMC6543197; doi:10.3389/fchem.2019.00374)
Supplement: Supplementary file 11 [file Data_Sheet_10.PDF]

1. We have tried more experiments to broaden the scope of substrates. We get new products and are still working on the certain structures of the new products, we will report them in the near future as a full paper (Fig 1-4).

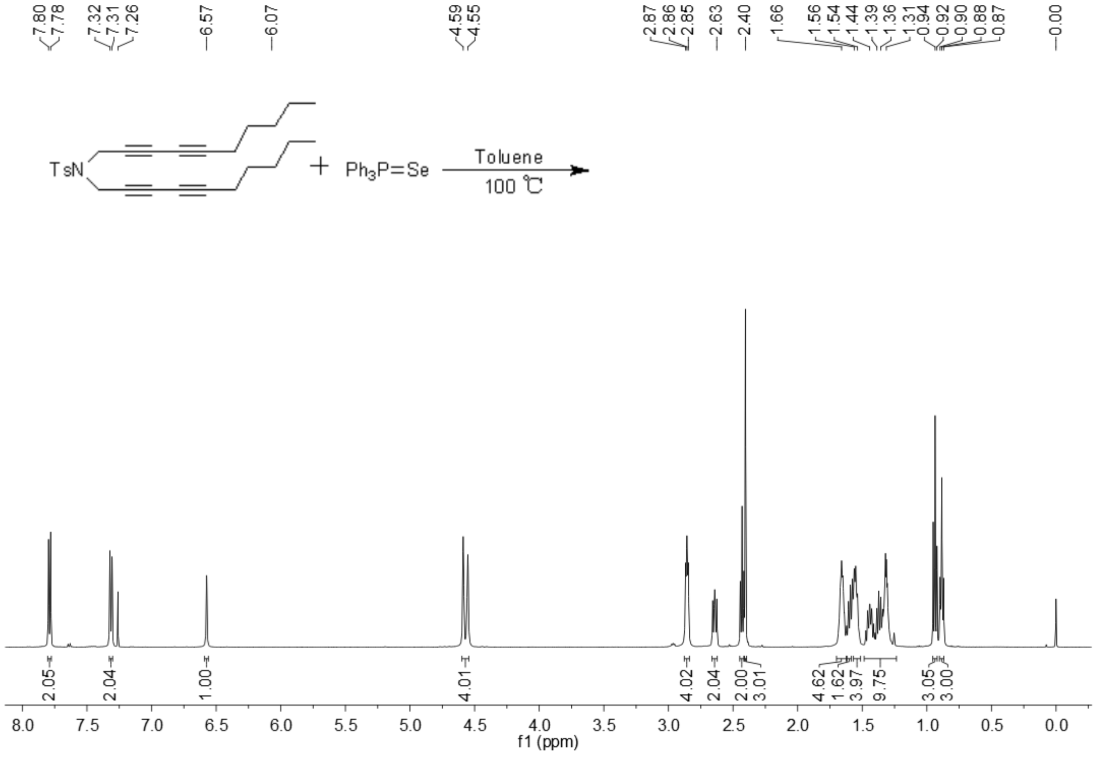

Fig 1.

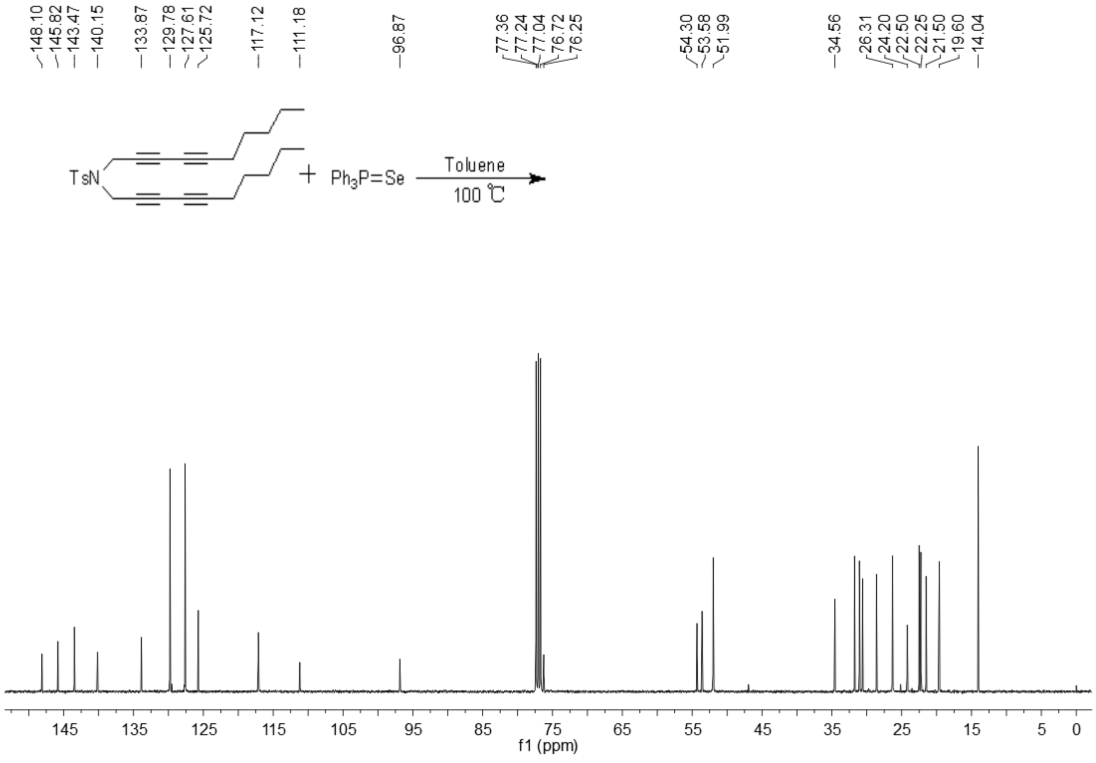

Fig 2.

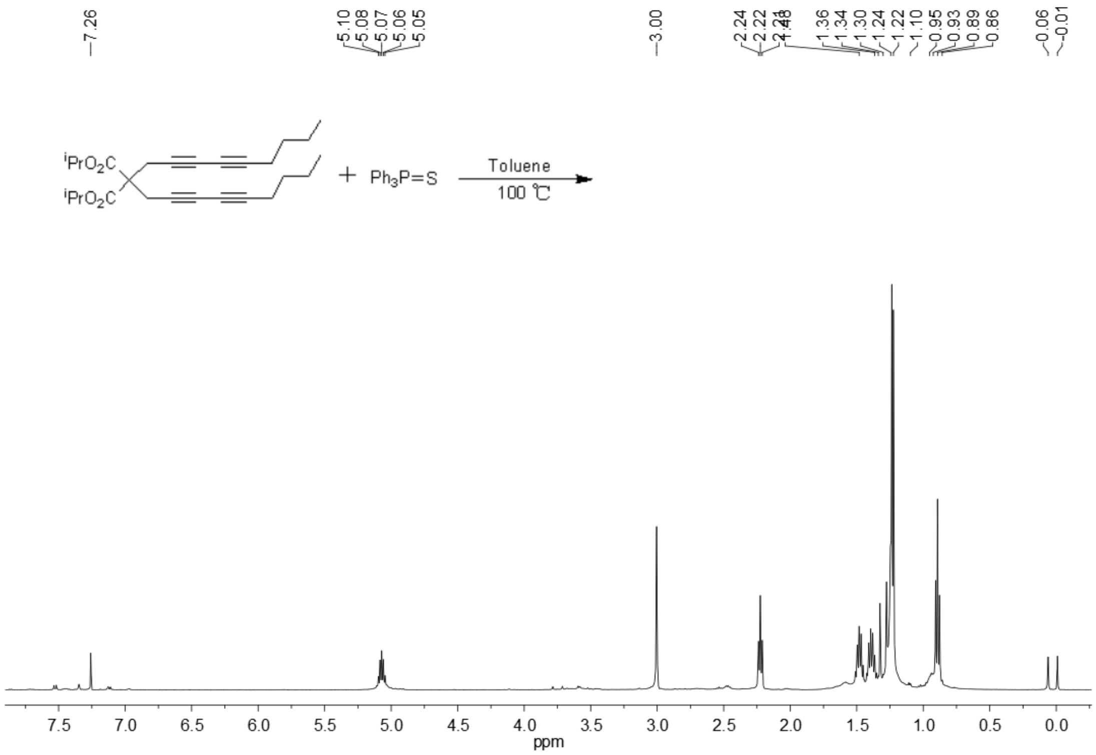
7.26
5.10
5.08
5.07
5.06
5.05
3.00
2.24
2.22
1.36
1.34
1.30
1.24
1.22
1.10
0.95
0.93
0.89
0.86
0.06
-0.01
$^iPrO_2C$
$^iPrO_2C$
$Ph_3P{=}S$
Toluene
100 ℃
ppm

Fig 3.

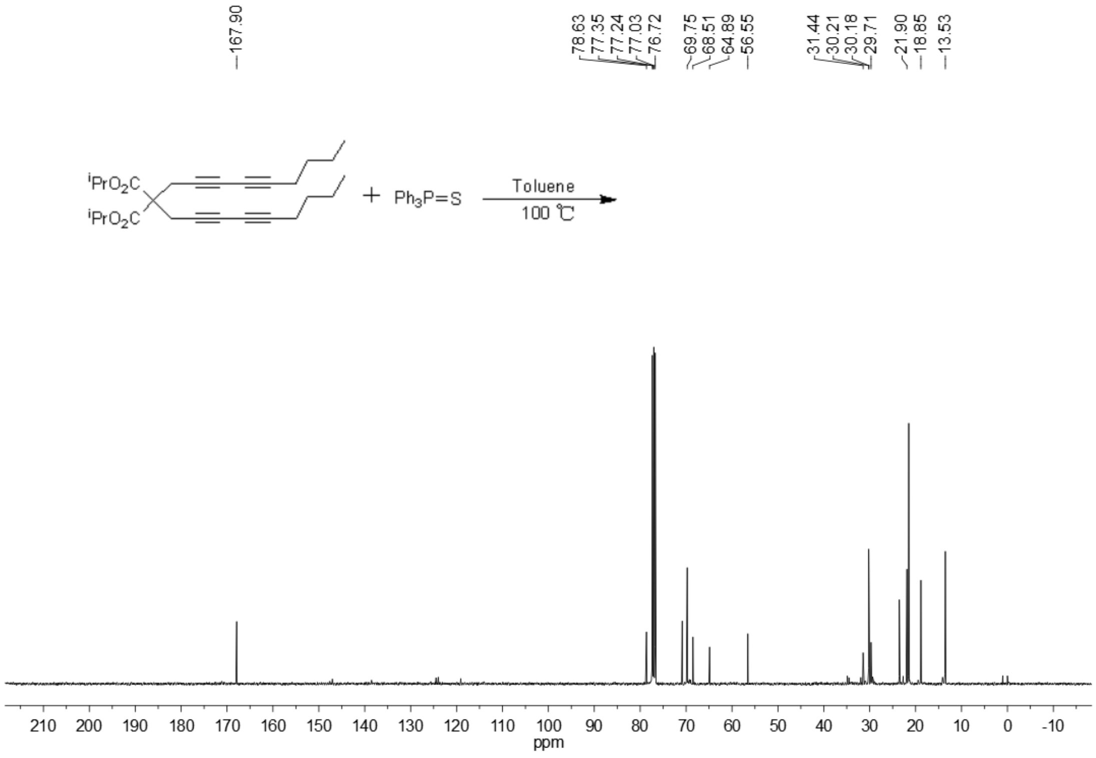
167.90
78.63
77.35
77.24
77.03
76.72
69.75
68.51
64.89
56.55
31.44
30.21
30.18
29.71
21.90
18.85
13.53
$^iPrO_2C$
$^iPrO_2C$
$Ph_3P{=}S$
Toluene
100 ℃
ppm

Fig 4.

2. ^{1}H NMR (Fig. 5) and ^{31}P NMR (Fig. 6) spectra for $HPPH_2$:

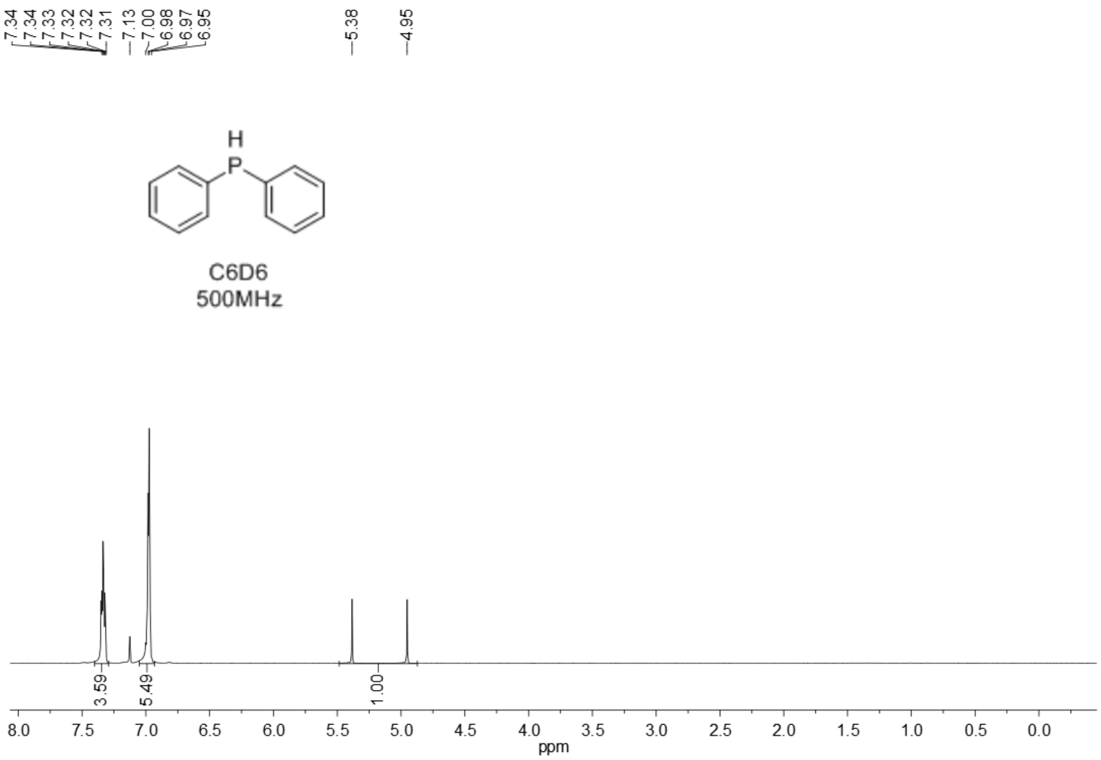

Fig 5.

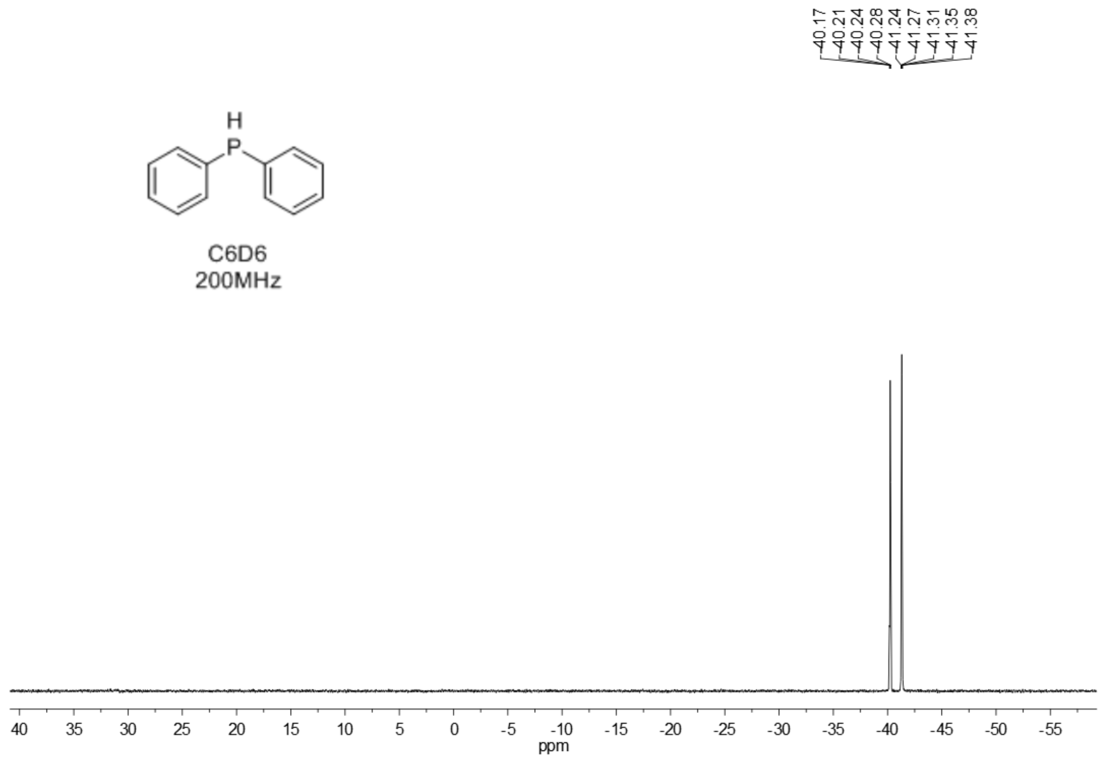

Fig 6.

3. ^{1}H NMR (Fig. 7) and ^{31}P NMR (Fig. 8) spectra for H(O)PPH2:

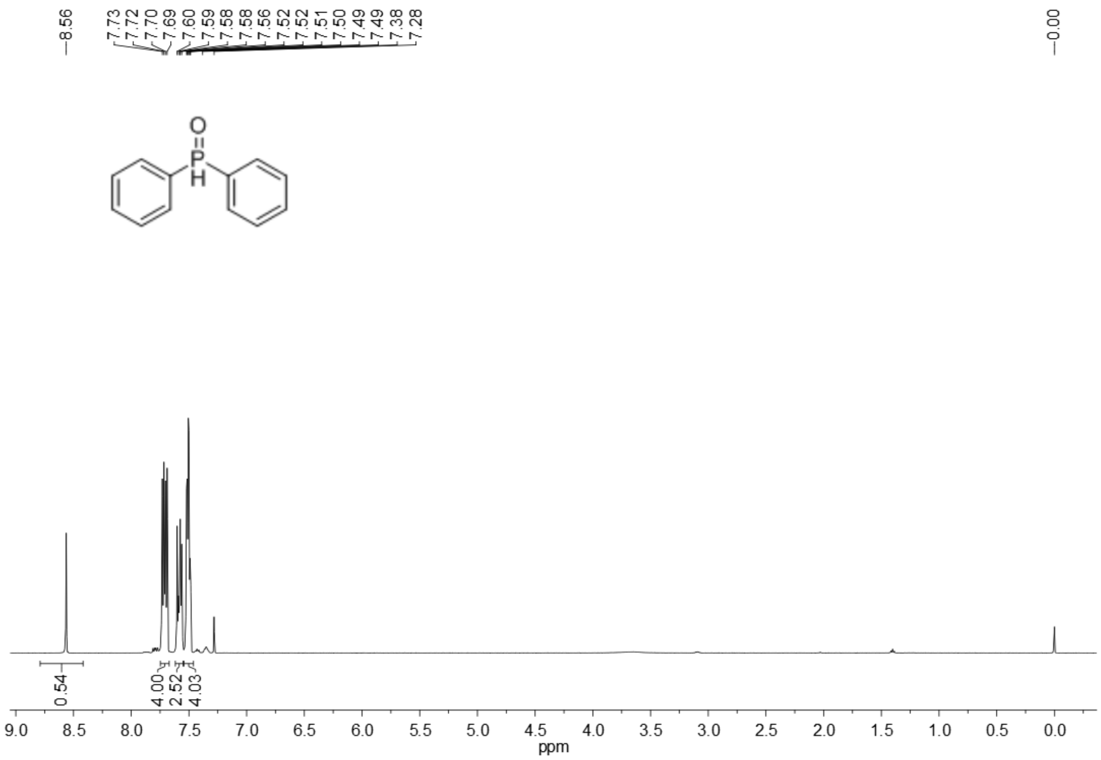

Fig 7.

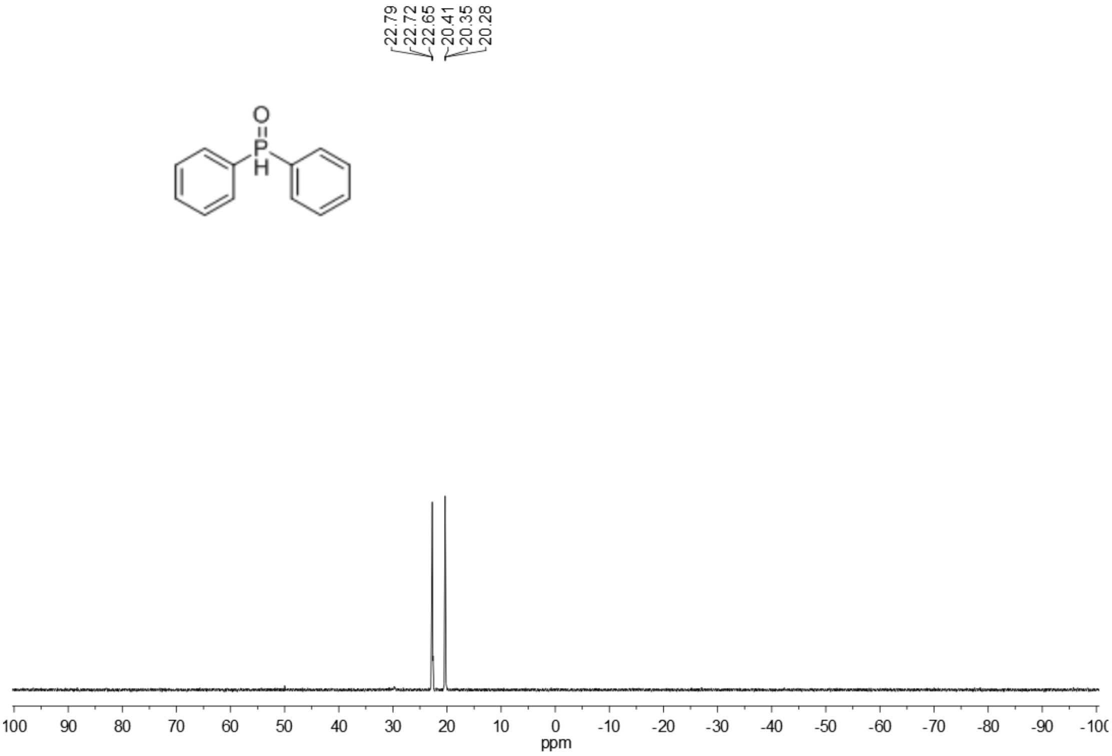

Fig 8.

4. Mechanisms of fused benzoselenophene derivatives (Fig 9, 10):

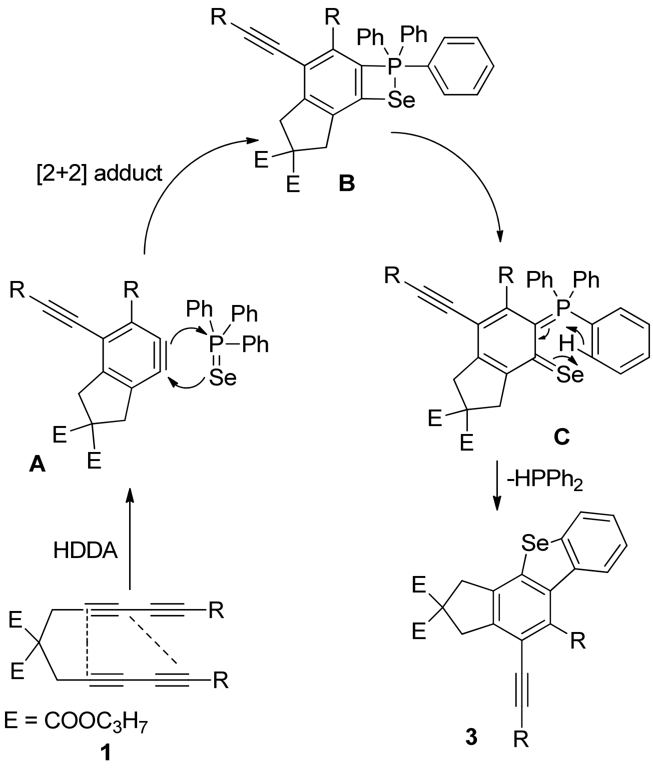

Fig 9. (Before)

Scheme 2 shows the sequence of procedures involved in the formation of Caryl–S(Se)–Caryl and C–C bonds containing benzyne cycloaddition, and an intramolecular C–H migration cascade. Aryne intermediate A, which is generated via the HDDA reaction of tetrayne 1, subsequently reacts with triphenylphosphine selenide via a formal [2+2] cycloaddition producing intermediate B.[24,25] Intermediate B is converted to intermediate C via a 4π-electrocyclic ring-opening reaction involving breaking of P–Se bonds.[26,27] The active intermediate C undergoes a 6π-electrocyclic ring-closing reaction followed by migration of an aromatic C–H bond[28] and elimination of $HPPh_2$ to afford final substituted dibenzoselenophenes and dibenzothiophenes 3. Unfortunately, diphenylphosphine was isolated from the reaction process, and this by-product was characterized by nuclear magnetic resonance and gas chromatography/mass spectrometry.

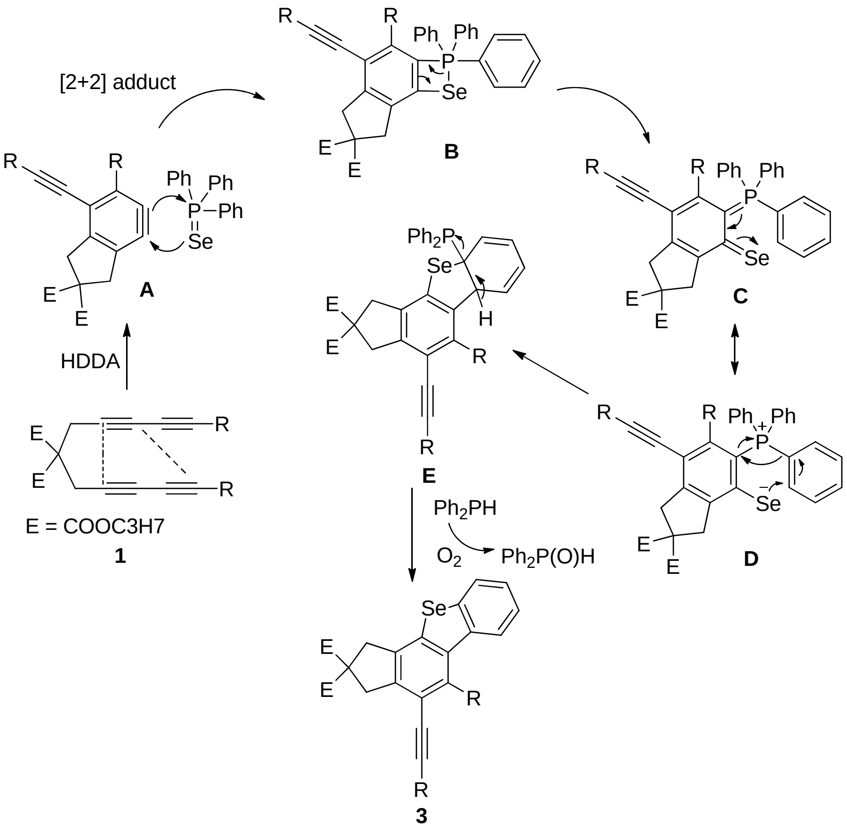

Fig 10. (Now)

Scheme 2 shows the sequence of procedures involved in the formation of Caryl–S(Se)–Caryl and C–C bonds containing benzyne cycloaddition and an intramolecular C–H migration cascade. Aryne intermediate A, which is generated via the HDDA reaction of tetrayne 1, subsequently reacts with the negative selenium ion at the acetylene carbon atoms with small steric hindrance via a nucleophilic addition reaction producing a P-Se four-membered ring intermediate B.[24,25] Intermediate B is converted to intermediate C via a 4π-electrocyclic ring-opening reaction involving the breaking of P–Se bonds.[26,27] The active intermediate C has a resonance structure D, and D then undergoes a intramolecular nucleophilic addition reaction to form E. A migration of an aromatic C-H bond[28] and elimination of $HPPh_2$ from intermediate E affords the final product 3. $HPPh_2$ was oxidized to $Ph_2P(O)H$. Fortunately, diphenylphosphine was isolated from the reaction process, and this by-product was characterized by nuclear magnetic resonance and gas chromatography/mass spectrometry.
